# Dietary Tannins in Cultured Fish: Growth Promoter or Metabolic Burden? A Meta‐Analysis

**DOI:** 10.1155/anu/2460161

**Published:** 2026-11-15

**Authors:** Vitas Atmadi Prakoso, Danung Nur Adli, Mohammad Miftakhus Sholikin, Evellin Dewi Lusiana, Rendy Ginanjar, Dita Ariyanti, Wira Wisnu Wardani, Hafizuddin Hafizuddin, Rasidi Rasidi, Frendy Ahmad Afandi, Tuty Maria Wardiny, Muhammad Ismunandar Yasin Saleh, Aliati Iswantari, Kurniawan Kurniawan, Katherine E. Doyle, Arif Wibowo, Anuraga Jayanegara

**Affiliations:** ^1^ Gulbali Institute for Agriculture, Water and Environment Charles Sturt University P.O. Box 789 Albury 2640 New South Wales Australia csu.edu.au; ^2^ Bureau of Organization and Human Resources National Research and Innovation Agency (BRIN) Cibinong Bogor 16915 West Java Indonesia brin.go.id; ^3^ Smart Livestock Industry Study Programme Department of Feed and Animal Nutrition Faculty of Animal Science Universitas Brawijaya Malang 65145 East Java Indonesia ub.ac.id; ^4^ Research Center for Animal Husbandry National Research and Innovation Agency (BRIN) Cibinong Bogor 16915 West Java Indonesia brin.go.id; ^5^ Department of Statistics Faculty of Mathematics and Natural Science Universitas Brawijaya Malang 65145 East Java Indonesia ub.ac.id; ^6^ Research Centre for Applied Zoology National Research and Innovation Agency (BRIN) Cibinong Bogor 16915 West Java Indonesia brin.go.id; ^7^ Research Centre for Applied Microbiology National Research and Innovation Agency (BRIN) Cibinong Bogor 16915 West Java Indonesia brin.go.id; ^8^ Nutricell Research and Development Division Nutricell Indonesia Cibis Nine 12th Floor Unit G1, Jl. TB Simatupang No. 2 Jakarta 12560 Indonesia; ^9^ Laboratory of Reproduction Faculty of Veterinary Medicine Universitas Syiah Kuala Banda Aceh 23111 Aceh Indonesia unsyiah.ac.id; ^10^ Research Center for Fisheries National Research and Innovation Agency (BRIN) Cibinong Bogor 16915 West Java Indonesia brin.go.id; ^11^ Bureau of Performance Management and Cooperation Coordinating Ministry for Economic Affairs Republic of Indonesia Jakarta 10710 Indonesia; ^12^ Agribusiness Study Program Universitas Terbuka South Tangerang 15437 Banten Indonesia; ^13^ Department of Marine Affairs and Fisheries West Sulawesi Provincial Government Mamuju 91511 West Sulawesi Indonesia; ^14^ Department of Aquatic Resources Management Faculty of Fisheries and Marine Sciences IPB University Bogor 16680 West Java Indonesia ipb.ac.id; ^15^ Research Centre for Biota System National Research and Innovation Agency (BRIN) Cibinong Bogor 16915 West Java Indonesia brin.go.id; ^16^ Department of Nutrition and Feed Technology Faculty of Animal Science IPB University Bogor 16680 West Java Indonesia ipb.ac.id

**Keywords:** antioxidant response, digestibility, functional feed additives, gut histology, immune modulation, polyphenols

## Abstract

Tannins are being increasingly explored as functional feed additives in aquaculture; however, their effects remain context‐dependent. This meta‐analysis assessed the impact of dietary tannin supplementation on growth, nutrient digestibility, physiological stress, immunity and gut morphology in cultured fish. A total of 97 studies were included, and standardised mean differences (Hedges’ *g*) were calculated for various growth performance and health parameters. Subgroup analyses were conducted according to tannin category, trophic guild, habitat and fish species. In general, tannin supplementation negatively affected several aspects of growth and nutrient utilisation, including feed consumption, protein utilisation, nutrient digestibility, digestive enzyme activity and lipid deposition, together with poorer feed efficiency as reflected by increased feed conversion ratio (FCR) in several major sub‐groups. The negative impacts of tannins were more pronounced for fish cultured in freshwater and those classified as either omnivores or herbivores, while there was less consistency in the degree to which growth reduction was produced by tannins among carnivorous species. Dose‐response analysis identified significant relationships for several key outcomes, although tannin inclusion level explained only a modest proportion of the observed variation, and no general optimum inclusion level was identified for growth responses. However, even though there was little evidence to suggest that survival was negatively impacted, this suggests that tannins acted primarily as sub‐lethal nutritional stressors rather than acute toxicants. While levels of certain antioxidants and markers of immune function tended to increase in several sub‐groups, the magnitude of such increases should be viewed cautiously, since they occurred alongside impaired growth and nutrient utilisation. Therefore, tannins should not be recommended using a simple inclusion‐level threshold; their application in aquafeeds should be evaluated according to tannin category, botanical source, analysed tannin concentration, fish species, feeding guild, habitat, basal diet composition and digestibility responses.

## 1. Introduction

Tannins are natural plant polyphenols with significant antimicrobial effects. Because of their impact on the growth and well‐being of fish, they have become a point of interest for the use in aquaculture. These compounds occur in a wide variety of food sources, including fruits, vegetables, legumes and grains [1, 2] and can be broadly categorised by structural characteristics as either hydrolysable tannins or condensed tannins [1, 3]. Since the early 1990s, research interest in tannins has grown greatly because tannins display a variety of biological activities, such as being antioxidants, antimicrobials and anti‐inflammatories [4–6].

In aquaculture nutrition, dietary tannins may occur naturally in plant‐based feed ingredients, be supplied through tannin‐rich plant materials used as dietary ingredients, or be intentionally supplemented as purified or extracted compounds. This distinction is important because the biological effects of tannins depend not only on their dietary concentration but also on their chemical characteristics, botanical source and the composition of the basal diet. Similar context‐dependent responses to tannin‐containing feeds and tannin supplementation have also been reported in other animal‐production systems [7–9]. In cultured fish, tannins may provide antioxidant, antimicrobial and immunomodulatory effects [2, 6, 10], but they may also impair nutritional performance when supplied at unsuitable concentrations or in highly reactive forms. These adverse effects are primarily related to their ability to bind with dietary proteins, amino acids and minerals, forming non‐digestible or insoluble complexes and therefore limiting nutrient availability and/or inhibiting growth performance in cultured fish [11, 12].

Among these mechanisms, interactions between tannins and dietary proteins may help explain why some tannin‐containing diets impair growth, whereas others have little apparent effect. Tannins interact strongly with proteins in dietary ingredients and can form insoluble complexes [13]. This binding process may lead to reduced protein digestibility [1, 14] and limited utilisation of nutrients from the diet [13]. A few studies have demonstrated that the structural characteristics of tannins can directly impact protein binding/interactions, utilisation of nutrients and overall growth performance in cultured fish [15, 16]. The strength of these interactions can vary with tannin structure and molecular characteristics, which influence tannin reactivity and affinity for proteins [17]. Structural variation can also influence interactions with digestive enzymes, providing an additional mechanism through which tannins may affect nutrient digestion and utilisation [18]. Nevertheless, the interactions between tannins and dietary proteins may not always inhibit growth under all dietary conditions. For example, this effect may vary based upon tannin category, tannin inclusion, fish species and composition of the basal diet. Importantly, responses to dietary tannin inclusion may vary across tannin sources, diets and fish species, and the available evidence does not support a single threshold or optimal inclusion level that can be applied broadly across aquaculture species [15, 19, 20]. Different inclusion levels may produce neutral or potentially beneficial, neutral or adverse responses depending on the tannin source and biological context, highlighting the need to evaluate dose–response relationships within specific species and dietary conditions. Similar context‐dependent responses to dietary feed additives have also been reported in previous aquaculture meta‐analyses [21, 22]. A number of interdependent variables have been identified to affect how well tannins perform as an additive in aquafeed, including tannin category, tannin source, extraction methodology used to obtain tannins from the plant material, chemical structure of the extracted tannins and the quantity of each type of tannin added to the aquafeed [13, 23]. The complexity of interpreting tannin efficacy is increased further by species‐specific differences in feeding strategy, digestive tract morphology, endogenous enzyme profiles and sensitivity or adaptation to plant‐derived secondary metabolites [16]. Carnivorous, omnivorous and herbivorous fish may therefore respond differently to dietary tannins because they differ in their habitual exposure to plant‐derived compounds, digestive tract characteristics and endogenous digestive enzyme profiles. Dietary tannins may also influence the intestinal microbiota. For example, hydrolysable tannin supplementation altered microbial diversity and the relative abundance of several intestinal bacterial taxa in grass carp [24]. Finally, hydrolysable and condensed tannins may elicit different physiological responses in animals fed these types of diets, and these broad categories encompass substantial chemical diversity, complicating comparisons across studies where little detail is provided about the chemistry of the tannins studied [1, 11].

Despite the many positive biological activities of extracts of tannins in animal feeds, there are numerous inconsistencies in experimental results obtained using cultured fish. Many reports indicate that certain levels of tannin extract improve growth performance, stimulate immune systems and/or provide protection against diseases. Conversely, many studies report no difference in fish growth or immune stimulation when compared to control animals receiving no supplement [10, 11, 15]. Such variability suggests that successful application of tannins may require knowledge of both nutritional and biological conditions that presently remain poorly defined. Consequently, we conducted a meta‐analysis of the published literature concerning the efficacy of tannin supplements in diets for cultured fish. Specifically, our objective was to synthesise data collected in previous studies regarding the impacts on growth performance, feed utilisation, nutrient digestibility and physiological and immune‐related responses resulting from tannin supplementation in cultured fish and to determine how these responses vary according to tannin category, inclusion level, trophic classification, habitat and fish species.

## 2. Materials and Methods

### 2.1. Literature Review and Data Collection

This study was conducted and reported in accordance with the PRISMA 2020 guidelines [25], consistent with their application in recent systematic reviews and meta‐analysis in animal nutrition and production research [26, 27]. A systematic literature search was conducted in the Web of Science and Scopus databases on 27 January 2026. In Web of Science, the search was conducted within the title, abstract, and keyword fields using (((TS = tannin ^∗^) AND (TS = fish ^∗^)) AND ((TS = feed ^∗^) OR (TS = diet ^∗^))). In Scopus, the search was conducted using TITLE‐ABS‐KEY ("tannin” AND “fish” AND ("feed” OR “diet")). Only English‐language studies were considered eligible for inclusion. No publication‐type restriction was applied at the database‐search stage. Therefore, non‐journal records indexed in these databases, including conference proceedings and book chapters, were retained for screening when retrieved. Records were subsequently assessed against the predefined eligibility criteria, with non‐primary studies and other ineligible records being excluded during screening.

Studies were included in the meta‐analysis database if they met the following criteria: (i) the control diet contained fish meal as a basal protein source and did not include supplemental tannin; (ii) the experimental diet included dietary tannin supplementation; and (iii) the study evaluated the effects of tannin supplementation on fish growth, feed utilisation or health‐related parameters. The restriction to fish meal‐containing control diets was applied to provide a more consistent nutritional baseline, reduce heterogeneity arising from major differences in basal protein sources and improve comparability among studies. From each eligible study, the following variables were extracted: study design, tannin inclusion level, tannin category (such as condensed, hydrolysable, or mixed), trophic classification, habitat, species, fish size, growth and feed‐utilisation parameters and physiological parameters together with their associated standard deviations.

The databases yielded 379 articles in total after removing duplicates. Following title and abstract screening, 206 articles were retained. After full‐text assessment, 91 articles were excluded for irrelevance, resulting in a total of 115 articles included in further full‐text assessment. Final assessment resulting in a total of 88 articles included for meta‐analysis (Figure 1). For transparency, the articles excluded after full‐text assessment and the corresponding reasons for exclusion are provided in Table S1.

**Figure 1:**
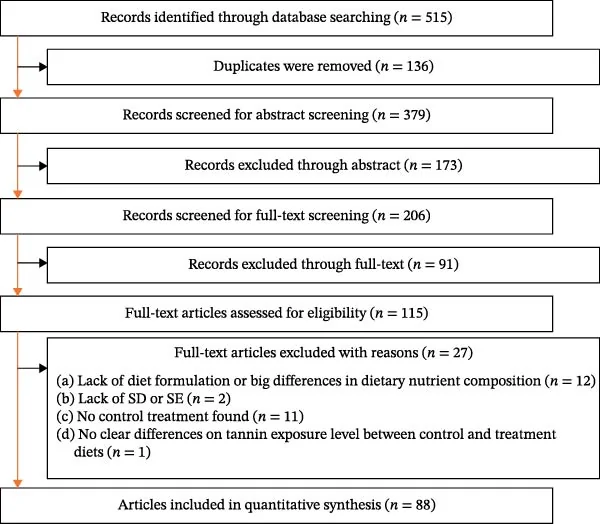
PRISMA flow diagram showing the identification, screening, eligibility assessment and inclusion of articles on the effects of dietary tannins on fish growth and health.

### 2.2. Data Extraction

A total of 97 studies from 88 articles were included in the final analysis. An ’article’ refers to a single publication, whereas a ‘study’ represents a distinct experiment reported within an article. Multiple tannin inclusion levels within the same experiment remained under the same Study ID and were analysed as separate treatment‐control contrasts, with the Study ID retained as the experimental unit. Each Study ID represented a single fish species. Each study reported various aspects, including fish species, trophic feeding behaviour (carnivore, omnivore and herbivore), habitat (freshwater and marine), initial body weight, life stage, source of tannin, tannin category (condensed, hydrolysable, mixed and not specified) and the proportion of dietary tannins administered in the experimental diet (ranging from 0.002% to 17.4%). Life stage was classified using terminology reported in the original studies where available, supplemented by species‐specific body‐size context when necessary. Cases that could not be classified confidently were recorded as not specified. Initial body weight was also recorded as a study characteristic (Table S2). Data were independently extracted by two reviewers, and any discrepancies were resolved through discussion and consensus. Where numerical data were presented only graphically, values were extracted from figures using the web‐based GraphReader tool (GraphReader, https://www.graphreader.com).

### 2.3. Risk‐of‐Bias (RoB) Assessment

All 97 selected studies underwent a methodological quality assessment using a RoB framework based on Sterne et al. [28]. The framework was adapted to the context of aquaculture nutrition experiments. The assessment was conducted independently by two reviewers. Five domains were evaluated: (D1) potential bias in the randomisation process, (D2) deviations from the intended interventions, (D3) incomplete outcome data, (D4) inaccuracies in outcome measurement and (D5) selective reporting of outcomes. These domains were interpreted with consideration of aquaculture‐specific study features, including allocation of fish or experimental units, implementation of dietary treatments and husbandry conditions, completeness of reported outcomes, reliability of measurement procedures and selective omission or reporting of measured outcomes. Each domain was rated as ‘Cannot determine’, ‘Low risk’, Some concerns’ or ‘High risk’ (Figure 2). The RoB was assessed separately across the five domains. Figure 2 presents the study‐level ratings for each domain and the corresponding summary distribution across all 97 studies.

**Figure 2:**
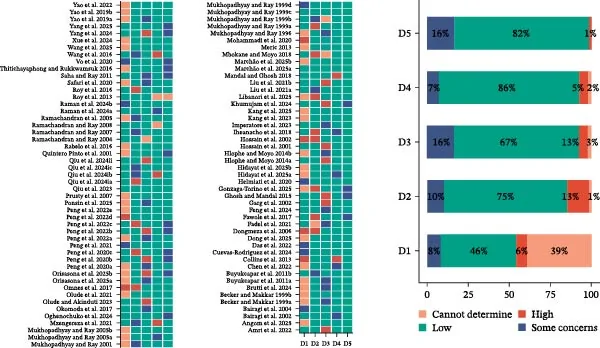
Risk‐of‐bias assessment across studies. The left panel shows study‐level risk of bias ratings across the five domains, while the right panel summarises the percentage distribution of ‘Cannot determine’, ‘Low’, ‘Some concerns’ and ‘High’ ratings within each domain across all studies. The five domains were (D1) potential bias in the randomisation process, (D2) deviations from the intended interventions, (D3) incomplete outcome data, (D4) inaccuracies in outcome measurement and (D5) selective reporting of outcomes.

### 2.4. Analysis of Data and Validation

Treatment effects were analysed through a comparison meta‐analytic framework whereby fish fed tannin‐supplemented diets were compared with fish fed the corresponding control‐diet without tannin inclusion. For each comparison, effect sizes as standardised mean differences (Hedges’ *g*) were calculated based on group means, standard deviations and sample size for the treatment and control group. Positive effect sizes indicate a higher value in the treatment group relative to the control, and negative effect sizes correspond to lower values. Since true effects were expected to vary among studies (due to differences in fish species, tannin inclusion level, tannin category, experimental conditions and diet composition), all analyses were performed with random‐effects models. Each effect size was weighted by the inverse of its sampling variance, and between‐study heterogeneity was accounted for by the random‐effects variance component. Between‐study heterogeneity was assessed using Cochran’s *Q*, *I^2^* and *τ^2^*, with *τ^2^* estimated using the DerSimonian‐Laird method. Pooled effect estimates were reported with 95% confidence intervals, and 95% prediction intervals were also calculated. In addition, quadratic regression was used to examine potential non‐linear relationships between the tannin inclusion level and the measured response variables. Predefined subgroup analyses were done according to the tannin category, trophic classification, habitat and species. If a study reported multiple tannin inclusion levels, each treatment‐control contrast was extracted as a separate effect size while remaining associated with the same study ID. If multiple tannin treatments shared a common control group, the sample size of the shared control was divided evenly among the corresponding comparisons to reduce unit‐of‐analysis error and avoid overweighting the shared control in the meta‐analysis. This adjustment reduces the influence of repeatedly used control groups while recognising that some within‐study correlation may remain among effect sizes derived from the same study. To maximise comparability among studies, only studies using fish meal‐containing control diets were included. The remaining variation in basal diet composition and feed ingredients among studies was treated as a source of residual heterogeneity under the random‐effects framework. We followed standard conventions of meta‐analysis to perform our analysis and to communicate our findings, but refer to Table S3 for the equations and variables underlying the statistical calculations of our results. Potential publication bias and small‐study effects were assessed using Begg’s test and Egger’s regression, with fail‐safe numbers (FSNs) also calculated to evaluate the robustness of the pooled estimates. Data were processed and visualised in RStudio (ver. 2025.05.1 + 513, ‘Mariposa Orchid’ release for Windows; Posit Software, PBC, Boston, MA, USA), using the following packages: ggplot2 (ver. 4.0.0), tidyr (ver. 1.3.1), dplyr (ver. 1.1.4), readxl (ver. 1.4.5), plotly (ver. 4.11.0), htmlwidgets (ver. 1.6.4), patchwork (ver. 1.3.2) and cowplot (ver. 1.2.0). Statistical significance was indicated when *p* < 0.05.

## 3. Results

Based on the life‐stage classification applied in this study, most experiments were classified as involving juvenile/fingerling fish (91 of 97 studies), whereas relatively few involved grow‐out (*n* = 2) or adult fish/broodstock (*n* = 1), and life stage could not be assigned confidently for three studies. Initial body weight varied widely across the included studies and species (Table S2). Quadratic regression showed significant relationships between the tannin inclusion level and weight gain (WGN), apparent protein digestibility (APD) and apparent net protein utilisation (ANP) (Figure 3). WGN showed a significant U‐shaped relationship with tannin inclusion level (*R* ^2^ = 0.0465, *p* = 0.0008), although no optimum point was identified within the fitted range. In contrast, specific growth rate (SGR) showed no significant quadratic relationship with tannin inclusion level (*R* ^2^ = 0.0113, *p* = 0.2006), with no optimum point identified within the fitted range. APD showed a significant quadratic relationship (*R* ^2^ = 0.0606, *p* = 0.0227), with the fitted curve reaching a maximum at ~1.39% tannin inclusion (APD = 86.41%). ANP also showed a significant quadratic relationship (*R* ^2^ = 0.0610, *p* = 0.0444), with a predicted maximum at ~0.88% tannin inclusion (ANP = 36.38%). In contrast, no significant quadratic relationships were detected for SGR (*R* ^2^ = 0.0113, *p* = 0.2006), feed conversion ratio (FCR; *R* ^2^ = 0.0099, *p* = 0.2483), survival rate (SUR; *R* ^2^ = 0.0377, *p* = 0.1058) or crude lipid content (CLC; *R* ^2^ = 0.0120, *p* = 0.2870).

Figure 3Quadratic regression on the effects of dietary tannin inclusion on (a) specific growth rate (SGR), (b) weight gain (WGN), (c) feed conversion ratio (FCR), (d) survival rate (SUR), (e) apparent net protein utilisation (ANP), (f) apparent protein digestibility (APD) and (g) crude lipid content (CLC) in cultured fish. *R* ^2^ and *p*‐values are shown for each model, with estimated optimum inclusion levels indicated where identifiable within the fitted range.

**Figure:**
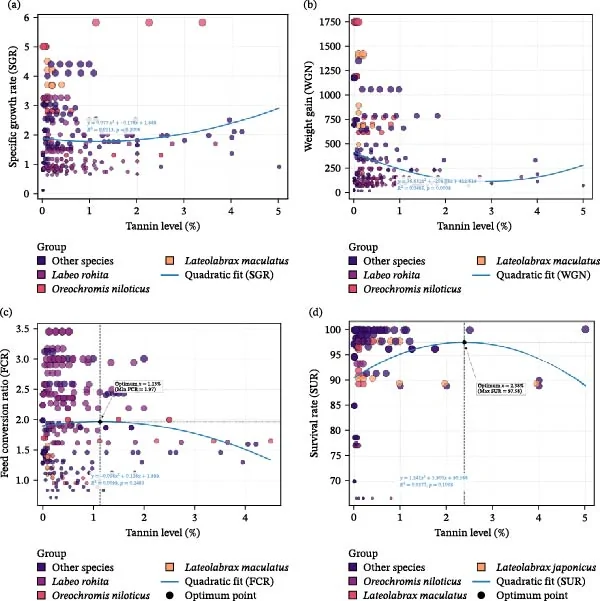

**Figure:**
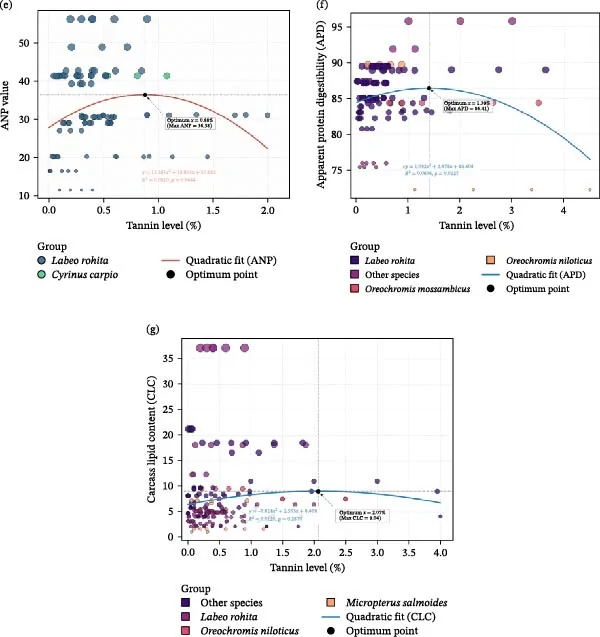

The effects of the tannin category on cultured fish performance were evaluated (Table 1 for key outcomes; Table S4 for the complete subgroup analysis). Of the 97 included studies, only three (3.1%) were classified as ‘not specified’ for the tannin category. Fish fed the diet with hydrolysable tannins had a mean SGR of *g* = –1.12; 95% CI: –1.64 to –0.59; *p* < 0.001 and those fed the diet with condensed tannins had a mean SGR of *g* = –1.05; 95% CI: –1.42 to –0.67; *p* < 0.001. In addition to their negative impact on a mean SGR, both tannin category also negatively impacted WGN. FCR values increased significantly under condensed tannins (*g* = 1.39, 95% CI: 1.07–1.70, *p* < 0.001) and hydrolysable tannins (*g* = 0.80, 95% CI: 0.35–1.25, *p* < 0.001). Protein efficiency ratio was significantly reduced by hydrolysable tannins (*g* = −1.63, 95% CI: −2.40 to −0.87, *p* < 0.001) and condensed tannins (*g* = −1.10, 95% CI: −1.38 to −0.83, *p* < 0.001). These growth responses were accompanied by significant reductions in body CLC under condensed tannins (*g* = −1.04, 95% CI: −1.40 to −0.68, *p*  < 0.001) and hydrolysable tannins (*g* = −0.85, 95% CI: −1.41 to −0.29, *p*  < 0.001), whereas body crude protein, body ash, and SUR were not significantly affected (*p*  > 0.05). Additionally, condensed tannins significantly reduced APD (*g* = −1.48, 95% CI: −1.89 to −1.06, *p*  < 0.001), apparent lipid digestibility (*g* = −0.84, 95% CI: −1.30 to −0.38, *p*  < 0.001), apparent ash digestibility (*g* = −1.63, 95% CI: −2.92 to −0.35, *p* = 0.01) and apparent phosphorus digestibility (*g* = −1.81, 95% CI: −3.25 to −0.38, *p* = 0.01), while hydrolysable tannins strongly reduced APD (*g* = −4.89, 95% CI: −7.16 to −2.62, *p*  < 0.001). Digestive enzyme activities were also significantly reduced under condensed tannins, including amylase (*g* = −1.09, *p*  < 0.001), lipase (*g* = −0.86, *p* = 0.01), protease (*g* = −1.20, *p*  < 0.001) and pepsin (*g* = −1.59, *p* = 0.02). In contrast, several antioxidant and immune‐related parameters increased significantly, including total antioxidant capacity under condensed tannins (*g* = 0.73, *p* = 0.01), superoxide dismutase (*g* = 0.92, *p*  < 0.001), catalase under hydrolysable (*g* = 1.24, *p*  < 0.001) and condensed tannins (*g* = 0.94, *p*  < 0.001), lysozyme activity under condensed (*g* = 1.27, *p*  < 0.001) and hydrolysable tannins (*g* = 1.82, *p* = 0.01), white blood cells under condensed tannins (*g* = 1.23, *p*  < 0.001), leukocytes under condensed (*g* = 1.39, *p* = 0.01) and hydrolysable tannins (*g* = 5.96, *p*  < 0.001), and immune globulin under condensed tannins (*g* = 0.69, *p* = 0.02). However, there was high heterogeneity across many parameters, frequently with *I^2^* values exceeding 70%, with corresponding between‐study variance estimates (*τ* ^2^ and *τ* ^2^′) also varying substantially among outcomes and tannin categories.

**Table 1:** Effects of dietary tannin supplementation on key outcomes in cultured fish according to tannin category.

Parameter	Subgroup	*k*	*g*	SE	*p*	Lower	Upper	FsN (*R*)	*I* ^*2*^	*τ^2^*	*τ^2^′*	*Q*	Begg	Egger
Specific growth rate (%g/day)	Hydrolysable	67	−1.12	0.27	<0.001	−1.64	−0.59	138.54	78.98	2.79	3.55	<0.001	<0.001	<0.001
Specific growth rate (%g/day)	Condensed	230	−1.46	0.17	<0.001	−1.79	−1.14	182.65	81.16	4.36	5.68	<0.001	<0.001	<0.001
Feed conversion ratio	Condensed	224	1.39	0.16	<0.001	1.07	1.70	223.67	80.07	3.94	5.18	<0.001	<0.001	<0.001
Feed conversion ratio	Hydrolysable	58	0.80	0.23	<0.001	0.35	1.25	287.72	72.92	1.72	2.22	<0.001	<0.001	<0.001
Protein efficiency ratio	Hydrolysable	40	−1.63	0.39	<0.001	−2.40	−0.87	17.56	72.77	3.75	4.98	<0.001	<0.001	<0.001
Protein efficiency ratio	Condensed	194	−1.10	0.14	<0.001	−1.38	−0.83	419.25	73.89	2.51	3.35	<0.001	<0.001	<0.001
Weight gain (%)	Hydrolysable	68	−1.40	0.35	<0.001	−2.08	−0.71	65.63	79.38	4.86	6.27	<0.001	<0.001	<0.001
Weight gain (%)	Condensed	227	−1.05	0.19	<0.001	−1.42	−0.67	567.19	84.10	5.75	7.87	<0.001	<0.001	<0.001
Body crude protein content (%)	Condensed	177	−0.15	0.16	0.34	−0.47	0.16	28,865.63	78.71	3.14	3.25	<0.001	0.28	0.05
Body crude protein content (%)	Hydrolysable	48	−0.23	0.24	0.34	−0.69	0.24	3572.23	62.32	1.59	1.81	<0.001	0.19	0.42
Body crude lipid content (%)	Condensed	161	−1.04	0.18	<0.001	−1.40	−0.68	413.27	80.85	3.91	4.94	<0.001	<0.001	<0.001
Body crude lipid content (%)	Hydrolysable	48	−0.85	0.29	<0.001	−1.41	−0.29	208.47	69.86	2.58	2.58	<0.001	<0.001	0.49
Apparent protein digestibility (%)	Condensed	109	−1.48	0.21	<0.001	−1.89	−1.06	82.99	77.15	3.36	5.03	<0.001	<0.001	<0.001
Apparent protein digestibility (%)	Hydrolysable	15	−4.89	1.16	<0.001	−7.16	−2.62	−12.59	78.19	12.74	15.92	<0.001	<0.001	<0.001
Apparent lipid digestibility (%)	Condensed	45	−0.84	0.24	<0.001	−1.30	−0.38	198.03	59.44	2.21	3.54	<0.001	<0.001	<0.001
Survival rate (%)	Hydrolysable	33	0.01	0.21	0.98	−0.41	0.42	3,866,868.37	52.30	0.75	0.75	<0.001	0.29	0.10
Survival rate (%)	Condensed	82	<0.001	0.09	1.00	−0.18	0.18	1,490,197,010	12.54	0.09	0.09	0.18	0.02	<0.001
Feed intake (g/fish/day)	Hydrolysable	19	−1.79	0.38	<0.001	−2.54	−1.05	3.66	61.75	1.58	2.78	<0.001	<0.001	<0.001
Feed intake (g/fish/day)	Condensed	71	−0.59	0.21	<0.001	−0.99	−0.18	717.66	70.37	1.97	2.22	<0.001	<0.001	<0.001
Total antioxidant capacity (U/mL)	Condensed	37	0.73	0.28	0.01	0.19	1.27	229.29	71.94	1.95	2.31	<0.001	0.06	0.07
Total antioxidant capacity (U/mL)	Hydrolysable	13	1.27	0.70	0.07	−0.10	2.64	17.85	80.03	4.72	6.10	<0.001	0.06	0.06
Superoxide dismutase (U/mL)	Hydrolysable	26	−0.10	0.48	0.84	−1.04	0.84	10,035.25	84.16	4.03	4.03	<0.001	0.46	0.84
Superoxide dismutase (U/mL)	Condensed	55	0.92	0.28	<0.001	0.37	1.47	193.43	79.24	2.97	3.56	<0.001	<0.001	<0.001
Catalase activity (kU/L)	Hydrolysable	23	1.24	0.44	<0.001	0.38	2.10	34.37	81.14	2.99	3.75	<0.001	<0.001	0.01
Catalase activity (kU/L)	Condensed	51	0.94	0.21	<0.001	0.53	1.35	172.52	67.31	1.37	1.88	<0.001	<0.001	<0.001
Malondialdehyde (U/mL)	Condensed	40	−0.08	0.20	0.69	−0.47	0.31	23,640.18	55.88	0.86	0.86	<0.001	0.54	0.13
Malondialdehyde (U/mL)	Hydrolysable	13	0.99	0.76	0.19	−0.49	2.48	37.47	86.81	3.98	5.58	<0.001	0.01	<0.001
Lysozyme activity (U/mL)	Condensed	36	1.27	0.37	<0.001	0.55	2.00	49.28	82.25	3.84	3.84	<0.001	0.15	<0.001
Amylase (U/mg protein)	Condensed	65	−1.09	0.29	<0.001	−1.65	−0.53	146.37	79.57	3.66	3.76	<0.001	<0.001	0.01
Amylase (U/mg protein)	Hydrolysable	25	−0.13	0.35	0.71	−0.82	0.56	5741.09	74.05	2.10	2.10	<0.001	0.01	<0.001
Lipase (U/mg protein)	Condensed	58	−0.86	0.33	0.01	−1.50	−0.22	241.73	81.73	4.45	5.55	<0.001	<0.001	0.48
Lipase (U/mg protein)	Hydrolysable	25	−0.23	0.32	0.46	−0.86	0.39	1724.62	71.23	1.75	1.75	<0.001	0.03	0.22
Trypsin (U/mg protein)	Condensed	17	−0.91	0.56	0.10	−2.00	0.19	62.44	84.03	4.08	4.08	<0.001	0.03	<0.001
Trypsin (U/mg protein)	Hydrolysable	14	−0.13	0.79	0.87	−1.67	1.42	3351.76	86.33	7.15	7.15	<0.001	0.91	0.07
Protease (U/mg protein)	Condensed	45	−1.20	0.35	<0.001	−1.88	−0.52	75.28	77.98	3.74	4.19	<0.001	<0.001	<0.001

*Note*: Only key outcomes are presented in the main table. Complete subgroup analyses across all reported outcomes, including estimates based on smaller numbers of effect sizes, are provided in the Supporting Information (Table S4). *g*, Hedges’ *g*; *p*, significance value for the pooled effect size; *Q*, significance value from Cochran’s *Q* test for heterogeneity; Begg, significance value from Begg’s test; Egger, significance value from Egger’s regression test; FSN, fail state number; kU, kilounit; g, gram; *I^2^*, heterogeneity; *τ^2^*, estimated between‐study variance from the original random‐effects model; *τ^2^′*, estimated between‐study variance from the corresponding adjusted/prime random‐effects model; *k*, number of studies; L, litre; mg, milligram; mL, mililitre; *U*, unit; *Q*s, significance value from the *Q* statistic; SE, standard error from the random‐effects model; %, percentage.

Subgroup analysis by trophic classification showed that tannin supplementation affected cultured fish differently depending on the feeding guild (Table 2 for key outcomes; Table S5 for the complete subgroup analysis). Growth reduction was most evident in omnivorous and herbivorous species, while carnivorous species showed weaker or non‐significant responses for several growth endpoints. SGR was significantly reduced in omnivores (*g* = −1.57, 95% CI: −1.89 to −1.25, *p* < 0.001) and herbivores (*g* = −2.07, 95% CI: −3.08 to −1.05, *p* < 0.001), but not in carnivores (*g* = −0.47, 95% CI: −1.05 to 0.12, *p* = 0.12). FCR increased significantly in omnivores (*g* = 1.54, 95% CI: 1.23–1.86, *p* < 0.001) and herbivores (*g* = 1.45, 95% CI: 0.77–2.14, *p* < 0.001), whereas the effect was not significant in carnivores (*g* = 0.06, *p* = 0.81). Protein efficiency ratio was significantly reduced in all three groups: omnivores (*g* = −1.12, *p* < 0.001), carnivores (*g* = −1.10, *p* < 0.001) and herbivores (*g* = −1.82, *p* < 0.001). WGN declined significantly only in omnivores (*g* = −1.54, 95% CI: −1.97 to −1.11, *p* < 0.001), while carnivores (*p* = 0.18) and herbivores (*p* = 0.14) showed non‐significant reductions. Digestibility responses supported these growth patterns, with APD significantly reduced in omnivores (*g* = −1.60, *p* < 0.001) and herbivores (*g* = −2.30, *p* < 0.001), but only marginally reduced in carnivores (*g* = −1.90, *p* = 0.07). Apparent lipid digestibility was significantly reduced only in omnivores (*g* = −1.08, *p* < 0.001), while survival was not significantly affected in omnivores, carnivores, or herbivores (*p* > 0.05). Tannin supplementation also reduced feed intake across all trophic groups, including omnivores (*g* = −0.76, *p* < 0.001), carnivores (*g* = −0.82, *p* < 0.001), and herbivores (*g* = −1.35, *p* = 0.01). Physiological responses were more variable: antioxidant capacity increased in carnivores (*g* = 1.57, *p* < 0.001), but decreased in omnivores (*g* = −0.68, *p* = 0.03) and herbivores (*g* = −2.93, *p* = 0.01). Several immune indicators increased significantly, such as lysozyme activity in omnivores (*g* = 1.21, *p* = 0.02) and carnivores (*g* = 1.49, *p* < 0.001), white blood cells in omnivores (*g* = 0.88, *p* = 0.01), leukocytes in omnivores (*g* = 2.76, *p* < 0.001) and carnivores (*g* = 2.66, *p* < 0.001) and immune globulin in carnivores (*g* = 1.53, *p* < 0.001). However, many outcomes showed moderate to high heterogeneity, often with *I^2^* values above 70%, with *τ* ^2^ and *τ* ^2^′ likewise indicating variable between‐study variance across trophic groups and outcomes.

**Table 2:** Effects of dietary tannin supplementation on key outcomes in cultured fish according to trophic classification.

Parameter	Subgroup	*k*	*g*	SE	*p*	Lower	Upper	FsN (*R*)	*I* ^*2*^	*τ^2^*	*τ^2^′*	*Q*	Begg	Egger
Specific growth rate (%g/day)	Omnivore	224	−1.57	0.16	<0.001	−1.89	−1.25	125.55	81.28	4.01	5.48	<0.001	<0.001	<0.001
Specific growth rate (%g/day)	Carnivore	62	−0.47	0.30	0.12	−1.05	0.12	1034.18	80.13	3.74	4.24	<0.001	<0.001	<0.001
Specific growth rate (%g/day)	Herbivore	16	−2.07	0.52	<0.001	−3.08	−1.05	−1.62	73.40	2.55	4.21	<0.001	<0.001	<0.001
Feed conversion Ratio	Carnivore	52	0.06	0.25	0.81	−0.44	0.56	52294.31	74.69	2.27	2.44	<0.001	0.65	0.60
Feed conversion ratio	Omnivore	214	1.54	0.16	<0.001	1.23	1.86	131.07	80.22	3.67	4.93	<0.001	<0.001	<0.001
Feed conversion ratio	Herbivore	21	1.45	0.35	<0.001	0.77	2.14	17.13	63.52	1.46	2.70	<0.001	<0.001	<0.001
Protein efficiency ratio	Omnivore	199	−1.12	0.15	<0.001	−1.41	−0.84	405.22	74.31	2.80	3.55	<0.001	<0.001	<0.001
Protein efficiency ratio	Carnivore	23	−1.10	0.38	<0.001	−1.84	−0.36	49.70	73.40	2.13	3.44	<0.001	<0.001	<0.001
Protein efficiency ratio	Herbivore	12	−1.82	0.41	<0.001	−2.62	−1.02	1.95	46.02	0.88	1.87	0.04	<0.001	<0.001
ANPU (%)	Omnivore	98	−3.02	0.60	<0.001	−4.19	−1.85	−56.73	89.72	24.57	27.79	<0.001	<0.001	<0.001
Weight gain (%)	Omnivore	227	−1.54	0.22	<0.001	−1.97	−1.11	139.19	84.77	7.20	10.01	<0.001	<0.001	<0.001
Weight gain (%)	Carnivore	63	−0.33	0.25	0.18	−0.82	0.15	2129.98	76.71	2.69	2.69	<0.001	0.05	0.88
Weight gain (%)	Herbivore	13	−0.33	0.23	0.14	−0.78	0.11	435.31	12.27	0.08	0.08	0.32	<0.001	<0.001
Body crude protein content (%)	Carnivore	41	−0.32	0.22	0.14	−0.74	0.11	1520.44	64.13	1.16	1.16	<0.001	<0.001	<0.001
Body crude protein content (%)	Omnivore	177	<0.001	0.17	0.98	−0.33	0.32	42118817.40	77.98	3.33	3.33	<0.001	0.56	0.87
Body crude lipid content (%)	Carnivore	41	−0.19	0.22	0.39	−0.63	0.24	4272.49	64.79	1.21	1.21	<0.001	0.12	0.73
Body crude lipid content (%)	Omnivore	161	−1.21	0.20	<0.001	−1.59	−0.83	260.60	80.58	4.49	5.84	<0.001	<0.001	<0.001
Apparent protein digestibility (%)	Omnivore	103	−1.60	0.23	<0.001	−2.05	−1.14	51.79	77.60	3.73	5.56	<0.001	<0.001	<0.001
Apparent protein digestibility (%)	Herbivore	12	−2.30	0.52	<0.001	−3.32	−1.28	−3.29	60.87	1.85	2.60	<0.001	<0.001	<0.001
Apparent lipid digestibility (%)	Omnivore	35	−1.08	0.24	<0.001	−1.56	−0.61	79.38	48.46	2.07	3.69	<0.001	<0.001	<0.001
Survival rate (%)	Omnivore	61	−0.22	0.14	0.11	−0.50	0.05	4670.49	42.30	0.49	0.49	<0.001	<0.001	<0.001
Survival rate (%)	Carnivore	50	0.18	0.11	0.09	−0.03	0.40	5594.69	<0.001	<0.001	<0.001	0.48	0.34	0.01
Feed intake (g/fish/day)	Omnivore	45	−0.76	0.27	<0.001	−1.28	−0.23	256.30	69.81	2.10	2.10	<0.001	<0.001	<0.001
Feed intake (g/fish/day)	Carnivore	35	−0.82	0.28	<0.001	−1.37	−0.26	166.83	70.90	1.81	2.90	<0.001	<0.001	<0.001
Feed intake (g/fish/day)	Herbivore	14	−1.35	0.55	0.01	−2.43	−0.27	15.41	75.63	3.07	3.77	<0.001	<0.001	<0.001
Total antioxidant capacity (U/mL)	Carnivore	38	1.57	0.28	<0.001	1.03	2.11	21.48	69.35	1.88	2.84	<0.001	<0.001	<0.001
Total antioxidant capacity (U/mL)	Omnivore	10	−0.68	0.31	0.03	−1.28	−0.07	74.08	18.19	0.17	0.17	0.28	<0.001	<0.001
Superoxide dismutase (U/mL)	Omnivore	32	0.07	0.23	0.77	−0.39	0.53	26040.77	66.94	1.01	1.01	<0.001	0.38	0.71
Superoxide dismutase (U/mL)	Carnivore	47	1.35	0.44	<0.001	0.49	2.22	51.37	85.28	6.66	7.36	<0.001	0.01	<0.001
Catalase activity (kU/L)	Omnivore	30	0.41	0.19	0.03	0.03	0.78	661.14	51.50	0.49	0.98	<0.001	<0.001	<0.001
Catalase activity (kU/L)	Carnivore	45	1.48	0.32	<0.001	0.86	2.11	33.49	77.63	3.26	4.31	<0.001	<0.001	<0.001
Malondialdehyde (U/mL)	Carnivore	34	−0.12	0.33	0.72	−0.77	0.53	8823.79	76.23	2.45	4.26	<0.001	0.66	<0.001
Malondialdehyde (U/mL)	Omnivore	20	0.09	0.25	0.73	−0.41	0.58	10146.40	54.34	0.59	0.59	<0.001	0.08	<0.001
Lysozyme activity (U/mL)	Omnivore	14	1.21	0.51	0.02	0.22	2.20	22.80	80.84	2.85	2.85	<0.001	0.59	0.96
Lysozyme activity (U/mL)	Carnivore	26	1.49	0.47	<0.001	0.58	2.41	18.71	81.76	4.35	5.23	<0.001	<0.001	<0.001
Amylase (U/mg protein)	Carnivore	42	−0.48	0.23	0.04	−0.94	−0.02	660.32	68.11	1.49	1.49	<0.001	0.01	0.10
Amylase (U/mg protein)	Omnivore	42	−1.65	0.40	<0.001	−2.44	−0.86	17.33	81.48	4.76	5.33	<0.001	<0.001	<0.001
Amylase (U/mg protein)	Herbivore	10	1.14	1.12	0.31	−1.06	3.34	19.65	87.90	9.04	9.04	<0.001	0.47	<0.001
Lipase (U/mg protein)	Carnivore	42	0.53	0.30	0.08	−0.07	1.13	531.96	77.84	2.72	2.72	<0.001	<0.001	<0.001
Lipase (U/mg protein)	Omnivore	35	−1.82	0.39	<0.001	−2.58	−1.06	5.55	78.86	3.77	5.28	<0.001	<0.001	<0.001
Lipase (U/mg protein)	Herbivore	10	−2.00	0.52	<0.001	−3.01	−0.99	−0.37	63.43	1.60	2.11	<0.001	<0.001	<0.001
Trypsin (U/mg protein)	Carnivore	26	−1.36	0.43	<0.001	−2.20	−0.52	27.98	81.56	3.61	4.50	<0.001	<0.001	<0.001
Protease (U/mg protein)	Carnivore	13	−1.10	0.95	0.24	−2.96	0.76	28.07	84.46	7.75	7.75	<0.001	0.25	0.87
Protease (U/mg protein)	Omnivore	34	−0.71	0.34	0.04	−1.38	−0.03	228.48	76.28	2.86	4.06	<0.001	<0.001	<0.001

*Note*: Only key outcomes are presented in the main table. Complete subgroup analyses across all reported outcomes, including estimates based on smaller numbers of effect sizes, are provided in the Supporting Information (Table S5). *g*, Hedges’ *g*; *p*, significance value for the pooled effect size; *Q*, significance value from Cochran’s *Q* test for heterogeneity; Begg, significance value from Begg’s test; Egger, significance value from Egger’s regression test; FSN, fail state number; kU, kilounit; g, gram; *I^2^*, heterogeneity; *τ^2^*, estimated between‐study variance from the original random‐effects model; *τ^2^′*, estimated between‐study variance from the corresponding adjusted/prime random‐effects model; *k*, number of studies; L, litre; mg, milligram; mL, mililitre; *U*, unit; *Q*s, significance value from the *Q* statistic; SE, standard error from the random‐effects model; %, percentage.

Subgroup analysis by habitat showed that tannin supplementation produced clearer negative effects in freshwater species than in marine species, particularly for growth, feed efficiency, digestibility, and energy deposition (Table 3 for key outcomes; Table S6 for the complete subgroup analysis). In marine species, tannins significantly reduced SGR (*g* = −0.67, 95% CI: −1.22 to −0.11, *p* = 0.02), WGN (*g* = −0.58, 95% CI: −1.10 to −0.06, *p* = 0.03), CLC (*g* = −0.59, 95% CI: −1.01 to −0.16, *p* = 0.01), feed intake (*g* = −0.54, 95% CI: −1.05 to −0.03, *p* = 0.04) and intra‐peritoneal fat (*g* = −1.64, 95% CI: −2.71 to −0.58, *p* < 0.001), while effects on FCR, protein efficiency ratio, crude protein content, ash content, APD, apparent lipid digestibility and survival were not statistically significant. In contrast, freshwater species showed significant reductions in SGR (*g* = −1.50, 95% CI: −1.80 to −1.19, *p* < 0.001), protein efficiency ratio (*g* = −1.22, 95% CI: −1.49 to −0.94, *p* < 0.001), ANP (*g* = −2.92, 95% CI: −4.02 to −1.82, *p* < 0.001), WGN (*g* = −1.24, 95% CI: −1.61 to −0.86, *p* < 0.001), feed intake (*g* = −1.08, 95% CI: −1.57 to −0.59, *p* < 0.001), CLC (*g* = −1.04, 95% CI: −1.40 to −0.69, *p* < 0.001), APD (*g* = −1.68, 95% CI: −2.11 to −1.26, *p* < 0.001), apparent lipid digestibility (*g* = −1.00, 95% CI: −1.44 to −0.57, *p* < 0.001), apparent ash digestibility (*g* = −1.63, 95% CI: −2.92 to −0.35, *p* = 0.01) and apparent phosphorus digestibility (*g* = −1.81, 95% CI: −3.25 to −0.38, *p* = 0.01). Freshwater species also showed a significant increase in FCR (*g* = 1.38, 95% CI: 1.09–1.66, *p* < 0.001). SUR was not significantly affected in either marine (*p* = 0.07) or freshwater species (*p* = 0.38). Physiological responses were more variable: marine species showed significant increases in total antioxidant capacity, superoxide dismutase, catalase, TBARS, lysozyme activity, interleukin‐8 and immunoglobulin, while freshwater species showed significant increases in catalase, lysozyme activity, plasma haemolytic activity, white blood cells, monocytes, leukocytes, villi density, goblet cell number, Keap1, interleukin‐8 and interleukin‐10. However, many outcomes showed moderate to high heterogeneity, particularly for growth, digestibility, antioxidant and digestive enzyme responses, with *τ* ^2^ and *τ* ^2^′ estimates also varying across marine and freshwater sub‐groups.

**Table 3:** Effects of dietary tannin supplementation on key outcomes in cultured fish according to habitat.

Parameter	Category	Subgroup	*k*	*g*	SE	*p*	Lower	Upper	FsN (*R*)	*I* ^*2*^	*τ^2^*	*τ^2^′*	*Q*	Begg	Egger
Specific growth rate (%/day)	Habitat	Marine	37	−0.67	0.28	0.02	−1.22	−0.11	282.76	72.82	2.01	2.23	<0.001	<0.001	<0.001
Feed conversion ratio	Habitat	Marine	34	0.28	0.29	0.35	−0.30	0.85	1691.61	73.70	1.99	3.25	<0.001	0.04	<0.001
Protein efficiency ratio	Habitat	Marine	18	−0.60	0.34	0.08	−1.28	0.07	172.12	64.07	1.26	2.02	<0.001	<0.001	<0.001
Weight gain (%)	Habitat	Marine	44	−0.58	0.27	0.03	−1.10	−0.06	450.98	74.24	2.16	2.32	<0.001	<0.001	<0.001
Crude protein content (%)	Habitat	Marine	29	0.11	0.18	0.56	−0.25	0.47	9689.98	40.23	0.38	0.38	0.01	0.97	0.01
Crude lipid content (%)	Habitat	Marine	29	−0.59	0.22	0.01	−1.01	−0.16	296.31	52.09	0.65	0.65	<0.001	<0.001	<0.001
Survival rate (%)	Habitat	Marine	33	0.24	0.13	0.07	−0.02	0.50	2173.51	<0.001	<0.001	0.18	0.68	0.17	<0.001
Feed intake (g/fish/day)	Habitat	Marine	34	−0.54	0.26	0.04	−1.05	−0.03	412.32	67.44	1.46	2.10	<0.001	<0.001	<0.001
Total antioxidant capacity (U/mL)	Habitat	Marine	26	1.13	0.29	<0.001	0.56	1.71	51.89	66.93	1.46	2.03	<0.001	<0.001	0.01
Superoxide dismutase (U/mL)	Habitat	Marine	32	1.30	0.44	<0.001	0.43	2.17	40.52	83.72	4.79	5.09	<0.001	<0.001	<0.001
Catalase (kU/L)	Habitat	Marine	30	1.39	0.31	<0.001	0.78	2.01	29.39	71.63	1.98	2.98	<0.001	<0.001	<0.001
Malondialdehyde	Habitat	Marine	25	−0.26	0.22	0.23	−0.69	0.17	1350.83	49.95	0.58	0.58	<0.001	0.06	<0.001
Lysozyme activity	Habitat	Marine	21	1.30	0.54	0.02	0.24	2.37	26.47	83.85	4.89	5.74	<0.001	<0.001	<0.001
Amylase (U/mg protein)	Habitat	Marine	22	−0.54	0.30	0.07	−1.13	0.05	268.38	67.38	1.24	1.69	<0.001	0.04	0.04
Lipase (U/mg protein)	Habitat	Marine	22	−0.17	0.36	0.64	−0.88	0.54	2862.81	75.66	2.01	2.01	<0.001	0.54	0.07
Trypsin (U/mg protein)	Habitat	Marine	20	−1.28	0.52	0.01	−2.29	−0.27	26.93	84.33	4.22	4.89	<0.001	<0.001	<0.001
Specific growth rate (%/day)	Habitat	Freshwater	265	−1.50	0.16	<0.001	−1.80	−1.19	188.25	81.52	4.19	5.49	<0.001	<0.001	<0.001
Feed conversion Ratio	Habitat	Freshwater	253	1.38	0.14	<0.001	1.09	1.66	260.22	79.38	3.46	4.54	<0.001	<0.001	<0.001
Protein efficiency ratio	Habitat	Freshwater	216	−1.22	0.14	<0.001	−1.49	−0.94	343.65	74.15	2.79	3.57	<0.001	<0.001	<0.001
Apparent net protein utilisation (%)	Habitat	Freshwater	102	−2.92	0.56	<0.001	−4.02	−1.82	−55.97	89.47	22.43	25.48	<0.001	<0.001	<0.001
Weight gain (%)	Habitat	Freshwater	259	−1.24	0.19	<0.001	−1.61	−0.86	388.82	83.91	6.26	8.50	<0.001	<0.001	<0.001
Crude protein content (%)	Habitat	Freshwater	197	−0.20	0.16	0.20	−0.51	0.11	18,219.58	78.26	3.39	3.55	<0.001	0.37	0.24
Crude lipid content (%)	Habitat	Freshwater	181	−1.04	0.18	<0.001	−1.40	−0.69	455.80	80.66	4.43	5.64	<0.001	<0.001	<0.001
Apparent protein digestibility (%)	Habitat	Freshwater	115	−1.68	0.22	<0.001	−2.11	−1.26	41.34	76.82	3.60	5.35	<0.001	<0.001	<0.001
Apparent lipid digestibility (%)	Habitat	Freshwater	39	−1.00	0.22	<0.001	−1.44	−0.57	110.01	46.23	1.82	3.25	<0.001	<0.001	<0.001
Survival rate (%)	Habitat	Freshwater	87	−0.09	0.11	0.38	−0.30	0.12	37,449.32	33.26	0.33	0.33	<0.001	<0.001	<0.001
Feed intake (g/fish/day)	Habitat	Freshwater	60	−1.08	0.25	<0.001	−1.57	−0.59	138.37	72.32	2.49	3.49	<0.001	<0.001	<0.001
Total antioxidant capacity (U/mL)	Habitat	Freshwater	25	0.59	0.45	0.19	−0.29	1.47	247.44	78.39	3.68	3.68	<0.001	0.30	0.02
Superoxide dismutase (U/mL)	Habitat	Freshwater	50	0.37	0.29	0.20	−0.19	0.93	1354.36	79.37	2.65	3.37	<0.001	0.18	<0.001
Catalase (kU/L)	Habitat	Freshwater	45	0.73	0.24	<0.001	0.27	1.20	276.48	72.01	1.54	2.26	<0.001	<0.001	<0.001
Malondialdehyde	Habitat	Freshwater	29	0.51	0.38	0.18	−0.24	1.26	402.15	78.87	2.58	3.31	<0.001	<0.001	<0.001
Lysozyme activity	Habitat	Freshwater	19	1.46	0.42	<0.001	0.64	2.29	15.11	76.70	2.49	2.49	<0.001	0.37	0.05
Amylase (U/mg protein)	Habitat	Freshwater	72	−0.97	0.29	<0.001	−1.53	−0.41	224.67	80.43	4.16	4.37	<0.001	<0.001	0.38
Lipase (U/mg protein)	Habitat	Freshwater	65	−0.87	0.29	<0.001	−1.44	−0.30	265.31	80.13	4.00	4.98	<0.001	<0.001	0.29
Trypsin (U/mg protein)	Habitat	Freshwater	12	0.27	0.94	0.78	−1.57	2.11	628.45	86.87	8.63	8.63	<0.001	0.95	0.63
Protease (U/mg protein)	Habitat	Freshwater	51	−0.96	0.31	<0.001	−1.56	−0.36	161.37	78.15	3.40	3.86	<0.001	<0.001	0.03

*Note*: Only key outcomes are presented in the main table. Complete subgroup analyses across all reported outcomes, including estimates based on smaller numbers of effect sizes, are provided in the Supporting Information (Table S6). *g*, Hedges’ *g*; *p*, significance value for the pooled effect size; *Q*, significance value from Cochran’s *Q* test for heterogeneity; Begg, significance value from Begg’s test; Egger, significance value from Egger’s regression test; FSN, fail state number; kU, kilounit; g, gram, *I^2^*, heterogeneity; *τ^2^*, estimated between‐study variance from the original random‐effects model; *τ^2^′*, estimated between‐study variance from the corresponding adjusted/prime random‐effects model; *k*, number of studies; L, litre; mg, milligram; mL, mililitre; U, unit; Qs, significance value from the Q statistic; SE, standard error from the random‐effects model; %, percentage.

Species‐level subgroup analysis showed that the response to tannin supplementation was highly species‐dependent, with the clearest negative responses concentrated in several cyprinid and cichlid species (Table 4 for key outcomes; Table S7 for the complete subgroup analysis). The most consistent evidence came from *Labeo rohita*, where tannin supplementation significantly reduced SGR (*g* = −1.97, 95% CI: −2.44 to −1.50, *p* < 0.001), WGN (*g* = −1.66, 95% CI: −2.34 to −0.97, *p* < 0.001), protein efficiency ratio (*g* = −1.37, 95% CI: −1.77 to −0.97, *p* < 0.001), APD (*g* = −1.56, 95% CI: −2.00 to −1.12, *p* < 0.001), apparent lipid digestibility (*g* = − 0.76, 95% CI: −1.20 to −0.32, *p* < 0.001), apparent ash digestibility (*g* = −1.63, *p* = 0.01) and apparent phosphorus digestibility (*g* = −1.81, *p* = 0.01), while increasing FCR (*g* = 1.86, 95% CI: 1.38–2.33, *p* < 0.001) and faecal phosphorus concentration (*g* = 4.75, 95% CI: 2.34–7.17, *p* < 0.001). Significant negative growth or feed‐utilisation responses were also observed in *Oreochromis niloticus* for SGR (*g* = −0.60, *p* = 0.03), FCR (*g* = 0.55, *p* = 0.03), PER (*g* = −0.73, *p* < 0.001), WGN (*g* = −1.20, *p* = 0.01), body lipid content (*g* = −1.19, *p* < 0.001) and feed intake (*g* = −1.50, *p* = 0.03). Other species also showed marked negative responses, including *Oreochromis mossambicus* for SGR, FCR, PER and APD; *Ctenopharyngodon idella* for SGR, FCR, PER, WGN, body lipid, hepatosomatic index, viscerosomatic index and intra‐peritoneal fat; and *Tilapia rendalli* for SGR, FCR and PER. In contrast, several species showed non‐significant responses for key growth traits, including *Sparus aurata*, *Cyprinus carpio*, *Clarias gariepinus*, *Micropterus salmoides*, *Lateolabrax japonicus* and *Lates calcarifer*, although some of these estimates were based on small sample sizes. Survival was generally unaffected across most species. Several species‐specific estimates had high heterogeneity, often with *I^2^* > 70%, with substantial variation in *τ* ^2^ and *τ* ^2^′ among species and outcomes. Publication‐bias diagnostics were evaluated across outcomes and sub‐groups, with potential small‐study effects varying across comparisons (Tables 1–4; Tables S4–S7).

**Table 4:** Effects of dietary tannin supplementation on key outcomes in cultured fish according to fish species.

Parameter	Category	Subgroup	*k*	*g*	SE	*p*	Lower	Upper	FsN (*R*)	*I* ^*2*^	*τ^2^*	*τ^2^′*	*Q*	Begg	Egger
Specific growth rate (%g/day)	Species	*Labeo rohita*	128	−1.97	0.24	<0.001	−2.44	−1.50	−1.46	81.33	4.97	6.75	<0.001	<0.001	<0.001
Specific growth rate (%g/day)	Species	*Oreochromis niloticus*	33	−0.60	0.27	0.03	−1.14	−0.06	318.30	68.18	1.64	2.26	<0.001	0.04	0.66
Specific growth rate (%g/day)	Species	*Lateolabrax maculatus*	12	−1.10	0.68	0.10	−2.43	0.23	26.06	84.11	4.46	5.04	<0.001	0.04	0.03
Specific growth rate (%g/day)	Species	*Oreochromis mossambicus*	12	−2.90	0.78	<0.001	−4.42	−1.38	−6.51	78.30	4.84	6.92	<0.001	<0.001	<0.001
Specific growth rate (%g/day)	Species	*Lateolabrax japonicus*	12	0.22	0.21	0.31	−0.20	0.63	970.46	<0.001	<0.001	<0.001	0.64	0.09	0.96
Feed conversion ratio	Species	*Labeo rohita*	128	1.86	0.24	<0.001	1.38	2.33	14.74	81.94	5.19	7.11	<0.001	<0.001	<0.001
Feed conversion ratio	Species	*Oreochromis niloticus*	24	0.55	0.25	0.03	0.06	1.05	275.60	43.06	0.62	1.33	0.01	<0.001	<0.001
Feed conversion ratio	Species	*Lateolabrax maculatus*	14	0.54	0.46	0.25	−0.37	1.45	172.54	76.02	2.15	2.15	<0.001	0.05	0.05
Feed conversion ratio	Species	*Oreochromis mossambicus*	12	2.28	0.66	<0.001	0.99	3.57	−3.11	74.10	3.36	3.87	<0.001	<0.001	<0.001
Feed conversion ratio	Species	*Micropterus salmoides*	13	−0.11	0.34	0.76	−0.78	0.57	4474.52	52.98	0.81	0.81	0.01	0.77	0.92
Protein efficiency ratio	Species	*Labeo rohita*	124	−1.37	0.21	<0.001	−1.77	−0.97	129.48	78.00	3.62	4.60	<0.001	<0.001	<0.001
Protein efficiency ratio	Species	*Oreochromis niloticus*	25	−0.73	0.25	<0.001	−1.22	−0.24	155.72	46.98	0.70	1.21	0.01	0.15	<0.001
Weight gain (%)	Species	*Labeo rohita*	129	−1.66	0.35	<0.001	−2.34	−0.97	51.64	86.88	10.35	14.71	<0.001	<0.001	<0.001
Weight gain (%)	Species	*Oreochromis niloticus*	33	−1.20	0.44	0.01	−2.07	−0.32	55.64	81.07	4.30	6.06	<0.001	<0.001	<0.001
Weight gain (%)	Species	*Lateolabrax maculatus*	14	−0.80	0.63	0.20	−2.03	0.43	70.80	84.13	4.43	4.43	<0.001	0.10	0.90
Weight gain (%)	Species	*Micropterus salmoides*	13	0.37	0.50	0.46	−0.61	1.34	358.37	72.36	2.17	2.96	<0.001	0.86	0.01
Weight gain (%)	Species	*Ctenopharyngodon idella*	13	−1.60	0.53	<0.001	−2.63	−0.56	6.59	73.36	2.28	3.79	<0.001	<0.001	<0.001
Weight gain (%)	Species	*Lateolabrax japonicus*	12	0.27	0.21	0.21	−0.15	0.68	631.75	<0.001	<0.001	<0.001	0.66	0.09	0.08
Whole body crude protein content (%)	Species	*Labeo rohita*	124	−0.03	0.19	0.88	−0.41	0.35	549,775.80	78.02	3.23	3.31	<0.001	0.23	<0.001
Whole body crude protein content (%)	Species	*Oreochromis niloticus*	21	−0.13	0.22	0.57	−0.56	0.31	4975.64	30.39	0.31	0.53	0.09	<0.001	<0.001
Whole body crude protein content (%)	Species	*Micropterus salmoides*	13	−0.12	0.44	0.78	−0.98	0.74	3351.33	68.06	1.66	1.66	<0.001	0.44	0.49
Whole body crude lipid content (%)	Species	*Labeo rohita*	111	−0.82	0.25	<0.001	−1.31	−0.32	530.71	83.08	5.41	7.00	<0.001	<0.001	0.64
Whole body crude lipid content (%)	Species	*Oreochromis niloticus*	18	−1.19	0.36	<0.001	−1.89	−0.49	30.77	59.95	1.28	1.79	<0.001	0.01	<0.001
Whole body crude lipid content (%)	Species	*Micropterus salmoides*	13	−0.13	0.86	0.88	−1.82	1.56	2966.41	85.93	7.85	7.85	<0.001	0.44	0.94
Apparent protein digestibility (%)	Species	*Labeo rohita*	95	−1.56	0.22	<0.001	−2.00	−1.12	54.93	75.16	3.12	4.77	<0.001	<0.001	<0.001
Apparent lipid digestibility (%)	Species	*Labeo rohita*	31	−0.76	0.22	<0.001	−1.20	−0.32	174.42	40.00	0.59	1.33	0.01	<0.001	<0.001
Survival rate (%)	Species	*Oreochromis niloticus*	11	0.21	0.23	0.36	−0.24	0.65	988.36	<0.001	<0.001	<0.001	0.67	1.00	0.49
Survival rate (%)	Species	*Lateolabrax maculatus*	14	0.13	0.19	0.48	−0.24	0.51	2992.20	<0.001	<0.001	<0.001	0.98	<0.001	0.30
Survival rate (%)	Species	*Ctenopharyngodon idella*	13	−0.23	0.22	0.29	−0.67	0.20	904.11	3.74	0.02	0.25	0.41	0.01	<0.001
Survival rate (%)	Species	*Lateolabrax japonicus*	12	0.50	0.21	0.02	0.08	0.92	173.37	<0.001	<0.001	<0.001	0.90	<0.001	0.41
Feed intake (g/fish/day)	Species	*Lateolabrax maculatus*	14	−0.76	0.49	0.12	−1.71	0.19	78.69	77.91	2.50	2.50	<0.001	0.04	0.05
Feed intake (g/fish/day)	Species	*Labeo rohita*	14	0.47	0.42	0.27	−0.36	1.30	230.98	63.10	1.48	1.48	<0.001	0.05	0.11
Feed intake (g/fish/day)	Species	*Oreochromis mossambicus*	12	−1.89	0.54	<0.001	−2.95	−0.84	0.87	67.05	2.21	3.25	<0.001	<0.001	<0.001

*Note:* Only key outcomes are presented in the main table. Complete subgroup analyses across all reported outcomes, including estimates based on smaller numbers of effect sizes, are provided in the Supporting Information (Table S7). *g*, Hedges’ *g*; *p*, significance value for the pooled effect size; *Q*, significance value from Cochran’s *Q* test for heterogeneity; Begg, significance value from Begg’s test; Egger, significance value from Egger’s regression test; FSN, fail state number; kU, kilounit; g, gram; *I^2^*, heterogeneity; *τ^2^*, estimated between‐study variance from the original random‐effects model; *τ^2^′*, estimated between‐study variance from the corresponding adjusted/prime random‐effects model; *k*, number of studies; L, litre; mg, milligram; mL, mililitre; *U*, unit; *Q*s, significance value from the *Q* statistic; SE, standard error from the random‐effects model; %, percentage.

## 4. Discussion

This meta‐analysis revealed that dietary tannin supplementation can produce both adverse and beneficial effects in cultured fish species, with outcomes depending on performance metrics, trophic classification, tannin category, habitat and species. Quadratic regression identified significant dose‐related relationships for WGN (*R* ^2^ = 4.65%, *p* = 0.0008), APD (*R* ^2^ = 6.06%, *p* = 0.0227) and ANP (*R* ^2^ = 6.10%, *p* = 0.0444). In contrast, the relationships were weaker and non‐significant for SGR (*R* ^2^ = 1.13%), FCR (*R* ^2^ = 0.99%), SUR (*R* ^2^ = 3.77%) and CLC (*R* ^2^ = 1.20%) (*p* > 0.05). No optimum tannin inclusion level was identified for a SGR or WGN within the fitted range. This likely reflects the broad biological variability represented across studies and the uneven distribution of observations across inclusion levels, highlighting the importance of considering dose together with species, diet and tannin characteristics when interpreting growth responses. These results demonstrate a measurable non‐linear dose‐related component for several key responses while also indicating that the tannin inclusion level does not fully account for the observed variation, suggesting an important contribution from broader nutritional and biological factors. Tannins differ markedly in chemical structure, molecular weight, protein‐binding affinity and capacity to complex minerals or inhibit digestive enzymes; these characteristics can influence interactions with proteins and other dietary macromolecules [14, 17], while structural variation may also modify interactions with digestive enzymes [18]. Therefore, the same nominal inclusion level may produce different physiological effects depending on tannin chemistry and botanical source, even within broad categories such as hydrolysable or condensed tannins [1, 13]. These broad categories encompass substantial chemical diversity, which may contribute to the heterogeneity observed among studies. The proportion of variation explained by the tannin inclusion level further suggests that fish‐specific factors, such as species‐level digestive capacity and feeding behaviour, may also contribute to variation in responses to tannin supplementation. For example, carnivorous, omnivorous and herbivorous species may differ in their tolerance to plant secondary metabolites, enzyme profiles, gut transit time, microbiota and ability to compensate for reduced nutrient availability [29, 30]. The presence of strong negative outliers at relatively low tannin levels further indicates that low inclusion does not necessarily guarantee safety when tannins are highly reactive, diets are protein‐limited, or the species is sensitive. Positive trends seen at certain ‘high’ levels of tannin may reflect adaptive responses or reduced tannin bioavailability during longer‐term exposure. As a result, tannin supplementation should not be recommended based on a simple ‘low vs. high dose’ approach; instead, future research examining the nutritional value of tannin‐supplemented diets for aquaculture should analyse tannin concentration, tannin category, source ingredient, protein level, amino acid balance, mineral availability and digestibility responses [1, 11, 23]. This would allow future meta‐analyses to better distinguish dose effects from chemical, dietary and species‐level influences.

The tannin‐category subgroup analysis did not point to a simple growth‐suppression effect. The stronger signal was impaired nutrient use. Declines in SGR, protein efficiency, digestibility, digestive enzyme activity and body lipid deposition indicate that tannins constrained the digestion and retention of nutrients, not just the final growth output. Where the FCR increased, this interpretation became even stronger. The reduction in APD under hydrolysable tannins and the combination of lower phosphorus digestibility with higher faecal phosphorus under condensed tannins suggest that both protein and mineral retention were compromised. These responses fit well with the established anti‐nutritional behaviour of tannins in feed systems [1, 13, 14, 23]. Survival, however, remained stable across categories, which indicates a sub‐lethal nutritional cost rather than overt toxicity. Likewise, higher CAT, SOD, lysozyme activity, leukocyte counts and related immune indicators indicate changes in antioxidant and immune‐related activity, although these responses may reflect enhanced defence, adaptive responses or compensatory mechanisms depending on the broader physiological context [15, 16]. On that basis, tannin supplementation should not be discussed as broadly beneficial without much tighter consideration of tannin chemistry, dose and the basal diet. These broad tannin categories may nevertheless encompass substantial chemical variation, including differences between gallotannins and ellagitannins within hydrolysable tannins, as well as variation in molecular weight, degree of polymerisation, galloylation and botanical source, all of which may contribute to variation in biological responses among studies.

Trophic classification clearly influenced the response to dietary tannins but not in a simple way. Omnivores showed the broadest decline, with negative effects extending beyond growth to feed use, digestibility, digestive enzyme activity and body lipid deposition. This pattern points to a general nutritional penalty rather than a narrow physiological effect. Herbivores were also adversely affected. A reduced SGR and protein efficiency, together with changes in body reserves and intestinal morphology, do not support the idea that routine exposure to plant material is enough to confer tolerance to tannin‐rich diets. More likely, the response depends on the interaction between tannin chemistry, inclusion level, diet composition and digestive capacity [29, 30]. Carnivores were somewhat less consistent in their growth response but were not unaffected. Changes in feed intake, protein efficiency, digestive enzymes and several physiological variables indicate that tannins still altered the biological performance in this group [10, 15]. Survival remained unchanged across trophic categories. That matters because it means tannins behaved mainly as sub‐lethal stressors. From a production standpoint, survival by itself is therefore a weak indicator of dietary suitability. The immune‐related responses need the same caution. Higher lysozyme, leukocyte, white blood cell or immunoglobulin values may indicate immune stimulation, although their biological significance remains context‐dependent and cannot be fully resolved in the absence of direct disease‐resistance or challenge‐test endpoints, particularly where growth and nutrient use deteriorated concurrently [15, 16]. Taken together, trophic grouping explained part of the variation, but the responses were not determined by feeding habit alone. The usefulness of tannins in aquafeeds therefore depends on the particular combination of species, diet composition and inclusion level.

The habitat‐based subgroup analysis showed a clearer production‐related cost of tannin supplementation in freshwater species than that in marine species. In the freshwater subgroup, negative effects were detected for SGR, WGN, feed intake, protein efficiency ratio and APD, together with an increase in FCR, indicating reduced feed use and nutrient retention, a pattern consistent with the known anti‐nutritional properties of tannins [1, 13, 14, 23]. In marine fish, by contrast, the significant responses were limited mainly to growth, feed intake and lipid‐related traits, while the FCR, digestibility and survival remained unchanged, suggesting a weaker or less consistent production‐level response in this subgroup [10, 15]. This difference suggests a stronger production‐level cost in freshwater species, with the habitat‐related pattern likely reflecting the combined influence of habitat, species composition, tannin source, dose range, diet formulation and other study characteristics. These factors provide an important context for interpreting the stronger responses observed in freshwater species. From a feeding perspective, these results highlight the importance of evaluating tannin inclusion in the context of species, diet formulation and production conditions, particularly where feed efficiency and nutrient retention are priority outcomes.

The species‐level subgroup analysis showed that the effects of dietary tannins were strongly species‐dependent rather than uniform across fish taxa. The clearest and most consistently supported negative response was observed in *L. rohita*, which was represented by 27 studies and was one of the most extensively represented species in the dataset. This greater representation provides a stronger evidence base for estimating its response, while differences in the number of studies and effect sizes among species should be considered when comparing the strength of species‐specific patterns. In *L. rohita*, changes were seen in many factors related to feeding and digestion, such as growth rate, FCR, protein utilisation ratio, apparent protein and lipid digestibility, lipid content of the body and mineral utilisation. These data collectively suggest impaired acquisition and retention of nutrients. These findings are consistent with the known anti‐nutritional properties of tannins, including binding of proteins and other nutrients, inhibition of digestive enzymes, reduced mineral availability and enhanced excretion of nutrients via faecal material [11, 13, 14, 31]. Negative responses similar to those seen in *L. rohita* have been documented in *O. niloticus*, *O. mossambicus*, *C. idella* and *Tilapia rendalli*, suggesting that many omnivorous and herbivorous fish species may be at risk if the dietary concentration of tannins is above their ability to adaptively adjust their digestive function or utilise microorganisms within their gastrointestinal tracts to degrade them [11, 16, 31–33]. In contrast, several carnivorous and euryphagous species showed no significant adverse responses for some key growth outcomes. It is essential to recognise that the existence of such responses does not provide an indication of tolerance. Rather, they will depend upon characteristics such as the origin of the tannin used in the experiment, levels of tannin addition to the diet, diet composition, quality of dietary proteins, anatomy or physiology of the gut, length of time that fish are fed the experimental diets and the number of species‐specific databases generated from previous studies [10, 15, 30]. Another important aspect is the lack of a consistent survival response among species. This suggests that tannins are exerted primarily as sub‐lethal nutritional stresses rather than as direct toxic agents. Therefore, from a nutritional and production perspective, surviving fish may exhibit reduced feed efficiency and less efficient nutrient utilisation and storage, resulting in reduced production potential [10, 11, 15, 16]. Based on these results, it cannot be recommended that tannin supplementation should be considered a generally applicable practice across all fish species. However, consideration should be given to evaluating its utility in aquaculture feeds based on the fish species being studied and considering the biochemical properties of the tannins added to the diet along with the nutritional bioavailability of the diet components [1, 11, 13, 23].

In addition to these biological and dietary considerations, the pre‐dominance of juvenile/fingerling fish and the broad range of initial body sizes across species in the present study provide important context for interpreting the findings and may partly explain variation among studies. Considering the uneven representation of life stages and the species‐specific nature of body size, these variables were retained as descriptive study characteristics rather than being included as formal moderators. Beyond these study‐level characteristics, several methodological considerations are also relevant to the interpretation of the pooled estimates. The shared‐control adjustment used in this study reduced the influence of repeated use of common control groups, although some within‐study correlation may remain among effect sizes derived from the same study. Where appropriate, multi‐level or robust‐variance approaches provide an alternative means of accounting for such dependence. In addition, the publication‐bias diagnostics provided additional context for evaluating the robustness of the pooled estimates, with potential small‐study effects varying across outcomes and sub‐groups. A similar use of Egger’s regression and FSNs to evaluate potential publication bias has been reported in recent animal‐nutrition meta‐analyses [34]. The publication‐bias findings of the present study were considered alongside between‐study heterogeneity while recognising that publication patterns may influence the representation of positive, neutral, and negative feed‐additive responses in the available literature.

Restricting the analysis to studies using fish meal‐containing control diets provided a more consistent nutritional baseline and improved comparability among studies. This criterion also defines the scope of the inference of the present findings, which are most directly applicable to diets containing fish meal. Further evaluation in predominantly plant‐protein‐based aquafeeds would help determine how differences in the dietary matrix influence tannin responses.

## 5. Conclusion

Tannins in the diet of cultured fish should be interpreted as context‐sensitive dietary components rather than a universally beneficial feed additive. Across the analysed studies, tannin inclusion generally decreased growth and nutrient utilisation, as evidenced by lower SGR, WGN, protein efficiency ratio, nutrient digestibility, digestive enzyme activity and lipid deposition, together with higher FCR. These effects were most prevalent in studies using freshwater fish species and in the groups comprising omnivorous and herbivorous fish. Conversely, carnivorous species were less consistent at demonstrating growth reductions although they still expressed changes in feed intake, protein digestibility and utilisation, digestive enzymes, blood parameters and immune‐related responses. Although tannins increased antioxidant and immune response markers in some of the sub‐groups considered, these responses sometimes occurred alongside reduced digestibility and production performance and may represent beneficial, adaptive or compensatory physiological responses depending on the broader biological context. Survival was not adversely impacted in general, suggesting that tannins acted for the most part as sub‐lethal nutritional stressors and not as acute toxicants. The tannin inclusion level explained only a modest proportion of variation in several responses, supporting the conclusion that generic ‘lower’ versus ‘higher’ dose‐type recommendations should not be adopted for practical use. For future application in aquafeeds, we recommend that before tannins are promoted as functional feed additives for cultured fish, several factors should be carefully considered, including species‐and diet‐specific responses, tannin category, tannin source and concentration, protein‐binding capacity, basal diet composition, feeding guild and habitat of the species, digestibility responses and longer‐term effects on production performance and fish health.

## Author Contributions

**Vitas Atmadi Prakoso**: conceptualisation, methodology, investigation, data curation, formal analysis, visualisation and illustration, writing – original draft. **Danung Nur Adli and Mohammad Miftakhus Sholikin**: data curation, investigation, methodology, formal analysis, software, visualisation and illustration, writing – original draft, writing – review and editing. **Evellin Dewi Lusiana**: data curation, investigation, methodology, formal analysis, visualisation and illustration, writing – original draft. **Rendy Ginanjar**, **Dita Ariyanti, Hafizuddin Hafizuddin, Rasidi Rasidi, Tuty Maria Wardiny, Aliati Iswantari and Kurniawan Kurniawan**: data curation, investigation, methodology. **Wira Wisnu Wardani**: data curation, investigation, methodology, writing – review and editing. **Frendy Ahmad Afandi**: writing – review and editing. **Muhammad Ismunandar Yasin Saleh**: investigation, methodology. **Katherine E. Doyle**: writing – review and editing. **Arif Wibowo and Anuraga Jayanegara**: data curation, investigation, writing – review and editing.

## Funding

The authors declare that no funds, grants or other support were received during the preparation of this manuscript. Open access publishing facilitated by Charles Sturt University, as part of the Wiley ‐ Charles Sturt University agreement via the Council of Australasian University Librarians.

## Ethics Statement

This research is based on a literature review that adheres to the standards of scientific writing and accurately cites all the sources referenced in the text.

## Conflicts of Interest

The authors declare no conflicts of interest.

## Supporting Information

Additional supporting information can be found online in the Supporting Information section.

## Supporting information


**Supporting Information** The Supporting Information includes Tables S1–S7, providing details of the study selection process, characteristics of the included studies, meta‐analysis equations and statistical variables and complete subgroup analyses according to tannin category, trophic classification, habitat and fish species.

## Data Availability

The datasets employed, used and/or analysed during the present study are accessible from the corresponding author upon reasonable request.
