# Supplementary material for: Dietary Tannins in Cultured Fish: Growth Promoter or Metabolic Burden? A Meta‐Analysis
Source: Aquac Nutr. 2026 Nov 15;2026:2460161. doi: 10.1155/anu/2460161 (PMC13770322; doi:10.1155/anu/2460161)
Supplement: Supplementary file 1 [file ANU-2026-2460161-s001.zip › Supplementary Materials/Table S2.docx]

**Table S2.** Studies included in the meta-analysis of dietary tannin on cultured fish growth and health

| Study ID | Reference | Species | Trophic classification | Habitat | Initial weight (g) | Life-stage | Source of tannin | Tannin category | Level of tannin inclusion in diet (%) |
| --- | --- | --- | --- | --- | --- | --- | --- | --- | --- |
| 1 | Roy et al., 2013 | *Labeo rohita* | Omnivore | Freshwater | 3.19 ± 0.09 | Juvenile/fingerling | Sesame oil seed meal | Condensed | 0.21-0.39 |
| 2 | Roy et al., 2016 | *Labeo rohita* | Omnivore | Freshwater | 1.85 ± 0.53 | Juvenile/fingerling | Sesame diet | Condensed | 0.07-0.29 |
| 3 | Safari et al., 2020 | *Huso huso* | Carnivore | Freshwater | 39.20 ± 0.63 | Juvenile/fingerling | Commercial product, botanical source: chestnut wood | Hydrolysable | 0.05-0.1 |
| 4 | Saha & Ray, 2011 | *Labeo rohita* | Omnivore | Freshwater | 4.0 ± 0.14 | Juvenile/fingerling | Raw *Eichhornia* leaf meal | Condensed | 0.1-0.3 |
| 5 | Vo et al., 2020 | *Lates calcarifer* | Carnivore | Marine | 6.0 - 6.5 | Juvenile/fingerling | Peanut meat diet | Condensed | 0.27-0.69 |
| 6 | Mukhopadhyay & Ray, 1999a | *Labeo rohita* | Omnivore | Freshwater | 5.50 ± 0.19 | Juvenile/fingerling | Salseed meal | Hydrolysable | 0.13-0.57 |
| 7 | Wang et al., 2016 | *Scophthalmus maximus* | Carnivore | Marine | 5.11 ± 0.016 | Juvenile/fingerling | Soybean meal | Condensed | 0.16-0.27 |
| 8 | Ramachandran & Ray, 2007 | *Labeo rohita* | Omnivore | Freshwater | 1.81 ± 0.06 | Juvenile/fingerling | *P. mungo* seed meal | Condensed | 0.01-0.25 |
| 9 | Ramachandran & Ray, 2004 | *Labeo rohita* | Omnivore | Freshwater | 2.04 ± 0.21 | Juvenile/fingerling | *Lathyrus sativus* seed meal | Condensed | 0.05-0.52 |
| 10 | Ramachandran et al., 2005_study 1 | *Labeo rohita* | Omnivore | Freshwater | 7.6 ± 0.12 | Juvenile/fingerling | *Lathyrus sativus* seed meal | Condensed | 0.13-0.52 |
| 11 | Peng et al., 2021 | *Lateolabrax japonicus* | Carnivore | Marine | 10.1 ± 0.11 | Juvenile/fingerling | Commercial product, botanical source: not reported | Condensed | 0.1-0.4 |
| 12 | Peng et al., 2020a | *Lateolabrax japonicus* | Carnivore | Marine | 5.5 ± 0.11 | Juvenile/fingerling | Grape seed | Condensed | 0.18-0.98 |
| 13 | Peng et al., 2020b | *Lateolabrax japonicus* | Carnivore | Marine | 2.3 ± 0.01 | Juvenile/fingerling | Grape seed | Condensed | 0.1 |
| 14 | Peng et al., 2020c | *Lateolabrax japonicus* | Carnivore | Marine | 10.1 ± 0.11 | Juvenile/fingerling | Grape seed | Condensed | 0.98-3.95 |
| 15 | Omnes et al., 2017 | *Dicentrarchus labrax* | Carnivore | Marine | 10.2 ± 0.7 | Juvenile/fingerling | Commercial product, botanical source: Chinese gallnut grass | Hydrolysable | 1-3 |
| 16 | Okomoda et al., 2017 | *Clarias gariepinus* | Omnivore | Freshwater | 2.02 ± 0.08 | Juvenile/fingerling | *Mucuna utilis* | Condensed | 0.145-17.4 |
| 17 | Mukhopadhyay & Ray, 1999b | *Labeo rohita* | Omnivore | Freshwater | 4.75 ± 0.3 | Juvenile/fingerling | De-oiled copra meal | Condensed | 0.16-0.85 |
| 18 | Mukhopadhyay & Ray, 1999c | *Labeo rohita* | Omnivore | Freshwater | 3.82 ± 0.05 | Juvenile/fingerling | Sesame seed meal | Condensed | 0.3-0.6 |
| 19 | Mukhopadhyay & Ray, 1999d | *Labeo rohita* | Omnivore | Freshwater | 1.50 ± 0.01 | Juvenile/fingerling | Sesame seed meal (Raw) | Condensed | 0.2-0.9 |
| 20 | Mukhopadhyay & Ray, 1996 | *Labeo rohita* | Omnivore | Freshwater | 3.75 ± 0.25 | Juvenile/fingerling | De-oiled salseed meal | Hydrolysable | 0.3-2.0 |
| 21 | Hlophe and Moyo, 2014a | *Tilapia rendalli* | Herbivore | Freshwater | 11.5 ± 1 | Juvenile/fingerling | *Pennisetum clandestinum* and *Moringa oleifera* | Condensed | 4.05-16.20 and 4.21-16.8 |
| 22 | Mohammadi et al 2020 | *Oreochromis niloticus* | Omnivore | Freshwater | 3.5 ± 0.1 | Juvenile/fingerling | Canola meal | Condensed | 1.125-4.5 |
| 23 | Mbokane and Moyo 2018 | *Oreochromis mossambicus* | Omnivore | Freshwater | 33.46 ± 1.57 | Not specified/unclear | *Moringa oleifera* | Condensed | 1.3-1.6 |
| 24 | Mandal and Ghosh 2018 | *Labeo rohita* | Omnivore | Freshwater | 3.39 ± 0.06 | Juvenile/fingerling | *Pistia* leaf meal | Condensed | 0.03-0.92 |
| 25 | Liu et al 2021_study 1 | *Ctenopharyngodon idella* | Herbivore | Freshwater | ± 30 g and ± 11 g | Juvenile/fingerling | Commercial product, botanical source: not reported | Hydrolysable | 1-16 |
| 26 | Liu et al 2021_study2 | *Ctenopharyngodon idella* | Herbivore | Freshwater | ± 11 g | Juvenile/fingerling | Commercial product, botanical source: not reported | Hydrolysable | 4 |
| 27 | Bairagi et al. 2002 | *Labeo rohita* | Omnivore | Freshwater | 6.4 ± 0.31 | Juvenile/fingerling | Duckweed leaf meal | Condensed | 0.1-0.4 |
| 28 | Bairagi et al. 2004 | *Labeo rohita* | Omnivore | Freshwater | 7.15 ± 0.35 | Juvenile/fingerling | Raw *Leucana* leaf meal | Condensed | 0.9-1.8 |
| 29 | Buyukcapar et al. 2011_study 1 | *Oreochromis niloticus* | Omnivore | Freshwater | 16.18 ± 0.03 | Juvenile/fingerling | Commercial product, botanical source: not reported | Condensed | 0.5-2.5 |
| 30 | Buyukcapar et al. 2011_study 2 | *Oreochromis niloticus* | Omnivore | Freshwater | 16.18 ± 0.03 | Juvenile/fingerling | Commercial product, botanical source: not reported | Hydrolysable | 0.5-2.5 |
| 31 | Ghosh and Mandal 2015 | *Labeo rohita* | Omnivore | Freshwater | 3.24 ± 0.11 | Juvenile/fingerling | Diets with raw groundnut oil cake | Condensed | 0.04-1.45 |
| 32 | Fawole et al., 2017 | *Labeo rohita* | Omnivore | Freshwater | 3.25 ± 0.02 | Juvenile/fingerling | Defatted Jatropha seed meal | Condensed | 0.9135-3.65 |
| 33 | Dongmeza et al., 2006 | *Oreochromis niloticus* | Omnivore | Freshwater | 2.5–3.9 | Juvenile/fingerling | moringa leaf meal | Condensed | 0.1-0.6 |
| 34 | Collins et al., 2013 | *Oncorhynchus mykiss* | Carnivore | Freshwater | 235.2 | Grow-out | Commercial product, botanical source: Soybean meal, pea meal, canola meal, soy protein concentrate, pea protein concentrate, canola protein concentrate, and non-starch polysaccharide | Condensed | 1.5-3.2 |
| 35 | Fadel et al., 2021 | *Oreochromis* sp. | Omnivore | Freshwater | 85.5 ± 5 | Juvenile/fingerling | Carob seed germ meal | Condensed | 0.04-0.103 |
| 36 | Garg et al., 2002 | *Cirrhinus mrigala* | Omnivore | Freshwater | 6.15 ± 0.14 | Juvenile/fingerling | Soybean, moong, cowpea, and guar meal | Condensed | 0.002011-0.004165 |
| 37 | Ramachandran and Ray, 2008 | *Labeo rohita* | Omnivore | Freshwater | 3.18 ± 0.11 | Juvenile/fingerling | *Lathyrus* seed meal | Condensed | 0.25-1.3 |
| 38 | Helmiati et al. 2020 | *Oreochromis* sp. | Omnivore | Freshwater | 13.87-14.71 | Juvenile/fingerling | Fermented Moringa leaves meal | Condensed | 0.139-0.417 |
| 39 | Hlophe and Moyo, 2014b | *Oreochromis mossambicus* | Omnivore | Freshwater | 12.5 | Juvenile/fingerling | Kikuyu leaf meals and Moringa leaf meals | Condensed | 0.63-2.52 and 0.879-3.51 |
| 40 | Hossain et al., 2001 | *Cyprinus carpio* |  | Freshwater | 5.25 ± 0.03 | Juvenile/fingerling | *Sesbania aculeata* seed meal | Condensed | 0.27-1.08 |
| 41 | Hossain et al., 2002 | *Oreochromis niloticus* | Omnivore | Freshwater | 7.06 ± 0.03 | Juvenile/fingerling | *Sesbania aculeata* seed meal | Condensed | 0.22-0.87 |
| 42 | Iheanacho et al., 2018 | *Clarias gariepinus* | Omnivore | Freshwater | 75.07 ± 2.97 | Juvenile/fingerling | Ginger (*Zingiber officinale*) | Condensed | 0.0015-0.006 |
| 43 | Mzengereza et al., 2021 | *Pagrus major* | Carnivore | Marine | 6.5 | Juvenile/fingerling | Camelina meal | Condensed | 0.7-1.126 |
| 44 | Amri et al., 2022 | *Sparus aurata* | Omnivore | Marine | - | Not specified/unclear | *Aloe vera* | Hydrolysable | 0.5-5 |
| 45 | Chen et al. 2022 | *Lateolabrax maculatus* | Carnivore | Marine | 2.7 ± 0.02 | Juvenile/fingerling | Commercial product, botanical source: Grape seed | Condensed | 0.1-0.2 |
| 46 | Das et al. 2022 | *Labeo rohita* | Omnivore | Freshwater | 2.5-3.5 | Juvenile/fingerling | fermented mahua oil cake | Condensed | 0.217-1.085 |
| 47 | Peng et al. 2022a | *Lateolabrax maculatus* | Carnivore | Marine | 2.90 ± 0.02 | Juvenile/fingerling | Commercial product, botanical source: Kyoho grape (*Vitis vinifera* L.) seed | Condensed | 0.1 |
| 48 | Peng et al. 2022b | *Lateolabrax maculatus* | Carnivore | Marine | 2.75 ± 0.03 | Juvenile/fingerling | Commercial product, botanical source: Grape seed | Condensed | 0.1-0.2 |
| 49 | Peng et al. 2022c | *Lateolabrax maculatus* | Carnivore | Marine | 2.7 ± 0.03 | Juvenile/fingerling | Commercial product, botanical source: Grape seed | Condensed | 0.1-0.2 |
| 50 | Peng et al. 2022d | *Lateolabrax maculatus* | Carnivore | Marine | 2.9 ± 0.1 | Juvenile/fingerling | Commercial product, botanical source: Grape seed | Condensed | 0.1 |
| 51 | Imperatore et al. 2023 | *Danio rerio* | Omnivore | Freshwater | 0.310 ± 0.118 | Adult/broodstock | Commercial product, botanical source: chestnut and quebracho wood | Mixed (Condensed + Hydrolysable) | 0.09-0.34 |
| 52 | Olude and Akinduti 2023 | *Oreochromis niloticus* | Omnivore | Freshwater | 6.21 ± 0.05 | Juvenile/fingerling | Fermented Cassava leaf meal | Condensed | 0.0656-0.1968 |
| 53 | Qiu et al. 2023 | *Lateolabrax maculatus* | Carnivore | Marine | 10.9 | Juvenile/fingerling | Commercial product, botanical source: *Rhus chinensis* | Hydrolysable | 0.1-0.4 |
| 54 | Cuevas-Rodríguez et al. 2024 | *Oreochromis niloticus* | Omnivore | Freshwater | 1.8 ± 0.2 | Juvenile/fingerling | Jackfruit (*Artocarpus heterophyllus*) seed meal | Condensed | 0.0129- 0.0653 |
| 55 | Feng et al. 2024 | *Cyprinus carpio* | Omnivore | Freshwater | 32.16 ± 1.40 | Juvenile/fingerling | Commercial product, botanical source: gallnut | Hydrolysable | 0.5-2 |
| 56 | Khumujam et al. 2024 | *Labeo rohita* | Omnivore | Freshwater | 5.28 ± 0.34 | Juvenile/fingerling | Commercial product, botanical source: not reported | Hydrolysable | 1.25 |
| 57 | Oghenochuko et al. 2024 | *Clarias gariepinus* | Omnivore | Freshwater | 28.0 ± 1.24 | Juvenile/fingerling | Onion bulb | Not specified | 0.02375-0.19 |
| 58 | Qiu et al. 2024a_study 1 | *Lateolabrax maculatus* | Carnivore | Marine | 10.9 ± 0.02 | Juvenile/fingerling | Commercial product, botanical source: not reported | Hydrolysable | 0.2 |
| 59 | Qiu et al. 2024a_study 2 | *Lateolabrax maculatus* | Carnivore | Marine | 10.9 ± 0.02 | Juvenile/fingerling | Commercial product, botanical source: not reported | Condensed | 0.2 |
| 60 | Qiu et al. 2024a_study 3 | *Lateolabrax maculatus* | Carnivore | Marine | 10.9 ± 0.02 | Juvenile/fingerling | hydrolysable tannin + condensed tannin | Mixed (Condensed + Hydrolysable) | 0.2 |
| 61 | Qiu et al. 2024b | *Lateolabrax maculatus* | Carnivore | Marine | 10.9 | Juvenile/fingerling | Commercial product, botanical source: Kyoho grape seed | Condensed | 0.1 |
| 62 | Raman et al. 2024_study 1 | *Labeo rohita* | Omnivore | Freshwater | 4.43 ± 0.12 | Juvenile/fingerling | Sesbania leaf meal | Condensed | 0.2-0.8 |
| 63 | Raman et al. 2024_study 2 | *Labeo rohita* | Omnivore | Freshwater | 4.43 ± 0.12 | Juvenile/fingerling | Fermented Sesbania leaf meal | Condensed | 0.08-0.32 |
| 64 | Brutti et al. 2024 | *Oreochromis niloticus* | Omnivore | Freshwater | 1.8 ± 0.02 | Juvenile/fingerling | Black wattle | Condensed | 0.015-0.1 |
| 65 | Yang et al. 2024 | *Micropterus salmoides* | Carnivore | Marine | 5.99 ± 0.03 | Juvenile/fingerling | Bark of quebracho | Condensed | 0.375 |
| 66 | Yang et al. 2025 | *Micropterus salmoides* | Carnivore | Marine | 5.99 ± 0.03 | Juvenile/fingerling | Quebracho | Condensed | 0.06-0.938 |
| 67 | Marchão et al. 2025a | *Megaleporinus obtusidens* | Omnivore | Freshwater | 36.5 ± 1.8 | Juvenile/fingerling | Mango (*Mangifera indica*) meal | Condensed | 0.045-0.123 |
| 68 | Hidayat et al. 2025_study 1 | *Tilapia* sp. | Omnivore | Freshwater | 8.19 ± 0.01 g | Juvenile/fingerling | non pretreated Jack Bean *Canavalia ensiformis* | Condensed | 0.19-0.57 |
| 69 | Hidayat et al. 2025_study 2 | *Tilapia* sp. | Omnivore | Freshwater | 8.19 ± 0.01 g | Juvenile/fingerling | Pretreated Jack Bean *Canavalia ensiformis* | Condensed | 0.1085 - 0.3255 |
| 70 | Orisasona et al. 2025_study 1 | *Heterobranchus longifilis* | Omnivore | Freshwater | 3.82±0.05 g | Juvenile/fingerling | Almond *Terminalia catappa* leaf ethanoic extracts | Hydrolysable | 0.00293 – 0.00586 |
| 71 | Orisasona et al. 2025_study 2 | *Heterobranchus longifilis* | Omnivore | Freshwater | 3.82±0.05 g | Juvenile/fingerling | Almond *Terminalia catappa* leaf aqueous extracts | Hydrolysable | 0.00105 – 0.0021 |
| 72 | Gonzaga-Torino et al. 2025 | *Tilapia* sp. | Omnivore | Freshwater | 1.2 g | Juvenile/fingerling | Fermented calamansi (*Citrus microcarpa*) waste | Not specified | 0.04701 |
| 73 | Libanori et al. 2025 | *Oreochromis niloticus* | Omnivore | Freshwater | 13.13 ±1.71 g | Juvenile/fingerling | Black wattle bark (*Acacia mearnsii*) | Condensed | 0.2-0.8 |
| 74 | Ponsin et al. 2025 | *Betta splendens* | Carnivore | Freshwater | 0.42 ± 0.02 g | Not specified/unclear | Karonda (*Carissa carandas*) | Condensed | 0.01229-0.03687 |
| 75 | Marchão et al. 2025b | *Megaleporinus obtusidens* | Omnivore | Freshwater | 27.26 ± 0.48g | Juvenile/fingerling | Whole guava meal | Not specified | 0.045 – 0.123 |
| 76 | Wang et al. 2025 | *Micropterus salmoides* | Carnivore | Freshwater | 26.15±3.59 g | Juvenile/fingerling | Commercial product, botanical source: not reported | Hydrolysable | 0.1 – 1.2 |
| 77 | Angom et al. 2025 | *Ompok bimaculatus* | Carnivore | Freshwater | 1.24±0.23 g | Juvenile/fingerling | Winged bean meal | Condensed | 0.01056 - 0.07038 |
| 78 | Dong et al. 2025 | *Lateolabrax maculatus* | Carnivore | Marine | ~500 g | Juvenile/fingerling | Grape seed | Condensed | 0.1 |
| 79 | Kang et al. 2025 | *Micropterus salmoides* | Carnivore | Freshwater | 8.17 ± 0.04 g | Juvenile/fingerling | Commercial product, botanical source: not reported | Condensed | 0.062 – 0.31 |
| 80 | Xue et al. 2024 | *Mastacembelus armatus* | Carnivore | Freshwater | 0.40 ± 0.005 g | Juvenile/fingerling | Commercial product, botanical source: not reported | Hydrolysable | 0.05 – 0.2 |
| 81 | Kang et al. 2023 | *Micropterus salmoides* | Carnivore | Freshwater | 8.17 ± 0.04 g | Juvenile/fingerling | Grape seed | Condensed | 0.062 – 0.31 |
| 82 | Yao et al. 2022 | *Ctenopharyngodon idella* | Herbivore | Freshwater | 8.18 ± 0.81 g | Juvenile/fingerling | Commercial product, botanical source: not reported | Hydrolysable | 0.75 – 1.75 |
| 83 | Peng et al. 2022e | *Lateolabrax maculatus* | Carnivore | Marine | 6.0 ± 0.15 g | Juvenile/fingerling | Commercial product, botanical source: grape seed | Condensed | 0.1 |
| 84 | Olude et al. 2021 | *Labeo rohita* | Omnivore | Freshwater | 2.03 ± 0.01 g | Juvenile/fingerling | Cassava leaf meal | Condensed | 0.002 – 0.005 |
| 85 | Yao et al. 2019a | *Ctenopharyngodon idella* | Herbivore | Freshwater | 8.18 ± 0.81 g | Juvenile/fingerling | Commercial product, botanical source: not reported | Hydrolysable | 0.75 – 1.75 |
| 86 | Yao et al. 2019b | *Ctenopharyngodon idella* | Herbivore | Freshwater | 8.18 ± 0.81 g | Juvenile/fingerling | Commercial product, botanical source: not reported | Hydrolysable | 1.25 |
| 87 | Thitichayaphong and Rukkwamsuk 2016 | *Oreochromis niloticus* | Omnivore | Freshwater | juvenile (size not reported) | Juvenile/fingerling | Commercial product, botanical source: not reported | Condensed | 0.4 |
| 88 | Rabelo et al. 2016 | *Rhamdia quelen* | Omnivore | Freshwater | 4.49 ± 0.80 g | Juvenile/fingerling | Sorghum | Condensed | 0.0014 - 0.0072 |
| 89 | Meriç 2013 | *Cyprinus carpio* | Omnivore | Freshwater | 993.82±11.68 g | Grow-out | Sunflower seedmeal | Condensed | 0.22 - 0.61 |
| 90 | Prusty et al. 2007 | *Labeo rohita* | Omnivore | Freshwater | 3.2 g | Juvenile/fingerling | Commercial product, botanical source: not reported | Hydrolysable | 0.12 - 1.87 |
| 91 | Ramachandran et al. 2005_study 2 | *Labeo rohita* | Omnivore | Freshwater | 7.6 ± 0.12 g | Juvenile/fingerling | Raw *Lathyrus* seed meal | Condensed | 0.13 - 0.52 |
| 92 | Mukhopadhyay & Ray 2005_study 1 | *Labeo rohita* | Omnivore | Freshwater | 4.50 ± 0.11 g | Juvenile/fingerling | Raw linseed meal | Condensed | 0.52 – 1.12 |
| 93 | Mukhopadhyay & Ray 2005_study 2 | *Labeo rohita* | Omnivore | Freshwater | 4.50 ± 0.11 g | Juvenile/fingerling | Fermented linseed meal | Condensed | 0.3 – 0.68 |
| 94 | Quintero Pinto et al. 2001 | *Leporinus macrocephalus* | Omnivore | Freshwater | 5.06 ± 0.20 g | Juvenile/fingerling | Barbatimão (*Dimorphandra mollis*) | Condensed | 0.23 – 1.82 |
| 95 | Mukhopadhyay & Ray 2001 | *Labeo rohita* | Omnivore | Freshwater | 1.50 ± 0.3 g | Juvenile/fingerling | Fermented linseed meal | Condensed | 0.21 – 0.72 |
| 96 | Becker & Makkar 1999_study 1 | *Cyprinus carpio* | Omnivore | Freshwater | 5.0 g | Juvenile/fingerling | Commercial product, botanical source: not reported | Hydrolysable | 2 |
| 97 | Becker & Makkar 1999_study 2 | *Cyprinus carpio* | Omnivore | Freshwater | 5.0 g | Juvenile/fingerling | Quebracho | Condensed | 2 |

**References**

Amri, A., Bouraoui, Z., Balbuena-Pecino, S., Capilla, E., Gharred, T., Haouas, Z., Guerbej, H., Hosni, K., Navarro, I., & Jebali, J. (2022). Dietary supplementation with *Aloe vera* induces hepatic steatosis and oxidative stress together with a disruption of cellular signalling pathways and lipid metabolism related genes' expression in gilthead sea bream (*Sparus aurata*). *Aquaculture*, *559*, Article 738433. [https://doi.org/10.1016/j.aquaculture.2022.738433](http://?)

Angom, J., Singh, S. K., Devi, A. G., Das, P., Biswas, P., Waikhom, G., Ngasotter, S., Debbarma, R., Debbarma, S., & Yadav, N. K. (2025). Effects of substituting soybean meal with winged bean on growth, physiological function and flesh quality of Indian butter catfish (*Ompok bimaculatus*). *Aquaculture Nutrition, 2025*, 9959405. [https://doi.org/10.1155/anu/9959405](http://?)

Bairagi, A., Ghosh, K. S., Sen, S. K., & Ray, A. K. (2002). Duckweed (*Lemna polyrhiza*) leaf meal as a source of feedstuff in formulated diets for rohu (*Labeo rohita* Ham.) fingerlings after fermentation with a fish intestinal bacterium. *Bioresource Technology, 85,* 17–24.

Bairagi, A., Ghosh, K. S., Sen, S. K., & Ray, A. K. (2004). Evaluation of the nutritive value of *Leucaena leucocephala* leaf meal, inoculated with fish intestinal bacteria *Bacillus subtilis* and *Bacillus circulans* in formulated diets for rohu, *Labeo rohita* (Hamilton) fingerlings. *Aquaculture research*, *35*, 436-446.

Becker, K., & Makkar, H. P. S. (1999). Effects of dietary tannic acid and quebracho tannin on growth performance and metabolic rates of common carp (*Cyprinus carpio* L.). *Aquaculture, 175*(3–4), 327–335. [https://doi.org/10.1016/S0044-8486(99)00106-4](http://?)

Brutti, D. D., Uez, D. E., Maysonnave, G. S., Konrath, R. A., da Silva, V. M., Costa, S. Z. R., & Sutili, F. J. (2024). Condensed tannins from black wattle as a promising nutritional additive for Nile tilapia: Growth, immune and antioxidant responses, and gut morphology. *Animal Feed Science and Technology*, *318*, Article 116152. [https://doi.org/10.1016/j.anifeedsci.2024.116152](http://?)

Buyukcapar, H. M., Atalay, A. I., & Kamalak, A. (2011). Growth performance of Nile tilapia (*Oreochromis niloticus*) fed with diets containing different levels of hydrolysable and condensed tannin. *Journal of Agricultural Science and Technology*, *13*(7), 1045-1051.

Chen, B., Qiu, J., Wang, Y., Huang, W., Zhao, H., Zhu, X., & Peng, K. (2022). Condensed tannins increased intestinal permeability of Chinese seabass (*Lateolabrax maculatus*) based on microbiome-metabolomics analysis. *Aquaculture*, *560*, Article 738615. [https://doi.org/10.1016/j.aquaculture.2022.738615](http://?)

Collins, S. A., Mansfield, G. S., Desai, A. R., Van Kessel, A. G., Hill, J. E., & Drew, M. D. (2013). Structural equation modelling of antinutrients in rainbow trout diets and their impact on feed intake and growth. *Aquaculture*, *416*, 219-227. [https://doi.org/10.1016/j.aquaculture.2013.09.020](http://?)

Cuevas-Rodríguez, B. L., Zavala-Leal, O. I., Ruiz-Velazco, M., Cuevas-Rodríguez, E. O., Sánchez-Magaña, L., & Valdez-González, F. J. (2024). Effect of Jackfruit (*Artocarpus heterophyllus*) Seed Processing on the Diets of Nile Tilapia (*Oreochromis niloticus*): Growth, Antinutrients, and Blood Parameters. *Indian Journal of Animal Research, 58*, 69-78. [https://doi.org/10.18805/ijar.Bf-1717](http://?)

Das, K. C., Mohanty, S., Sahoo, P. K., Sahoo, S., Prakash, B., & Swain, P. (2022). Inclusion of different levels of solid-state fermented mahua oil cake on growth, digestibility and immunological parameters of rohu (*Labeo rohita*). *Aquaculture*, *553*, 738049. [https://doi.org/10.1016/j.aquaculture.2022.738049](http://?)

Dong, R., Qiu, J., Cao, J., Huang, W., Chen, B., Zhao, H., Sun, W., Lu, H., Loh, J.-Y., & Peng, K. (2025). *In vitro* and *in vivo* evaluation of the effects of condensed tannins and catechins monomers on antioxidant and intestinal health of Chinese seabass (*Lateolabrax maculatus*). *Frontiers in Veterinary Science, 12*, 1558942. https://doi.org/10.3389/fvets.2025.1558942

Dongmeza, E., Siddhuraju, P., Francis, G., & Becker, K. (2006). Effects of dehydrated methanol extracts of moringa (*Moringa oleifera* Lam.) leaves and three of its fractions on growth performance and feed nutrient assimilation in Nile tilapia (*Oreochromis niloticus* (L.)). *Aquaculture*, *261*(1), 407-422. [https://doi.org/10.1016/j.aquaculture.2006.08.006](http://?)

Fadel, A. H. I., Kamarudin, M. S., Romano, N., Ebrahimi, M., Saad, C. R., & Samsudin, A. A. (2021). Pretreating carob seed germ meal enhanced digestibility, growth performance and feed utilization in red tilapia (*Oreochromis* sp.). *Animal Feed Science and Technology*, *278*, 114976. [https://doi.org/10.1016/j.anifeedsci.2021.114976](http://?)

Fawole, F. J., Sahu, N. P., Shamna, N., Phulia, V., Emikpe, B. O., Adeoye, A. A., Aderolu, A. Z., & Popoola, O. M. (2018). Effects of detoxified *Jatropha curcas* protein isolate on growth performance, nutrient digestibility and physio-metabolic response of *Labeo rohita* fingerlings. *Aquaculture Nutrition*, *24*(4), 1223-1233. [https://doi.org/10.1111/anu.12660](http://?)

Feng, H., Zhao, S., Ma, X., Zhang, Y., Zhang, M., Zeng, Y., Zhao, J., Liu, Z., & Liu, H. (2024). Effects of dietary supplementation of gallic tannic acid on growth, intestinal digestive enzymes activity, innate immunity, morphology, and microbial composition of *Cyprinus carpio*. *Aquaculture International*, *32*(4), 3815-3833. [https://doi.org/10.1007/s10499-023-01352-9](http://?)

Garg, S. K., Kalla, A., & Bhatnagar, A. (2002). Evaluation of raw and hydrothermically processed leguminous seeds as supplementary feed for the growth of two Indian major carp species. *Aquaculture research*, *33*, 151-163.

Ghosh, K., & Mandal, S. (2015). Nutritional evaluation of groundnut oil cake in formulated diets for rohu, *Labeo rohita* (Hamilton) fingerlings after solid-state fermentation with a tannase producing yeast, *Pichia kudriavzevii* (GU939629) isolated from fish gut. *Aquaculture Reports*, *2*, 82-90. [https://doi.org/10.1016/j.aqrep.2015.08.006](http://?)

Gonzaga-Torino, F. M., Candole, K. P., Bajalla, M. L. B., Catienza, F. A., Anuevo, G. M., Özdemir, K. Y., Apines-Amar, M. J. S., Camarin, M.-A. M., Fagutao, F. F., Choresca, C. H., Jr., & Pedroso, F. L. (2025). Fermented calamansi (*Citrus microcarpa*) waste as a functional feed additive: Nutritional, antioxidant, and anti-nutritional insights for tilapia (*Oreocrhromis* sp.). *International Journal of Aquatic Biology, 13*(3), 19–33. [https://doi.org/10.22034/ijab.v13i3.2472](http://?)

Helmiati, S., Rustadi, Isnansetyo, A., & Zuprizal. (2020). Replacement of fish meal with fermented Moringa leaves meal and its effect on the growth performance of red tilapia (*Oreochromis* sp.). *AACL Bioflux*, *13*(3), 1622-1632.

Hidayat, A. P. N., Setiawati, M., Ekasari, J., Adestia, T. S., & Fauzi, I. A. (2025). Utilization of pretreated jack bean (*Canavalia ensiformis*) as raw material for tilapia (*Oreochromis* sp.) feed. *Egyptian Journal of Aquatic Biology & Fisheries, 29*(5), 213–234.

Hlophe, S. N., & Moyo, N. A. G. (2014a). A comparative study on the use of *Pennisetum clandestinum* and *Moringa oleifera* as protein sources in the diet of the herbivorous *Tilapia rendalli*. *Aquaculture International*, *22*(4), 1245-1262. [https://doi.org/10.1007/s10499-013-9744-4](http://?)

Hlophe, S. N., & Moyo, N. A. G. (2014b). Evaluation of kikuyu grass and moringa leaves as protein sources in *Oreochromis mossambicus* diets. *African Journal of Aquatic Science*, *39*(3), 305-312. [https://doi.org/10.2989/16085914.2014.958049](http://?)

Hossain, M. A., Focken, U., & Becker, K. (2001). Evaluation of an unconventional legume seed, *Sesbania aculeata*, as a dietary protein source for common carp, *Cyprinus carpio* L. *Aquaculture*, *198*, 129–140.

Hossain, M. A., Focken, U., & Becker, K. (2002). Nutritional evaluation of dhaincha (*Sesbania aculeata*) seeds as dietary protein source for tilapia *Oreochromis niloticus*. *Aquaculture research*, *33*, 653-662.

Iheanacho, S. C., Ogbu, M. U., Ude, F. E., Ayotunde, I. O., & Ogueji, E. O. (2018). Growth, Hematology and Immuno-Modulatory Potential of Ginger (*Zingiber officinale*) Supplemented Diets in *Clarias gariepinus* Juvenile (Burchell, 1822). *Aquaculture Studies*, *18*(1), 41-49. [https://doi.org/10.4194/2618-6381-v18_1_05](http://?)

Imperatore, R., Fronte, B., Scicchitano, D., Orso, G., Marchese, M., Mero, S., Licitra, R., Coccia, E., Candela, M., & Paolucci, M. (2023). Dietary Supplementation with a Blend of Hydrolyzable and Condensed Tannins Ameliorates Diet-Induced Intestinal Inflammation in Zebrafish (*Danio rerio*). *Animals*, *13*(1), Article 167. [https://doi.org/10.3390/ani13010167](http://?)

Kang, P., Hang, Y., Chen, C., Pan, Y., Wang, Q., & Hua, X. (2023). Effects of replacing fishmeal with rapeseed meal and dietary condensed tannins on antioxidant capacity, immunity, and hepatic and intestinal health of largemouth bass (*Micropterus salmoides*). *Aquaculture Reports, 30*, 101548. [https://doi.org/10.1016/j.aqrep.2023.101548](http://?)

Kang, P., Xia, X., Hang, Y., & Hua, X. (2025). Exploring the relationship between dietary rapeseed meal, condensed tannin and growth, nutrive metabolism in largemouth bass (*Micropterus salmoides*). *Aquaculture and Fisheries, 10*(5), 825–835. [https://doi.org/10.1016/j.aaf.2024.02.004](http://?)

Khumujam, S. D., Dasgupta, S., Srivastava, P. P., Sahu, N. P., & Varghese, T. (2024). Interactive effects of dietary saponin with cholesterol and tannin on growth and biochemical responses in *Labeo rohita* (Hamilton, 1822) fingerlings. *Aquaculture International*, *32*(4), 4141-4157. [https://doi.org/10.1007/s10499-023-01368-1](http://?)

Libanori, M. C. M., Gomes dos Santos, G., Souza, A. P., da Silva Costa, D., Saldana-Serrano, M., Berlofa Ferreira, M., de Araújo Pereira Junior, J., Bellettini, F., Pereira Dutra, S. A., Laterça Martins, M., Owatari, M. S., & Pedreira Mouriño, J. L. (2025). Anti-inflammatory, immunostimulant and antimicrobial properties of tannic acid in the diet of *Oreochromis niloticus* infected with *Aeromonas hydrophila*. *Fish and Shellfish Immunology, 157*, 110119. [https://doi.org/10.1016/j.fsi.2025.110119](http://?)

Liu, L. W., Liang, X. F., Fang, J. G., Yuan, X. C., Li, B., & Li, J. (2021). Tannase alleviates the adverse physiological and toxicological impacts of supplementing tannin in the diet of grass carp (*Ctenopharyngodon idellus*). *Aquaculture Nutrition*, *27*(5), 1612-1625. [https://doi.org/10.1111/anu.13301](http://?)

Mandal, S., & Ghosh, K. (2018). Utilization of Fermented Pistia Leaves in the Diet of Rohu, *Labeo rohita* (Hamilton): Effects on Growth, Digestibility and Whole Body Composition. *Waste and Biomass Valorization*, *10*(11), 3331-3342. [https://doi.org/10.1007/s12649-018-0336-4](http://?)

Marchão, R. S., Copatti, C. E., Rocha, A. D. S., Costa, T. S., Silva, R. C. D., Pereira, G. A., Rocha, D. R. D., & Melo, J. F. B. (2025a). Mango (*Mangifera indica*) meal can partially replace corn meal in diets for piava (*Megaleporinus obtusidens*). *Ciencia Rural*, *55*(4), Article e20230670. [https://doi.org/10.1590/0103-8478cr20230670](http://?)

Marchão, R. S., Copatti, C. E., Rocha, A. S., Silva, R. C., Santana, G. A., Pereira, G. A., Rocha, D. R., Souza, A. M., Figueiredo, R. A. C. R., & Melo, J. F. B. (2025b). Whole guava meal can partially replace corn meal in diets for piava (*Megaleporinus obtusidens*). *Arquivo Brasileiro de Medicina Veterinária e Zootecnia, 77*(6), e13546.

Mbokane, E. M., & Moyo, N. A. G. (2018). Alterations of haemato-biochemical parameters pre and postchallenge with *Aeromonas hydrophila* and survival of *Oreochromis mossambicus* fed *Moringa oleifera*-based diets. *Fish Shellfish Immunol*, *83*, 213-222. [https://doi.org/10.1016/j.fsi.2018.09.017](http://?)

Meriç, İ. (2013). Evaluation of sunflower seed meal in feeds for carp: Antinutritional effects on antioxidant defense system. *Journal of Food, Agriculture & Environment, 11*(2), 1128–1132.

Mohammadi, M., Imani, A., Farhangi, M., Gharaei, A., & Hafezieh, M. (2020). Replacement of fishmeal with processed canola meal in diets for juvenile Nile tilapia (*Oreochromis niloticus*): Growth performance, mucosal innate immunity, hepatic oxidative status, liver and intestine histology. *Aquaculture*, *518*. [https://doi.org/10.1016/j.aquaculture.2019.734824](http://?)

Mukhopadhyay, N., & Ray, A. K. (1999a). Effect of fermentation on the nutritive value of sesame seed meal in the diets for rohu, *Labeo rohita* (Hamilton), fingerlings. *Aquaculture Nutrition 5*, 229-236.

Mukhopadhyay, N., & Ray, A. K. (1996). The potential of deoiled sal (*Shorea robusta*) seed meal as a feedstuff in pelleted feed for Indian major carp, rohu, *Labeo rohita* (Hamilton), fingerlings. *Aquaculture Nutrition 2*, 221-227.

Mukhopadhyay, N., & Ray, A. K. (1999b). Improvement of quality of sal (*Shorea robusta*) seed meal protein with supplemental amino acids in feeds for rohu, *Labeo rohita* (Hamilton), fingerlings. *Acta Ichthyologica Et Piscatoria*, *XXIX*, 25-39.

Mukhopadhyay, N., & Ray, A. K. (1999c). Improvement of quality of sesame *Seasamum indicum* seed meal protein with supplemental amino acids in feeds for rohu *Labeo rohita* (Hamilton) fingerlings. *Aquaculture research*, *30*, 549-557.

Mukhopadhyay, N., & Ray, A. K. (1999d). Utilization of copra meal in the formulation of compound diets for rohu, *Labeo rohita*, fingerlings. *Journal of Applied Ichthyology 15*, 127-131.

Mukhopadhyay, N., & Ray, A. K. (2001). Effects of amino acid supplementation on the nutritive quality of fermented linseed meal protein in the diets for rohu, *Labeo rohita*, fingerlings. *Journal of Applied Ichthyology, 17*(5), 220–226.

Mukhopadhyay, N., & Ray, A. K. (2005). Effect of fermentation on apparent total and nutrient digestibility of linseed, *Linum usitatissimum*, meal in rohu, *Labeo rohita*, fingerlings. *Acta Ichthyologica et Piscatoria, 35*(2), 73–78.

Mzengereza, K., Ishikawa, M., Koshio, S., Yokoyama, S., Yukun, Z., Shadrack, R. S., Seo, S., Kotani, T., Dossou, S., El Basuini, M. F., & Dawood, M. A. O. (2021). Growth performance, growth-related genes, digestibility, digestive enzyme activity, immune and stress responses of de novo camelina meal in diets of red seabream (*Pagrus major*). *Animals*, *11*(11), Article 3118. [https://doi.org/10.3390/ani11113118](http://?)

Oghenochuko, O. M., Adenubi, O. T., Ajayi, O. L., Mshelbwala, F. M., Olukunle, J. O., Rahman, S. A., & Ezeri, G. N. O. (2024). Effect of Whole *Allium cepa* Linn. Bulb Slurry on Haematological and Biochemical Components of *Clarias gariepinus* Juveniles. *Pakistan Journal of Zoology*, *56*(1), 353-362. [https://doi.org/10.17582/journal.pjz/20200828170831](http://?)

Okomoda, V. T., Tiamiyu, L. O., & Akpan, I. S. (2017). Nutritional evaluation of toasted *Mucuna utilis* seed meal and its utilization in the diet of *Clarias gariepinus* (Burchell, 1822). *Journal of Applied Aquaculture*, *29*(2), 167-182. [https://doi.org/10.1080/10454438.2017.1278733](http://?)

Olude, O. O., Sahu, N. P., Nuzaiba, P. M., & Krishna, G. (2021). Feeding sundried cassava leaf meal as a replacement for de-oiled rice bran in the diets of rohu, *Labeo rohita*, fingerlings: Effect on growth, enzyme activities and gene expression of IGF-I and IGF-BPI. *Aquaculture Nutrition, 27*, 817–828. https://doi.org/10.1111/anu.13226

Olude, O. O., & Akinduti, P. (2023). Growth and Haemato-Biochemical Responses of All-Male Tilapia, *Oreochromis niloticus*, to Diets Containing Fermented Cassava Leaf Meal. In *Biotechnological Approaches to Sustainable Development Goals* (pp. 187-203). [https://doi.org/10.1007/978-3-031-33370-5_13](http://?)

Omnes, M.-H., Le Goasduff, J., Le Delliou, H., Le Bayon, N., Quazuguel, P., & Robin, J. H. (2017). Effects of dietary tannin on growth, feed utilization and digestibility, and carcass composition in juvenile European seabass (*Dicentrarchus labrax* L.). *Aquaculture Reports*, *6*, 21-27.

Orisasona, O., Adekanmbi, A. O., Akinrinade, B. P., & Taiwo, O. O. (2025). Effect of almond *Terminalia catappa* leaf extracts on growth, gut morphometry, immunocompetence and resistance of *Heterobranchus longifilis* to *Pseudomonas aeruginosa*. *Aquaculture Studies, 25*(5), 245–253. [https://doi.org/10.4194/AQUAST2394](http://?)

Peng, K., Wang, G., Wang, Y., Chen, B., Sun, Y., Mo, W., Li, G., & Huang, Y. (2020a). Condensed tannins enhanced antioxidant capacity and hypoxic stress survivability but not growth performance and fatty acid profile of juvenile Japanese seabass (*Lateolabrax japonicus*). *Animal Feed Science and Technology*, *269*. [https://doi.org/10.1016/j.anifeedsci.2020.114671](http://?)

Peng, K., Wang, G., Zhao, H., Wang, Y., Mo, W., Wu, H., & Huang, Y. (2020b). Effect of high level of carbohydrate and supplementation of condensed tannins on growth performance, serum metabolites, antioxidant and immune response, and hepatic glycometabolism gene expression of *Lateolabrax japonicus*. *Aquaculture Reports*, *18*. [https://doi.org/10.1016/j.aqrep.2020.100515](http://?)

Peng, K., Zhou, Y., Wang, Y., Wang, G., Huang, Y., & Cao, J. (2020c). Inclusion of condensed tannins in *Lateolabrax japonicus* diets: Effects on growth, nutrient digestibility, antioxidant and immune capacity and copper sulphate stress resistance. *Aquaculture Reports*, *18*. [https://doi.org/10.1016/j.aqrep.2020.100525](http://?)

Peng, K., Zhao, H. X., Wang, G. X., Chen, B., Mo, W. Y., & Huang, Y. H. (2021). Effect of condensed tannins on growth performance, intestinal immune capacity and bacterial microbiomes of *Lateolabrax japonicus*. *Aquaculture research*, *52*(11), 5321-5331. [https://doi.org/10.1111/are.15402](http://?)

Peng, K., Chen, B., Wang, Y., Zhao, H., Zheng, C., Chen, X., & Huang, W. (2022a). Condensed tannins protect against aflatoxin B1-induced toxicity in *Lateolabrax maculatus* by restoring intestinal integrity and regulating bacterial microbiota. *Aquaculture*, *555*, Article 738255. [https://doi.org/10.1016/j.aquaculture.2022.738255](http://?)

Peng, K., Chen, B., Zhao, H., Wang, Y., & Huang, W. (2022b). Condensed Tannins Improve Glycolipid Metabolism but Induce Liver Injury of Chinese Seabass (*Lateolabrax maculatus*). *Frontiers in Marine Science*, *9*, Article 902633. [https://doi.org/10.3389/fmars.2022.902633](http://?)

Peng, K., Chen, B., Zhao, H., Wang, Y., Zheng, C., Lu, H., Huang, M., Zhao, J., & Huang, W. (2022c). Reevaluation of dietary condensed tannins on growth, antioxidant and immune response and muscle quality of Chinese seabass (*Lateolabrax maculatus*). *Aquaculture*, *558*, Article 738413. [https://doi.org/10.1016/j.aquaculture.2022.738413](http://?)

Peng, K., Chen, B., Zhao, H., Zheng, C., Wang, Y., Luo, C., & Huang, W. (2022d). Condensed tannins alleviate aflatoxin B1-induced injury in Chinese sea bass (*Lateolabrax maculatus*). *Aquaculture*, *552*, Article 738029. [https://doi.org/10.1016/j.aquaculture.2022.738029](http://?)

Peng, K., Lv, X., Zhao, H., Chen, B., Chen, X., & Huang, W. (2022e). Antioxidant and intestinal recovery function of condensed tannins in *Lateolabrax maculatus* responded to in vivo and in vitro oxidative stress. *Aquaculture, 547*, 737399. [https://doi.org/10.1016/j.aquaculture.2021.737399](http://?)

Ponsin, K., Khunchalee, J., & Munglue, P. (2025). Dietary administration of karonda (*Carissa carandas*) on the growth, digestive enzymes, skin mucosal immunity, and pigmentation in Siamese fighting fish (*Betta splendens*). *Pertanika Journal of Tropical Agricultural Science, 48*(1), 259–278. [https://doi.org/10.47836/pjtas.48.1.14](http://?)

Prusty, A. K., Sahu, N. P., Pal, A. K., Reddy, A. K., & Kumar, S. (2007). Effect of dietary tannin on growth and haemato-immunological parameters of *Labeo rohita* (Hamilton) fingerlings. *Animal Feed Science and Technology, 136*(1–2), 96–108. [https://doi.org/10.1016/j.anifeedsci.2006.08.023](http://?)

Qiu, J., Huang, W., Cao, J., Zhao, H., Chen, B., Jiun-Yan, L., & Peng, K. (2023). Dietary hydrolyzable tannins reduce growth performance and induce histological damage of Chinese seabass (*Lateolabrax maculatus*). *Frontiers in Marine Science*, *10*, Article 1183438. [https://doi.org/10.3389/fmars.2023.1183438](http://?)

Qiu, J., Chen, B., Huang, W., Zhao, H., Hu, J., Loh, J. Y., & Peng, K. (2024). Dietary condensed tannin exhibits stronger growth-inhibiting effect on Chinese sea bass than hydrolysable tannin. *Animal Feed Science and Technology*, *308*, Article 115880. [https://doi.org/10.1016/j.anifeedsci.2024.115880](http://?)

Qiu, J., Chen, B., Zhao, H., Huang, W., Cao, J., Wang, G., Loh, J. Y., & Peng, K. (2024). The protective role of condensed tannins on growth inhibition and enterohepatic injury induced by histamine in Chinese seabass (*Lateolabrax maculatus*). *Animal Feed Science and Technology*, *310*, Article 115921. [https://doi.org/10.1016/j.anifeedsci.2024.115921](http://?)

Quintero Pinto, L. G., Pezzato, L. E., Miranda, E. C. de, & Barros, M. M. (2001). Desempenho do piauçu (*Leporinus macrocephalus*) arraçoado com dietas contendo diferentes teores de tanino [Growth performance of piauçu (*Leporinus macrocephalus*) fed diets with different levels of tannin]. *Revista Brasileira de Zootecnia, 30*(4), 1164–1171. [https://doi.org/10.1590/S1516-35982001000500005](http://?)

Rabelo, P. C., Pessini, J. E., Sanchez, M. S. D. S., Boscolo, W. R., Feiden, A., Bittencourt, F., & Signor, A. (2016). Sorghum in diets for silver catfish *Rhamdia quelen*. *Acta Veterinaria Brasilica, 10*(4), 339–345.

Ramachandran, S., & Ray, A. K. (2004). Inclusion of extruded grass pea, *Lathyrus sativus* seed meal in compound diets for rohu, *Labeo rohita* (Hamilton, 1822) fingerlings. *Acta Ichthyologica Et Piscatoria*, *34(2)*, 205–218.

Ramachandran, S., Bairagi, A., & Ray, A. K. (2005). Improvement of nutritive value of grass pea (*Lathyrus sativus*) seed meal in the formulated diets for rohu, *Labeo rohita* (Hamilton) fingerlings after fermentation with a fish gut bacterium. *Bioresource Technology*, *96*(13), 1465-1472. [https://doi.org/10.1016/j.biortech.2004.12.002](http://?)

Ramachandran, S., & Ray, A. K. (2007). Nutritional evaluation of fermented black gram (*Phaseolus mungo*) seed meal in compound diets for rohu, *Labeo rohita* (Hamilton), fingerlings. *Journal of Applied Ichthyology*, *23*(1), 74-79. [https://doi.org/10.1111/j.1439-0426.2006.00772.x](http://?)

Ramachandran, S., & Ray, A. (2008). Effect of Different Processing Techniques on the Nutritive Value of Grass Pea, *Lathyrus sativus* L., Seed Meal in Compound Diets for Indian Major Carp Rohu, *Labeo rohita* (Hamilton), Fingerlings. *Archives of Polish Fisheries*, *16*(2). [https://doi.org/10.2478/s10086-008-0016-2](http://?)

Raman, S., Deo, A. D., Aklakur, M., Sahu, N. P., Jayant, M., & Varghese, T. (2024). Comparative Evaluation of Dietary Raw and Solid-state Fermented Sesbania Leaf Meal in *Labeo rohita* (Hamilton, 1822). *Indian Journal of Animal Research,* *58*(3)*, 433-439*. [https://doi.org/10.18805/ijar.B-4889](http://?)

Roy, T., Banerjee, G., Dan, S. K., Ghosh, P., & Ray, A. K. (2014). Improvement of nutritive value of sesame oilseed meal in formulated diets for rohu, *Labeo rohita* (Hamilton), fingerlings after fermentation with two phytase-producing bacterial strains isolated from fish gut. *Aquaculture International*, *22*(2), 633-652. [https://doi.org/10.1007/s10499-013-9691-0](http://?)

Roy, T., Dan, S. K., Banerjee, G., Nandi, A., Ghosh, P., & Ray, A. K. (2016). Comparative Efficacy of Phytase from Fish Gut Bacteria and a Commercially Available Phytase in Improving the Nutritive Value of Sesame Oilseed Meal in Formulated Diets for Fingerlings of Rohu, *Labeo rohita* (Actinopterygii: Cypriniformes: Cyprinidae). *Acta Ichthyologica Et Piscatoria*, *46*(1), 9-23. [https://doi.org/10.3750/Aip2016.46.1.02](http://?)

Safari, R., Hoseinifar, S. H., Imanpour, M. R., Mazandarani, M., Sanchouli, H., & Paolucci, M. (2020). Effects of dietary polyphenols on mucosal and humoral immune responses, antioxidant defense and growth gene expression in beluga sturgeon (*Huso huso*). *Aquaculture*, *528*. [https://doi.org/10.1016/j.aquaculture.2020.735494](http://?)

Saha, S., & Ray, A. K. (2011). Evaluation of Nutritive Value of Water Hyacinth (*Eichhornia crassipes*) Leaf Meal in Compound Diets for Rohu, *Labeo rohita* (Hamilton, 1822) Fingerlings after Fermentation with Two Bacterial Strains Isolated from Fish Gut. *Turkish Journal of Fisheries and Aquatic Sciences*, *11*(2), 199-207. [https://doi.org/10.4194/trjfas.2011.0204](http://?)

Thitichayaphong, N., & Rukkwamsuk, T. (2016). Dietary supplementation of condensed tannin decreased lipid peroxidation in muscle of Nile tilapias (*Oreochromis niloticus*). *The Thai Journal of Veterinary Medicine, 46*(4), 649–654. [https://doi.org/10.56808/2985-1130.2784](http://?)

Vo, B. V., Siddik, M. A. B., Chaklader, M. R., Fotedar, R., Nahar, A., Foysal, M. J., Bui, D. P., & Nguyen, H. Q. (2020). Growth and health of juvenile barramundi (*Lates calcarifer*) challenged with DO hypoxia after feeding various inclusions of germinated, fermented and untreated peanut meals. *PLoS ONE*, *15*(4), e0232278. [https://doi.org/10.1371/journal.pone.0232278](http://?)

Wang, L., Zhou, H. H., He, R. J., Xu, W., Mai, K. S., & He, G. (2016). Effects of soybean meal fermentation by *Lactobacillus plantarum* P8 on growth, immune responses, and intestinal morphology in juvenile turbot (*Scophthalmus maximus* L.). *Aquaculture*, *464*, 87-94. [https://doi.org/10.1016/j.aquaculture.2016.06.026](http://?)

Wang, J., Zhang, J., Hang, Y., Xia, X., Huang, X., & Hua, X. (2025). Effects of low-dose hydrolysable tannin on growth, antioxidant capacity, and intestinal microbiota of largemouth bass. *Journal of Fisheries of China, 49*(9), 099604. [https://doi.org/10.11964/jfc.20240414472](http://?)

Xue, X., Chen, Y., Yu, Z., Feng, Y., Zhang, L., Han, C., Yin, X., Lu, B., & Shu, H. (2024). Effects of diet supplemented with hydrolyzable tannin on the growth performance, antioxidant capacity, and muscle nutritional quality of juvenile *Mastacembelus armatus*. *Aquaculture Nutrition, 2024*, Article 8266189. [https://doi.org/10.1155/2024/8266189](http://?)

Yao, J., Chen, P., Apraku, A., Zhang, G., Huang, Z., & Hua, X. (2019a). Hydrolysable tannin supplementation alters digestibility and utilization of dietary protein, lipid, and carbohydrate in grass carp (*Ctenopharyngodon idellus*). *Frontiers in Nutrition, 6*, 183. [https://doi.org/10.3389/fnut.2019.00183](http://?)

Yao, J., Chen, P., Ringø, E., Zhang, G., Huang, Z., & Hua, X. (2019b). Effect of diet supplemented with rapeseed meal or hydrolysable tannins on the growth, nutrition, and intestinal microbiota in grass carp (*Ctenopharyngodon idellus*). *Frontiers in Nutrition*, *6,* 154. [https://doi.org/10.3389/fnut.2019.00154](http://?)

Yao, J., Hang, Y., Hua, X., Li, N., & Li, X. (2022). Hepatopancreas-intestinal health in grass carp (*Ctenopharyngodon idella*) fed with hydrolyzable tannin or rapeseed meal. *Aquaculture Nutrition, 2022*, 6746201. [https://doi.org/10.1155/2022/6746201](http://?)

Yang, M., Jiang, D., Lai, W., Chen, K., Xu, S., Yu, R., Li, L., Zhang, L., Lu, L., Xu, Y., Liu, Y., & Jiang, J. (2024). Condensed tannin improves growth and alleviates intestinal inflammation of juvenile largemouth bass (*Micropterus salmoides*) fed with high cottonseed protein concentrate diet. *International Journal of Biological Macromolecules*, *280*, Article 135874. [https://doi.org/10.1016/j.ijbiomac.2024.135874](http://?)

Yang, M., Jiang, D., Zhang, L., Lu, L., Xu, Y., Khan, M. S., & Jiang, J. (2025). Dietary condensed tannin supplementation improves growth performance and feed utilization of juvenile Largemouth bass (*Micropterus salmoides*) through positively regulating serum lipids and intestinal health. *Aquaculture*, *595*, Article 741607. [https://doi.org/10.1016/j.aquaculture.2024.741607](http://?)
