# Supplementary material for: Dietary Tannins in Cultured Fish: Growth Promoter or Metabolic Burden? A Meta‐Analysis
Source: Aquac Nutr. 2026 Nov 15;2026:2460161. doi: 10.1155/anu/2460161 (PMC13770322; doi:10.1155/anu/2460161)
Supplement: Supplementary file 1 [file ANU-2026-2460161-s001.zip › Supplementary Materials/Table S3.docx]

| Table S3. Meta-analysis equations and explanations of variables used in the statistical calculations | | | |
| --- | --- | --- | --- |
| Equations | Description | Formula Summary | Where |
| (1) | Hedges’ g | $\boldsymbol{g=}\frac{{\boldsymbol{M}_{\boldsymbol{treatment}}\boldsymbol{-M}}_{\boldsymbol{ref}}}{\boldsymbol{SD}_{\boldsymbol{pooled}}}$ | $\boldsymbol{M}_{\boldsymbol{treatment}}\boldsymbol{}$ is mean of the treatment group;  $\boldsymbol{M}_{\boldsymbol{ref}}$mean of the control group (reference);  $\boldsymbol{SD}_{\boldsymbol{pooled}}$ pooled standard deviation across the treatment and control groups being compared. |
| (2) | Standard error (SE) | $SE=\sqrt{\frac{1}{\Sigma w_{i}}+{var}^{2}}$ | $\boldsymbol{w}_{\boldsymbol{i}}\boldsymbol{}$ is the weight of the $\boldsymbol{i}_{\boldsymbol{th}}$ study;  ${var}^{2}$ is the between-study variance |
| (3) | Fail-safe number (FsN) | $FsN>5k+10$ | $FsN$ is the fail-safe number;  $k$ is the number of included in the studies |
| (4) | $I^{2}$ | $I^{2}=\frac{Q-df}{Q}\times100\%$ | Q refers to Cochran’s Q statistic, which represents the total variation across studies, and df denotes the degrees of freedom, calculated as the number of studies minus one (k − 1). |
| (5) | Egger's | $Egger's=\frac{\hat{\beta}_{0}}{{SE(\hat{\beta}}_{0})},=k-2$ | $\hat{\beta}_{0}$ is the estimated intercept from Egger's regression;  SE ($\hat{\beta}_{0}$) denotes the standard error of $\hat{\beta}_{0}$;  k is the number of studies;  df is the degrees of freedom in the regression model. |
| (6) | Begg’s test | $\boldsymbol{\tau=}\frac{\boldsymbol{S}}{\sqrt{\boldsymbol{(}\boldsymbol{n}_{\boldsymbol{0}}\boldsymbol{-1)\cdot(}{\boldsymbol{2}\boldsymbol{n}}_{\boldsymbol{0}}\boldsymbol{+5)/18}}}$  $Z=\frac{\tau}{{SE}_{\tau}}$ | S is the number of concordant-discordant pairs in effect sizes vs. standard errors;  $\boldsymbol{n}_{\boldsymbol{0}}$ is the number of studies;  The Z value is then used to obtain a p value, testing the null hypothesis that there is no association between effect size and its standard error (i.e., no publication bias). |
