# Supplementary material for: Dietary Tannins in Cultured Fish: Growth Promoter or Metabolic Burden? A Meta‐Analysis
Source: Aquac Nutr. 2026 Nov 15;2026:2460161. doi: 10.1155/anu/2460161 (PMC13770322; doi:10.1155/anu/2460161)
Supplement: Supplementary file 1 [file ANU-2026-2460161-s001.zip › Supplementary Materials/Table S4.docx]

| Table S4. Complete subgroup analysis of the effects of dietary tannin supplementation across all reported outcomes in cultured fish according to tannin category. | | | | | | | | | | | | | | |
| --- | --- | --- | --- | --- | --- | --- | --- | --- | --- | --- | --- | --- | --- | --- |
| **Parameter** | **Subgroup** | **k** | ***g*** | **SE** | ***p*** | **Lower** | **Upper** | **FsN (R)** | ***I^2^*** | ***τ²*** | ***τ²′*** | **Q** | **Begg** | **Egger** |
| Specific growth rate (%g/day) | Hydrolysable | 67 | -1.12 | 0.27 | <0.001 | -1.64 | -0.59 | 138.54 | 78.98 | 2.79 | 3.55 | <0.001 | <0.001 | <0.001 |
| Specific growth rate (%g/day) | Condensed | 230 | -1.46 | 0.17 | <0.001 | -1.79 | -1.14 | 182.65 | 81.16 | 4.36 | 5.68 | <0.001 | <0.001 | <0.001 |
| Specific growth rate (%g/day) | Not specified | 4 | -0.38 | 0.71 | 0.59 | -1.78 | 1.01 | 101.34 | 67.56 | 1.36 | 1.36 | 0.03 | 0.72 | 0.18 |
| Feed Conversion Ratio | Condensed | 224 | 1.39 | 0.16 | <0.001 | 1.07 | 1.70 | 223.67 | 80.07 | 3.94 | 5.18 | <0.001 | <0.001 | <0.001 |
| Feed Conversion Ratio | Hydrolysable | 58 | 0.80 | 0.23 | <0.001 | 0.35 | 1.25 | 287.72 | 72.92 | 1.72 | 2.22 | <0.001 | <0.001 | <0.001 |
| Feed Conversion Ratio | Not specified | 4 | 0.44 | 0.67 | 0.52 | -0.88 | 1.76 | 76.14 | 64.37 | 1.16 | 1.16 | 0.04 | 1.00 | 0.05 |
| Protein efficiency ratio | Hydrolysable | 40 | -1.63 | 0.39 | <0.001 | -2.40 | -0.87 | 17.56 | 72.77 | 3.75 | 4.98 | <0.001 | <0.001 | <0.001 |
| Protein efficiency ratio | Condensed | 194 | -1.10 | 0.14 | <0.001 | -1.38 | -0.83 | 419.25 | 73.89 | 2.51 | 3.35 | <0.001 | <0.001 | <0.001 |
| Atrial natriuretic peptide (%) | Condensed | 82 | -2.82 | 0.61 | <0.001 | -4.02 | -1.61 | -42.35 | 90.36 | 22.78 | 26.29 | <0.001 | <0.001 | <0.001 |
| Atrial natriuretic peptide (%) | Hydrolysable | 20 | -3.50 | 1.44 | 0.02 | -6.33 | -0.68 | -13.74 | 83.87 | 23.81 | 23.81 | <0.001 | 0.10 | 0.22 |
| Weight gain (%) | Hydrolysable | 68 | -1.40 | 0.35 | <0.001 | -2.08 | -0.71 | 65.63 | 79.38 | 4.86 | 6.27 | <0.001 | <0.001 | <0.001 |
| Weight gain (%) | Condensed | 227 | -1.05 | 0.19 | <0.001 | -1.42 | -0.67 | 567.19 | 84.10 | 5.75 | 7.87 | <0.001 | <0.001 | <0.001 |
| Weight gain (%) | Not specified | 4 | -0.19 | 0.76 | 0.80 | -1.69 | 1.30 | 408.43 | 71.16 | 1.64 | 1.64 | 0.02 | 1.00 | 0.24 |
| Weight gain (%) | Mixed (HT + CT) | 4 | -0.80 | 0.79 | 0.31 | -2.34 | 0.74 | 20.00 | 67.46 | 1.65 | 1.65 | 0.03 | 0.33 | <0.001 |
| Body crude protein content (%) | Condensed | 177 | -0.15 | 0.16 | 0.34 | -0.47 | 0.16 | 28865.63 | 78.71 | 3.14 | 3.25 | <0.001 | 0.28 | 0.05 |
| Body crude protein content (%) | Hydrolysable | 48 | -0.23 | 0.24 | 0.34 | -0.69 | 0.24 | 3572.23 | 62.32 | 1.59 | 1.81 | <0.001 | 0.19 | 0.42 |
| Body crude lipid content (%) | Condensed | 161 | -1.04 | 0.18 | <0.001 | -1.40 | -0.68 | 413.27 | 80.85 | 3.91 | 4.94 | <0.001 | <0.001 | <0.001 |
| Body crude lipid content (%) | Hydrolysable | 48 | -0.85 | 0.29 | <0.001 | -1.41 | -0.29 | 208.47 | 69.86 | 2.58 | 2.58 | <0.001 | <0.001 | 0.49 |
| Body ash content (%) | Condensed | 165 | -0.04 | 0.12 | 0.74 | -0.28 | 0.20 | 380236.31 | 67.98 | 1.55 | 1.70 | <0.001 | <0.001 | <0.001 |
| Body ash content (%) | Hydrolysable | 48 | -0.09 | 0.14 | 0.54 | -0.37 | 0.19 | 23592.75 | 16.31 | 0.16 | 0.33 | 0.17 | 0.03 | 0.03 |
| Total carbohydrate (%) | Hydrolysable | 5 | -1.78 | 1.11 | 0.11 | -3.96 | 0.40 | 1.07 | 79.17 | 4.78 | 6.53 | <0.001 | 0.23 | 0.12 |
| Apparent protein digestibility (%) | Condensed | 109 | -1.48 | 0.21 | <0.001 | -1.89 | -1.06 | 82.99 | 77.15 | 3.36 | 5.03 | <0.001 | <0.001 | <0.001 |
| Apparent protein digestibility (%) | Hydrolysable | 15 | -4.89 | 1.16 | <0.001 | -7.16 | -2.62 | -12.59 | 78.19 | 12.74 | 15.92 | <0.001 | <0.001 | <0.001 |
| Apparent lipid digestibility (%) | Condensed | 45 | -0.84 | 0.24 | <0.001 | -1.30 | -0.38 | 198.03 | 59.44 | 2.21 | 3.54 | <0.001 | <0.001 | <0.001 |
| Apparent ash digestibility (%) | Condensed | 19 | -1.63 | 0.65 | 0.01 | -2.92 | -0.35 | 8.40 | 81.67 | 5.43 | 7.24 | <0.001 | <0.001 | <0.001 |
| Apparent phosphorous digestibility (%) | Condensed | 11 | -1.81 | 0.73 | 0.01 | -3.25 | -0.38 | 1.84 | 77.56 | 4.05 | 4.05 | <0.001 | <0.001 | <0.001 |
| Apparent calcium digestibility (%) | Condensed | 7 | -0.34 | 0.63 | 0.59 | -1.57 | 0.89 | 227.45 | 67.83 | 1.87 | 1.87 | <0.001 | 1.00 | <0.001 |
| Apparent manganese digestibility (%) | Condensed | 7 | -1.14 | 0.71 | 0.11 | -2.53 | 0.26 | 13.79 | 71.93 | 2.52 | 2.52 | <0.001 | 0.03 | <0.001 |
| Apparent copper digestibility (%) | Condensed | 7 | -0.62 | 0.72 | 0.39 | -2.03 | 0.79 | 63.68 | 73.42 | 2.64 | 2.64 | <0.001 | 0.38 | 0.55 |
| Apparent iron digestibility (%) | Condensed | 7 | -1.46 | 0.84 | 0.08 | -3.10 | 0.17 | 5.55 | 76.83 | 3.68 | 3.68 | <0.001 | 0.01 | <0.001 |
| Whole body phosphorus content (mg/g) | Condensed | 7 | -1.40 | 1.15 | 0.22 | -3.65 | 0.85 | 6.70 | 84.28 | 7.66 | 8.53 | <0.001 | 0.14 | 0.17 |
| Whole body calcium content (mg/g) | Condensed | 7 | -0.68 | 1.37 | 0.62 | -3.36 | 2.00 | 51.07 | 84.41 | 10.62 | 10.62 | <0.001 | 1.00 | <0.001 |
| Whole body manganese content (µg/g) | Condensed | 7 | -3.77 | 2.11 | 0.07 | -7.90 | 0.37 | -5.11 | 89.97 | 27.13 | 27.13 | <0.001 | <0.001 | <0.001 |
| Whole body copper content (µg/g) | Condensed | 7 | -5.87 | 1.20 | <0.001 | -8.21 | -3.52 | -6.22 | 64.14 | 5.79 | 8.60 | 0.01 | <0.001 | <0.001 |
| Whole body iron content (µg/g) | Condensed | 7 | -2.58 | 0.88 | <0.001 | -4.30 | -0.86 | -2.96 | 74.08 | 3.80 | 3.80 | <0.001 | <0.001 | <0.001 |
| Faecal phophorus concentration (mg/g) | Condensed | 7 | 4.75 | 1.23 | <0.001 | 2.34 | 7.17 | -5.81 | 76.39 | 7.05 | 8.59 | <0.001 | <0.001 | <0.001 |
| Survival Rate (%) | Hydrolysable | 33 | 0.01 | 0.21 | 0.98 | -0.41 | 0.42 | 3866868.37 | 52.30 | 0.75 | 0.75 | <0.001 | 0.29 | 0.10 |
| Survival Rate (%) | Condensed | 82 | <0.001 | 0.09 | 1.00 | -0.18 | 0.18 | 1490197010 | 12.54 | 0.09 | 0.09 | 0.18 | 0.02 | <0.001 |
| Survival Rate (%) | Not specified | 4 | 0.17 | 0.37 | 0.65 | -0.56 | 0.89 | 559.81 | <0.001 | 289.23 | 289.23 | 0.83 | 0.08 | 0.35 |
| Average daily feed intake (%g/day) | Condensed | 6 | -4.53 | 3.82 | 0.24 | -12.02 | 2.95 | -4.88 | 91.62 | <0.001 | <0.001 | <0.001 | 0.14 | 0.59 |
| Feed intake (g/fish/day) | Hydrolysable | 19 | -1.79 | 0.38 | <0.001 | -2.54 | -1.05 | 3.66 | 61.75 | 69.49 | 78.73 | <0.001 | <0.001 | <0.001 |
| Feed intake (g/fish/day) | Condensed | 71 | -0.59 | 0.21 | <0.001 | -0.99 | -0.18 | 717.66 | 70.37 | 1.58 | 2.78 | <0.001 | <0.001 | <0.001 |
| Feed intake (g/fish/day) | Not specified | 3 | -0.30 | 0.75 | 0.69 | -1.76 | 1.16 | 124.19 | 65.32 | 1.97 | 2.22 | 0.06 | 1.00 | 0.02 |
| Condition factor (g/cm3) | Hydrolysable | 15 | 0.59 | 0.40 | 0.13 | -0.18 | 1.37 | 147.86 | 82.88 | 1.09 | 1.09 | <0.001 | 0.73 | 0.54 |
| Condition factor (g/cm3) | Condensed | 24 | <0.001 | 0.26 | 0.99 | -0.51 | 0.52 | 4531867.75 | 56.45 | 1.76 | 1.69 | <0.001 | 0.02 | <0.001 |
| Hepatosomatic Index (%) | Hydrolysable | 27 | -0.35 | 0.30 | 0.24 | -0.93 | 0.23 | 837.11 | 62.80 | 0.87 | 1.57 | <0.001 | 0.13 | <0.001 |
| Hepatosomatic Index (%) | Condensed | 57 | -0.42 | 0.13 | <0.001 | -0.67 | -0.17 | 1174.88 | 24.29 | 1.39 | 2.08 | 0.05 | <0.001 | <0.001 |
| Viscerosomatic index (%) | Condensed | 24 | 0.18 | 0.16 | 0.25 | -0.13 | 0.49 | 2764.00 | <0.001 | 0.21 | 0.68 | 0.58 | 0.57 | 0.01 |
| Viscerosomatic index (%) | Hydrolysable | 24 | -1.12 | 0.32 | <0.001 | -1.75 | -0.48 | 50.10 | 64.55 | <0.001 | <0.001 | <0.001 | <0.001 | <0.001 |
| Intestinosomatic index (%) | Condensed | 20 | -0.12 | 0.18 | 0.52 | -0.47 | 0.24 | 5775.07 | <0.001 | 1.52 | 1.52 | 0.79 | 0.29 | 0.16 |
| Intestinosomatic index (%) | Hydrolysable | 10 | -0.26 | 0.24 | 0.28 | -0.73 | 0.21 | 570.73 | <0.001 | <0.001 | <0.001 | 0.95 | 0.86 | 0.86 |
| ILI (%) | Hydrolysable | 6 | 0.11 | 0.58 | 0.84 | -1.03 | 1.26 | 1746.76 | 59.73 | <0.001 | <0.001 | 0.03 | 1.00 | 0.05 |
| IPF (%) | Hydrolysable | 5 | -2.00 | 0.82 | 0.01 | -3.60 | -0.40 | -0.20 | 64.49 | 1.20 | 1.20 | 0.02 | 0.02 | <0.001 |
| IPF (%) | Condensed | 8 | -1.64 | 0.54 | <0.001 | -2.71 | -0.58 | 3.38 | 67.48 | 2.03 | 2.03 | <0.001 | <0.001 | <0.001 |
| Total Antioxidant Capacity (U/mL) | Condensed | 37 | 0.73 | 0.28 | 0.01 | 0.19 | 1.27 | 229.29 | 71.94 | 1.54 | 1.54 | <0.001 | 0.06 | 0.07 |
| Total Antioxidant Capacity (U/mL) | Hydrolysable | 13 | 1.27 | 0.70 | 0.07 | -0.10 | 2.64 | 17.85 | 80.03 | 1.95 | 2.31 | <0.001 | 0.06 | 0.06 |
| Total Cholesterol (mmol/L) | Condensed | 40 | -0.94 | 0.18 | <0.001 | -1.29 | -0.58 | 134.93 | 39.82 | 4.72 | 6.10 | 0.01 | <0.001 | <0.001 |
| Total Cholesterol (mmol/L) | Hydrolysable | 13 | -0.72 | 0.69 | 0.30 | -2.09 | 0.64 | 82.42 | 82.07 | 0.50 | 1.21 | <0.001 | 0.16 | <0.001 |
| Triacylglycerol (mmol/L) | Condensed | 16 | -0.95 | 0.25 | <0.001 | -1.44 | -0.46 | 52.54 | 38.41 | 4.46 | 4.46 | 0.06 | <0.001 | <0.001 |
| Triacylglycerol (mmol/L) | Hydrolysable | 4 | -0.48 | 0.45 | 0.29 | -1.37 | 0.41 | 62.15 | 32.45 | 0.38 | 0.38 | 0.22 | 0.06 | 0.04 |
| Glucose (mmol/L) | Condensed | 40 | -0.04 | 0.24 | 0.88 | -0.51 | 0.43 | 111649.06 | 76.89 | 1.55 | 1.80 | <0.001 | 0.87 | 0.18 |
| Glucose (mmol/L) | Hydrolysable | 5 | -0.34 | 0.33 | 0.30 | -0.98 | 0.31 | 165.29 | <0.001 | 0.27 | 0.27 | 1.00 | 0.02 | 0.77 |
| Glucose (mmol/L) | Not specified | 3 | -0.89 | 0.43 | 0.04 | -1.73 | -0.05 | 11.60 | <0.001 | <0.001 | <0.001 | 0.84 | 0.33 | 0.55 |
| Blood urea nitrogen (mmol/L) | Condensed | 16 | 0.86 | 0.30 | <0.001 | 0.26 | 1.45 | 68.05 | 76.27 | <0.001 | <0.001 | <0.001 | 0.52 | 0.26 |
| Blood urea nitrogen (mmol/L) | Hydrolysable | 8 | -4.29 | 1.41 | <0.001 | -7.06 | -1.52 | -6.33 | 88.96 | 1.05 | 1.35 | <0.001 | 0.01 | <0.001 |
| Diamine oxide (U/L) | Condensed | 15 | -0.07 | 0.46 | 0.88 | -0.97 | 0.84 | 12647.25 | 76.62 | 9.39 | 13.13 | <0.001 | 1.00 | 0.23 |
| Lactate (µmol/L) | Condensed | 8 | -0.33 | 0.26 | 0.21 | -0.84 | 0.18 | 274.42 | 1.73 | 2.37 | 2.37 | 0.42 | 0.06 | 0.06 |
| Lipopolysaccharide (pg/mL) | Condensed | 11 | -0.69 | 0.53 | 0.19 | -1.73 | 0.35 | 77.02 | 75.27 | 0.01 | 0.01 | <0.001 | 0.45 | 0.03 |
| Superoxide dismutase (U/mL) | Hydrolysable | 26 | -0.10 | 0.48 | 0.84 | -1.04 | 0.84 | 10035.25 | 84.16 | 2.22 | 3.09 | <0.001 | 0.46 | 0.84 |
| Superoxide dismutase (U/mL) | Condensed | 55 | 0.92 | 0.28 | <0.001 | 0.37 | 1.47 | 193.43 | 79.24 | 4.03 | 4.03 | <0.001 | <0.001 | <0.001 |
| Catalase activity (kU/L) | Hydrolysable | 23 | 1.24 | 0.44 | <0.001 | 0.38 | 2.10 | 34.37 | 81.14 | 2.97 | 3.56 | <0.001 | <0.001 | 0.01 |
| Catalase activity (kU/L) | Condensed | 51 | 0.94 | 0.21 | <0.001 | 0.53 | 1.35 | 172.52 | 67.31 | 2.99 | 3.75 | <0.001 | <0.001 | <0.001 |
| Glutathione S-transferase (pmol/min/mg protein) | Condensed | 8 | 0.01 | 0.20 | 0.95 | -0.38 | 0.40 | 216936.86 | <0.001 | 1.37 | 1.88 | 0.56 | 0.40 | 0.02 |
| Glutathione S-transferase (pmol/min/mg protein) | Hydrolysable | 6 | 1.60 | 0.98 | 0.10 | -0.33 | 3.52 | 3.04 | 79.18 | <0.001 | 0.03 | <0.001 | 0.06 | <0.001 |
| Glutathione peroxidase (U/mL) | Condensed | 23 | 0.18 | 0.16 | 0.27 | -0.13 | 0.49 | 2840.86 | <0.001 | 4.25 | 5.43 | 0.97 | 0.22 | 0.47 |
| Malondialdehyde (U/mL) | Condensed | 40 | -0.08 | 0.20 | 0.69 | -0.47 | 0.31 | 23640.18 | 55.88 | <0.001 | <0.001 | <0.001 | 0.54 | 0.13 |
| Malondialdehyde (U/mL) | Hydrolysable | 13 | 0.99 | 0.76 | 0.19 | -0.49 | 2.48 | 37.47 | 86.81 | 0.86 | 0.86 | <0.001 | 0.01 | <0.001 |
| TBARS (nmol/g) | Hydrolysable | 3 | 2.00 | 0.80 | 0.01 | 0.42 | 3.57 | -0.11 | 42.70 | 3.98 | 5.58 | 0.17 | 0.33 | 0.06 |
| TBARS (nmol/g) | Condensed | 8 | 0.25 | 0.43 | 0.56 | -0.58 | 1.08 | 486.95 | 71.48 | 0.83 | 2.74 | <0.001 | 0.06 | 0.26 |
| Lysozyme activity (U/mL) | Condensed | 36 | 1.27 | 0.37 | <0.001 | 0.55 | 2.00 | 49.28 | 82.25 | 0.94 | 0.94 | <0.001 | 0.15 | <0.001 |
| Lysozyme activity (U/mL) | Hydrolysable | 3 | 1.82 | 0.71 | 0.01 | 0.43 | 3.20 | 0.48 | 38.69 | 3.84 | 3.84 | 0.20 | 1.00 | 0.09 |
| Peroxidase activity (OD 450 nm) | Condensed | 7 | -1.36 | 0.32 | <0.001 | -1.99 | -0.74 | 7.49 | 41.21 | 0.58 | 0.58 | 0.12 | 0.03 | 0.12 |
| Plasma bactericidal activity | Condensed | 5 | 0.93 | 0.49 | 0.06 | -0.02 | 1.89 | 17.10 | 74.32 | 0.28 | 0.28 | <0.001 | 0.08 | 0.02 |
| Nitro blue tetrazolium (OD 540 nm) | Condensed | 7 | -0.49 | 0.54 | 0.36 | -1.54 | 0.56 | 105.65 | 79.89 | 0.88 | 0.88 | <0.001 | 0.22 | 0.02 |
| Nitro blue tetrazolium (OD 540 nm) | Hydrolysable | 5 | 0.52 | 0.96 | 0.59 | -1.36 | 2.39 | 67.09 | 74.49 | 3.48 | 5.61 | <0.001 | 0.03 | <0.001 |
| Amylase (U/mg protein) | Condensed | 65 | -1.09 | 0.29 | <0.001 | -1.65 | -0.53 | 146.37 | 79.57 | 3.66 | 3.76 | <0.001 | <0.001 | 0.01 |
| Amylase (U/mg protein) | Hydrolysable | 25 | -0.13 | 0.35 | 0.71 | -0.82 | 0.56 | 5741.09 | 74.05 | 2.10 | 2.10 | <0.001 | 0.01 | <0.001 |
| Amylase (U/mg protein) | Not specified | 3 | -2.70 | 0.57 | <0.001 | -3.82 | -1.58 | -1.42 | <0.001 | <0.001 | 0.70 | 0.47 | 0.33 | 0.22 |
| Lipase (U/mg protein) | Condensed | 58 | -0.86 | 0.33 | 0.01 | -1.50 | -0.22 | 241.73 | 81.73 | 4.45 | 5.55 | <0.001 | <0.001 | 0.48 |
| Lipase (U/mg protein) | Hydrolysable | 25 | -0.23 | 0.32 | 0.46 | -0.86 | 0.39 | 1724.62 | 71.23 | 1.75 | 1.75 | <0.001 | 0.03 | 0.22 |
| Lipase (U/mg protein) | Not specified | 3 | -0.93 | 0.94 | 0.32 | -2.78 | 0.92 | 10.31 | 75.32 | 2.00 | 2.00 | 0.02 | 0.33 | 0.01 |
| Trypsin (U/mg protein) | Condensed | 17 | -0.91 | 0.56 | 0.10 | -2.00 | 0.19 | 62.44 | 84.03 | 4.08 | 4.08 | <0.001 | 0.03 | <0.001 |
| Trypsin (U/mg protein) | Hydrolysable | 14 | -0.13 | 0.79 | 0.87 | -1.67 | 1.42 | 3351.76 | 86.33 | 7.15 | 7.15 | <0.001 | 0.91 | 0.07 |
| Creatine kinase (U/mg protein) | Condensed | 5 | -0.43 | 1.01 | 0.67 | -2.41 | 1.56 | 99.84 | 81.93 | 3.53 | 6.54 | <0.001 | 0.79 | <0.001 |
| Na+, K+- ATPase (U/mg protein) | Condensed | 5 | -5.41 | 2.33 | 0.02 | -9.96 | -0.85 | -4.34 | 86.32 | 15.68 | 24.62 | <0.001 | 0.02 | <0.001 |
| Protease (U/mg protein) | Condensed | 45 | -1.20 | 0.35 | <0.001 | -1.88 | -0.52 | 75.28 | 77.98 | 3.74 | 4.19 | <0.001 | <0.001 | <0.001 |
| Protease (U/mg protein) | Hydrolysable | 8 | -0.32 | 0.75 | 0.67 | -1.79 | 1.15 | 297.26 | 83.46 | 3.70 | 3.70 | <0.001 | 0.01 | <0.001 |
| Pepsin (U/mg protein) | Condensed | 4 | -1.59 | 0.71 | 0.02 | -2.98 | -0.21 | 2.05 | 49.69 | 0.98 | 0.98 | 0.11 | 0.08 | 0.02 |
| Pepsin (U/mg protein) | Hydrolysable | 9 | -2.79 | 0.77 | <0.001 | -4.31 | -1.28 | -4.57 | 72.76 | 3.54 | 5.10 | <0.001 | <0.001 | <0.001 |
| Hematocrit (%) | Condensed | 23 | 0.48 | 0.27 | 0.08 | -0.05 | 1.02 | 353.26 | 80.85 | 1.24 | 1.24 | <0.001 | 0.10 | 0.21 |
| Creatinine (mg/dL) | Not specified | 3 | 10.47 | 3.35 | <0.001 | 3.91 | 17.03 | -2.89 | 46.72 | 15.76 | 28.84 | 0.15 | 0.33 | 0.05 |
| Total bilirubin (mg/dL) | Condensed | 6 | 0.04 | 0.33 | 0.91 | -0.62 | 0.69 | 17600.99 | <0.001 | <0.001 | <0.001 | 0.99 | 0.70 | 0.74 |
| Total bilirubin (mg/dL) | Not specified | 3 | -0.96 | 1.20 | 0.43 | -3.31 | 1.40 | 9.58 | 64.16 | 2.68 | 2.68 | 0.06 | 1.00 | 0.02 |
| Total protein (g/dL) | Condensed | 36 | -0.16 | 0.28 | 0.56 | -0.71 | 0.38 | 5130.54 | 83.43 | 2.12 | 2.66 | <0.001 | 0.20 | <0.001 |
| Total protein (g/dL) | Hydrolysable | 17 | 0.34 | 0.56 | 0.55 | -0.76 | 1.43 | 558.53 | 80.31 | 4.05 | 4.05 | <0.001 | 0.77 | 0.03 |
| Total protein (g/dL) | Not specified | 3 | -0.86 | 0.98 | 0.38 | -2.78 | 1.07 | 12.75 | 52.94 | 1.52 | 1.52 | 0.12 | 0.33 | 0.05 |
| Triglyceride (g/dL) | Condensed | 12 | -1.04 | 0.36 | <0.001 | -1.74 | -0.35 | 30.39 | 42.70 | 0.64 | 0.81 | 0.06 | <0.001 | <0.001 |
| Triglyceride (g/dL) | Hydrolysable | 11 | 0.31 | 1.04 | 0.77 | -1.73 | 2.35 | 426.21 | 86.29 | 8.45 | 11.38 | <0.001 | 0.45 | <0.001 |
| Phosphate (mmol/L) | Condensed | 5 | -0.55 | 0.37 | 0.14 | -1.28 | 0.18 | 58.02 | <0.001 | <0.001 | <0.001 | 0.98 | 0.02 | 0.51 |
| Alkaline phosphatase (IU/L) | Condensed | 26 | 0.45 | 0.17 | 0.01 | 0.11 | 0.78 | 475.84 | 35.24 | 0.24 | 0.24 | 0.04 | 0.54 | 0.96 |
| Alkaline phosphatase (IU/L) | Not specified | 3 | -6.27 | 1.72 | <0.001 | -9.64 | -2.90 | -2.71 | 22.78 | 2.17 | 7.01 | 0.27 | 0.33 | 0.11 |
| Alkaline phosphatase (IU/L) | Hydrolysable | 6 | 3.02 | 1.16 | 0.01 | 0.74 | 5.29 | -3.47 | 81.05 | 5.59 | 8.27 | <0.001 | 0.06 | <0.001 |
| Alanine aminotransferase (IU/L) | Condensed | 48 | 0.24 | 0.19 | 0.21 | -0.13 | 0.60 | 3288.64 | 62.90 | 0.94 | 0.94 | <0.001 | 0.52 | 0.09 |
| Alanine aminotransferase (IU/L) | Hydrolysable | 16 | 1.04 | 0.40 | 0.01 | 0.25 | 1.82 | 41.29 | 76.83 | 1.62 | 2.41 | <0.001 | <0.001 | <0.001 |
| Alanine aminotransferase (IU/L) | Not specified | 3 | -4.38 | 1.10 | <0.001 | -6.53 | -2.23 | -2.40 | <0.001 | <0.001 | 0.56 | 0.51 | 0.33 | 0.25 |
| Aspartate aminotransferase (IU/L) | Condensed | 43 | 0.59 | 0.28 | 0.03 | 0.05 | 1.13 | 433.68 | 77.78 | 2.16 | 2.44 | <0.001 | 0.03 | <0.001 |
| Aspartate aminotransferase (IU/L) | Hydrolysable | 16 | 0.65 | 0.34 | 0.06 | -0.03 | 1.32 | 130.44 | 71.11 | 1.14 | 1.30 | <0.001 | 0.06 | 0.38 |
| Aspartate aminotransferase (IU/L) | Not specified | 3 | -2.93 | 1.05 | 0.01 | -5.00 | -0.86 | -1.66 | 28.89 | 1.01 | 1.01 | 0.25 | 0.33 | 0.09 |
| Lactate dehydrogenase (U/mg protein) | Condensed | 15 | 0.02 | 0.24 | 0.94 | -0.45 | 0.48 | 158291.58 | 47.92 | 0.34 | 0.76 | 0.02 | 0.05 | 0.01 |
| Malate dehydrogenase (U/mg protein) | Condensed | 3 | 4.89 | 1.92 | 0.01 | 1.13 | 8.66 | -2.52 | 72.53 | 7.89 | 13.49 | 0.03 | 0.33 | 0.01 |
| Red blood cell (x10^12^L) | Condensed | 21 | 0.08 | 0.26 | 0.76 | -0.43 | 0.59 | 12250.02 | 79.38 | 1.04 | 1.04 | <0.001 | 0.70 | 0.92 |
| Red blood cell (x10^12^L) | Hydrolysable | 7 | -0.80 | 0.86 | 0.35 | -2.48 | 0.88 | 34.68 | 76.75 | 3.37 | 3.37 | <0.001 | 0.56 | <0.001 |
| Red blood cell (x10^12^L) | Not specified | 3 | 1.84 | 0.70 | 0.01 | 0.46 | 3.21 | 0.42 | <0.001 | <0.001 | <0.001 | 0.60 | 0.33 | 0.31 |
| White blood cell (x10^12^L) | Condensed | 21 | 1.23 | 0.42 | <0.001 | 0.40 | 2.06 | 32.43 | 90.18 | 3.18 | 3.18 | <0.001 | 0.03 | 0.03 |
| White blood cell (x10^12^L) | Hydrolysable | 7 | 0.15 | 0.31 | 0.64 | -0.47 | 0.76 | 1219.53 | <0.001 | <0.001 | <0.001 | 0.91 | 0.38 | 0.29 |
| WBC (x 10^12^L) | Not specified | 3 | 0.02 | 0.58 | 0.97 | -1.12 | 1.16 | 29070.82 | <0.001 | <0.001 | <0.001 | 0.84 | 1.00 | 0.79 |
| Haemoglobin (g/dL) | Condensed | 28 | 0.51 | 0.25 | 0.04 | 0.03 | 0.99 | 381.04 | 78.02 | 1.15 | 1.15 | <0.001 | 0.19 | 0.04 |
| Haemoglobin (g/dL) | Hydrolysable | 10 | -2.98 | 0.93 | <0.001 | -4.81 | -1.16 | -5.68 | 80.56 | 5.75 | 8.68 | <0.001 | <0.001 | <0.001 |
| Haemoglobin (g/dL) | Not specified | 3 | 2.67 | 0.81 | <0.001 | 1.09 | 4.25 | -1.38 | <0.001 | <0.001 | <0.001 | 0.70 | 0.33 | 0.39 |
| Platelet (%) | Condensed | 4 | 0.29 | 0.41 | 0.48 | -0.52 | 1.10 | 177.01 | <0.001 | <0.001 | <0.001 | 0.90 | 0.08 | 0.50 |
| Platelet (%) | Hydrolysable | 5 | 0.74 | 0.54 | 0.17 | -0.32 | 1.80 | 30.04 | 43.84 | 0.63 | 0.63 | 0.13 | 0.08 | 0.01 |
| Packed Cell Volume (%) | Condensed | 7 | -2.73 | 0.74 | <0.001 | -4.17 | -1.28 | -3.38 | 68.63 | 2.54 | 3.01 | <0.001 | <0.001 | <0.001 |
| Packed Cell Volume (%) | Not specified | 3 | 3.18 | 1.57 | 0.04 | 0.09 | 6.26 | -1.86 | 59.57 | 4.06 | 4.06 | 0.08 | 0.33 | 0.03 |
| Packed Cell Volume (%) | Hydrolysable | 4 | -1.42 | 1.00 | 0.16 | -3.38 | 0.54 | 3.60 | 73.42 | 2.82 | 2.82 | 0.01 | 0.07 | <0.001 |
| Mean corpuscular volume (fL) | Condensed | 24 | -0.20 | 0.28 | 0.47 | -0.75 | 0.34 | 2201.97 | 82.35 | 1.41 | 1.41 | <0.001 | 0.10 | 0.04 |
| Mean corpuscular volume (fL) | Not specified | 3 | -1.05 | 0.62 | 0.09 | -2.25 | 0.16 | 7.53 | <0.001 | <0.001 | <0.001 | 0.99 | 0.33 | 0.87 |
| Mean corpuscular volume (fL) | Hydrolysable | 6 | 0.99 | 1.02 | 0.33 | -1.01 | 2.98 | 17.72 | 80.63 | 4.62 | 4.62 | <0.001 | 0.47 | <0.001 |
| Melanine Concentrating Hormone | Condensed | 24 | 0.28 | 0.33 | 0.41 | -0.38 | 0.93 | 1183.26 | 86.47 | 2.13 | 3.02 | <0.001 | 0.06 | 0.09 |
| Melanine Concentrating Hormone | Not specified | 3 | -0.90 | 0.61 | 0.14 | -2.09 | 0.29 | 11.17 | <0.001 | <0.001 | <0.001 | 0.92 | 0.33 | 0.68 |
| Melanine Concentrating Hormone | Hydrolysable | 6 | 0.39 | 1.00 | 0.70 | -1.57 | 2.34 | 149.16 | 81.06 | 4.70 | 4.70 | <0.001 | 0.47 | 0.07 |
| Major Histocompatibility complex | Condensed | 24 | 0.42 | 0.23 | 0.07 | -0.03 | 0.87 | 497.38 | 74.51 | 0.85 | 1.26 | <0.001 | 0.68 | 0.42 |
| Major Histocompatibility complex | Not specified | 3 | 1.29 | 0.64 | 0.04 | 0.05 | 2.54 | 3.90 | <0.001 | <0.001 | <0.001 | 0.98 | 0.33 | 0.84 |
| Major Histocompatibility complex | Hydrolysable | 6 | -0.21 | 0.34 | 0.54 | -0.87 | 0.46 | 523.87 | <0.001 | <0.001 | <0.001 | 0.82 | 0.27 | 0.33 |
| Neutrophils | Condensed | 10 | 0.03 | 0.25 | 0.92 | -0.46 | 0.51 | 56370.18 | <0.001 | <0.001 | <0.001 | 0.99 | 0.02 | 0.38 |
| Neutrophils | Not specified | 3 | 2.00 | 1.20 | 0.10 | -0.36 | 4.36 | -0.13 | 57.66 | 2.48 | 5.97 | 0.09 | 0.33 | 0.03 |
| Lymphocytes | Condensed | 10 | 0.29 | 0.26 | 0.26 | -0.22 | 0.79 | 455.57 | 0.83 | 0.01 | 0.01 | 0.43 | 0.01 | 0.01 |
| Lymphocytes | Not specified | 3 | -1.50 | 0.66 | 0.02 | -2.80 | -0.20 | 2.12 | <0.001 | <0.001 | <0.001 | 0.69 | 0.33 | 0.39 |
| Lymphocytes | Hydrolysable | 6 | -0.29 | 0.56 | 0.60 | -1.38 | 0.80 | 262.59 | 56.40 | 1.04 | 1.04 | 0.04 | 0.47 | 0.35 |
| Basophils | Condensed | 7 | 0.01 | 0.29 | 0.97 | -0.56 | 0.58 | 311892.05 | <0.001 | <0.001 | <0.001 | 0.99 | 0.05 | 0.56 |
| Basophils | Not specified | 3 | -0.33 | 0.61 | 0.59 | -1.52 | 0.86 | 102.76 | <0.001 | <0.001 | <0.001 | 0.37 | 1.00 | 0.17 |
| Basophils | Hydrolysable | 4 | 0.19 | 0.41 | 0.64 | -0.61 | 1.00 | 405.45 | <0.001 | <0.001 | <0.001 | 0.87 | 0.08 | 0.40 |
| Monocytes | Condensed | 10 | 0.02 | 0.25 | 0.92 | -0.46 | 0.51 | 66367.70 | <0.001 | <0.001 | <0.001 | 1.00 | 1.00 | 0.95 |
| Monocytes | Not specified | 3 | 0.22 | 0.60 | 0.71 | -0.95 | 1.39 | 237.04 | <0.001 | <0.001 | <0.001 | 0.53 | 0.16 | 0.26 |
| Monocytes | Hydrolysable | 6 | 1.38 | 0.38 | <0.001 | 0.64 | 2.11 | 6.16 | <0.001 | <0.001 | <0.001 | 0.88 | <0.001 | 0.19 |
| Erythrocytes | Condensed | 7 | 0.28 | 0.27 | 0.31 | -0.26 | 0.82 | 334.42 | <0.001 | <0.001 | <0.001 | 0.58 | 0.07 | 0.04 |
| Erythrocytes | Not specified | 3 | 0.46 | 0.61 | 0.45 | -0.73 | 1.64 | 51.70 | <0.001 | <0.001 | <0.001 | 0.46 | 0.16 | 0.21 |
| Erythrocytes | Hydrolysable | 5 | -0.49 | 3.38 | 0.89 | -7.11 | 6.14 | 75.97 | 88.90 | 22.18 | 37.41 | <0.001 | 0.19 | 0.04 |
| Thrombocytes | Condensed | 3 | 0.45 | 0.42 | 0.28 | -0.36 | 1.27 | 53.38 | <0.001 | <0.001 | <0.001 | 0.69 | 0.33 | 0.40 |
| Leukocytes | Condensed | 7 | 1.39 | 0.56 | 0.01 | 0.30 | 2.49 | 6.89 | 67.33 | 1.44 | 1.98 | 0.01 | 0.03 | <0.001 |
| Leukocytes | Hydrolysable | 5 | 5.96 | 1.78 | <0.001 | 2.47 | 9.45 | -4.46 | 77.07 | 11.42 | 11.15 | <0.001 | 0.02 | <0.001 |
| Eosinophils | Condensed | 7 | <0.001 | 0.31 | 0.99 | -0.60 | 0.61 | 1765944.12 | <0.001 | <0.001 | <0.001 | 1.00 | 1.00 | 0.81 |
| Eosinophils | Hydrolysable | 6 | -0.74 | 0.35 | 0.04 | -1.44 | -0.05 | 35.63 | <0.001 | <0.001 | 0.21 | 0.54 | <0.001 | 0.05 |
| Villus length (cm) | Condensed | 40 | 0.03 | 0.31 | 0.91 | -0.58 | 0.65 | 127439.52 | 80.07 | 2.96 | 3.68 | <0.001 | <0.001 | 0.29 |
| Villus length (cm) | Hydrolysable | 4 | 0.57 | 1.04 | 0.58 | -1.47 | 2.61 | 42.88 | 90.28 | 3.82 | 3.82 | <0.001 | 1.00 | 0.21 |
| Villus width (cm) | Condensed | 40 | -0.53 | 0.29 | 0.07 | -1.09 | 0.04 | 516.05 | 77.16 | 2.38 | 2.38 | <0.001 | 0.21 | <0.001 |
| Villi density (mm^2^) | Condensed | 10 | 1.32 | 0.54 | 0.01 | 0.27 | 2.37 | 11.98 | 87.55 | 2.47 | 2.47 | <0.001 | 0.03 | <0.001 |
| Muscular thickness (cm) | Condensed | 9 | 0.11 | 0.24 | 0.65 | -0.36 | 0.58 | 2927.46 | <0.001 | <0.001 | <0.001 | 0.85 | 0.36 | 0.78 |
| Goblet cell number | Hydrolysable | 3 | 2.15 | 1.23 | 0.08 | -0.26 | 4.55 | -0.50 | 91.18 | 4.09 | 4.09 | <0.001 | 0.33 | <0.001 |
| Goblet cell number | Condensed | 11 | 0.73 | 0.40 | 0.07 | -0.05 | 1.51 | 67.90 | 55.24 | 0.93 | 1.21 | 0.01 | <0.001 | <0.001 |
| Albumin (g/L) | Condensed | 21 | -0.08 | 0.26 | 0.77 | -0.59 | 0.44 | 13792.20 | 66.90 | 0.86 | 1.49 | <0.001 | 0.01 | <0.001 |
| Albumin (g/L) | Hydrolysable | 21 | -0.19 | 0.31 | 0.53 | -0.80 | 0.42 | 2139.76 | 62.47 | 1.23 | 1.23 | <0.001 | 0.61 | 0.08 |
| Albumin (g/L) | Not specified | 3 | 0.85 | 0.62 | 0.17 | -0.37 | 2.07 | 13.10 | <0.001 | <0.001 | <0.001 | 0.48 | 0.33 | 0.24 |
| Globulin (g/L) | Condensed | 15 | 0.06 | 0.21 | 0.78 | -0.35 | 0.46 | 17564.48 | 5.59 | 0.04 | 0.04 | 0.39 | 0.85 | 0.25 |
| Globulin (g/L) | Hydrolysable | 16 | 0.27 | 0.60 | 0.65 | -0.90 | 1.45 | 807.80 | 81.54 | 4.11 | 4.11 | <0.001 | 0.50 | 0.01 |
| Globulin (g/L) | Not specified | 3 | -1.86 | 1.08 | 0.08 | -3.98 | 0.25 | 0.31 | 48.88 | 1.68 | 1.68 | 0.14 | 0.33 | 0.05 |
| HDLC (mmol/L) | Condensed | 18 | -0.07 | 0.21 | 0.75 | -0.49 | 0.35 | 15053.04 | 27.41 | 0.22 | 0.22 | 0.14 | 0.34 | 0.01 |
| HDLC (mmol/L) | Hydrolysable | 7 | -1.64 | 1.48 | 0.27 | -4.55 | 1.26 | 2.96 | 90.67 | 13.10 | 14.62 | <0.001 | 0.07 | 0.02 |
| LDLC (mmol/L) | Condensed | 18 | 0.20 | 0.30 | 0.49 | -0.37 | 0.78 | 1641.83 | 58.21 | 0.89 | 0.89 | <0.001 | 0.52 | 0.01 |
| LDLC (mmol/L) | Hydrolysable | 7 | -0.73 | 0.89 | 0.41 | -2.47 | 1.02 | 43.77 | 83.66 | 4.12 | 4.12 | <0.001 | 0.76 | <0.001 |
| Insulin (pg/mL) | Condensed | 3 | 0.98 | 0.91 | 0.28 | -0.79 | 2.75 | 8.98 | 70.50 | 1.72 | 1.72 | 0.03 | 0.33 | 0.01 |
| Glucagon (ng/mL) | Condensed | 3 | -0.16 | 0.43 | 0.70 | -1.00 | 0.68 | 425.92 | <0.001 | <0.001 | <0.001 | 0.88 | 1.00 | 0.84 |
| TNF-α (ng/L) | Condensed | 8 | -1.62 | 0.29 | <0.001 | -2.19 | -1.05 | 3.72 | <0.001 | <0.001 | <0.001 | 0.95 | <0.001 | 0.14 |
| Nrf2 (ng/L) | Hydrolysable | 3 | 2.35 | 0.53 | <0.001 | 1.31 | 3.40 | -0.92 | <0.001 | <0.001 | <0.001 | 0.92 | 0.33 | 0.68 |
| Keap1 | Hydrolysable | 3 | 6.77 | 1.28 | <0.001 | 4.26 | 9.28 | -2.75 | 26.72 | 1.37 | 5.76 | 0.26 | 0.33 | 0.10 |
| IL-6 (ng/L) | Condensed | 10 | -1.42 | 0.66 | 0.03 | -2.72 | -0.12 | 9.07 | 80.49 | 3.33 | 5.04 | <0.001 | 0.05 | <0.001 |
| IL-8 (ng/L) | Condensed | 8 | 0.69 | 0.26 | 0.01 | 0.18 | 1.20 | 57.20 | <0.001 | <0.001 | <0.001 | 0.83 | <0.001 | 0.07 |
| IL-8 (ng/L) | Hydrolysable | 3 | 2.01 | 0.50 | <0.001 | 1.02 | 2.99 | -0.13 | <0.001 | <0.001 | <0.001 | 0.89 | 0.33 | 0.62 |
| IL-10 (ng/L) | Condensed | 5 | 0.36 | 0.32 | 0.26 | -0.27 | 0.99 | 141.49 | <0.001 | <0.001 | <0.001 | 0.90 | 0.02 | 0.33 |
| IL-10 (ng/L) | Hydrolysable | 3 | 3.26 | 0.93 | <0.001 | 1.45 | 5.08 | -1.92 | 50.03 | 1.27 | 1.27 | 0.14 | 0.33 | 0.05 |
| Immune globulin (µg/mg) | Condensed | 15 | 0.69 | 0.29 | 0.02 | 0.13 | 1.25 | 105.52 | 46.25 | 0.56 | 0.56 | 0.03 | 0.01 | <0.001 |

*g* – Hedges’ *g*; *p* – significance value for the pooled effect size, Q – significance value from Cochran’s Q test for heterogeneity, Begg – significance value from Begg’s test, Egger – significance value from Egger’s regression test, FSN – fail state number, cm – centimetre, cm^3^ – cubic centimetre, dL – decilitre, fL – femtoliter, FSN – fail state number, HDLC – high density lipoprotein cholesterol, kU – kilounit, µg – microgram, g – gram, IL – interleukin, *I²* – heterogeneity, *τ²* – estimated between-study variance from the original random-effects model; *τ²′* – estimated between-study variance from the corresponding adjusted/prime random-effects model; K+ - Kalium, k – number of studies, IU – international unit, L – litre, mg – milligram, LDLC – low density lipoprotein cholesterol, mL – mililiter, mmol – milimol, mm^2^ – square millimetre, Na+ - natrium, ng – nanogram, pg – pictogram, U – unit, Qs – significance value from the Q statistic; SE – standard error from the random effects model, TNF-α – Tumor Necrosis Factor-alpha, % – percentage, µmol – micromole
