# Supplementary material for: Dietary Tannins in Cultured Fish: Growth Promoter or Metabolic Burden? A Meta‐Analysis
Source: Aquac Nutr. 2026 Nov 15;2026:2460161. doi: 10.1155/anu/2460161 (PMC13770322; doi:10.1155/anu/2460161)
Supplement: Supplementary file 1 [file ANU-2026-2460161-s001.zip › Supplementary Materials/Table S5.docx]

| Table S5. Complete subgroup analysis of the effects of dietary tannin supplementation across all reported outcomes in cultured fish according to trophic classification. | | | | | | | | | | | | | | |
| --- | --- | --- | --- | --- | --- | --- | --- | --- | --- | --- | --- | --- | --- | --- |
| **Parameter** | **Subgroup** | **k** | ***g*** | **SE** | ***p*** | **Lower** | **Upper** | **FsN (R)** | ***I^2^*** | ***τ²*** | ***τ²′*** | **Q** | **Begg** | **Egger** |
| Specific growth rate (%g/day) | Omnivore | 224 | -1.57 | 0.16 | <0.001 | -1.89 | -1.25 | 125.55 | 81.28 | 4.01 | 5.48 | <0.001 | <0.001 | <0.001 |
| Specific growth rate (%g/day) | Carnivore | 62 | -0.47 | 0.30 | 0.12 | -1.05 | 0.12 | 1034.18 | 80.13 | 3.74 | 4.24 | <0.001 | <0.001 | <0.001 |
| Specific growth rate (%g/day) | Herbivore | 16 | -2.07 | 0.52 | <0.001 | -3.08 | -1.05 | -1.62 | 73.40 | 2.55 | 4.21 | <0.001 | <0.001 | <0.001 |
| Feed Conversion Ratio | Carnivore | 52 | 0.06 | 0.25 | 0.81 | -0.44 | 0.56 | 52294.31 | 74.69 | 2.27 | 2.44 | <0.001 | 0.65 | 0.60 |
| Feed Conversion Ratio | Omnivore | 214 | 1.54 | 0.16 | <0.001 | 1.23 | 1.86 | 131.07 | 80.22 | 3.67 | 4.93 | <0.001 | <0.001 | <0.001 |
| Feed Conversion Ratio | Herbivore | 21 | 1.45 | 0.35 | <0.001 | 0.77 | 2.14 | 17.13 | 63.52 | 1.46 | 2.70 | <0.001 | <0.001 | <0.001 |
| Protein efficiency ratio | Omnivore | 199 | -1.12 | 0.15 | <0.001 | -1.41 | -0.84 | 405.22 | 74.31 | 2.80 | 3.55 | <0.001 | <0.001 | <0.001 |
| Protein efficiency ratio | Carnivore | 23 | -1.10 | 0.38 | <0.001 | -1.84 | -0.36 | 49.70 | 73.40 | 2.13 | 3.44 | <0.001 | <0.001 | <0.001 |
| PER | Herbivore | 12 | -1.82 | 0.41 | <0.001 | -2.62 | -1.02 | 1.95 | 46.02 | 0.88 | 1.87 | 0.04 | <0.001 | <0.001 |
| ANPU (%) | Omnivore | 98 | -3.02 | 0.60 | <0.001 | -4.19 | -1.85 | -56.73 | 89.72 | 24.57 | 27.79 | <0.001 | <0.001 | <0.001 |
| ANPU (%) | Herbivore | 4 | -1.49 | 1.20 | 0.22 | -3.85 | 0.87 | 2.95 | 79.86 | 4.44 | 6.41 | <0.001 | 0.33 | 0.02 |
| Weight gain (%) | Omnivore | 227 | -1.54 | 0.22 | <0.001 | -1.97 | -1.11 | 139.19 | 84.77 | 7.20 | 10.01 | <0.001 | <0.001 | <0.001 |
| Weight gain (%) | Carnivore | 63 | -0.33 | 0.25 | 0.18 | -0.82 | 0.15 | 2129.98 | 76.71 | 2.69 | 2.69 | <0.001 | 0.05 | 0.88 |
| Weight gain (%) | Herbivore | 13 | -0.33 | 0.23 | 0.14 | -0.78 | 0.11 | 435.31 | 12.27 | 0.08 | 0.08 | 0.32 | <0.001 | <0.001 |
| Body crude protein content (%) | Carnivore | 41 | -0.32 | 0.22 | 0.14 | -0.74 | 0.11 | 1520.44 | 64.13 | 1.16 | 1.16 | <0.001 | <0.001 | <0.001 |
| Body crude protein content (%) | Omnivore | 177 | <0.001 | 0.17 | 0.98 | -0.33 | 0.32 | 42118817.40 | 77.98 | 3.33 | 3.33 | <0.001 | 0.56 | 0.87 |
| Body crude protein content (%) | Herbivore | 8 | -1.93 | 0.79 | 0.01 | -3.47 | -0.39 | 0.24 | 78.47 | 3.54 | 5.28 | <0.001 | <0.001 | <0.001 |
| Body crude lipid content (%) | Carnivore | 41 | -0.19 | 0.22 | 0.39 | -0.63 | 0.24 | 4272.49 | 64.79 | 1.21 | 1.21 | <0.001 | 0.12 | 0.73 |
| Body crude lipid content (%) | Omnivore | 161 | -1.21 | 0.20 | <0.001 | -1.59 | -0.83 | 260.60 | 80.58 | 4.49 | 5.84 | <0.001 | <0.001 | <0.001 |
| Body crude lipid content (%) | Herbivore | 8 | -1.66 | 0.80 | 0.04 | -3.23 | -0.09 | 3.15 | 79.65 | 4.04 | 4.04 | <0.001 | 0.10 | 0.11 |
| Body ash content (%) | Carnivore | 40 | 0.20 | 0.14 | 0.16 | -0.08 | 0.48 | 3752.89 | 22.17 | 0.18 | 0.18 | 0.11 | 0.02 | 0.38 |
| Body ash content (%) | Omnivore | 166 | -0.12 | 0.13 | 0.34 | -0.37 | 0.13 | 41989.29 | 67.82 | 1.68 | 1.89 | <0.001 | <0.001 | <0.001 |
| Body ash content (%) | Herbivore | 8 | -0.42 | 0.27 | 0.13 | -0.95 | 0.12 | 168.91 | <0.001 | <0.001 | <0.001 | 0.97 | 0.01 | 0.33 |
| Total carbohydrate (%) | Omnivore | 5 | -1.78 | 1.11 | 0.11 | -3.96 | 0.40 | 1.07 | 79.17 | 4.78 | 6.53 | <0.001 | 0.23 | 0.12 |
| Apparent protein digestibility (%) | Omnivore | 103 | -1.60 | 0.23 | <0.001 | -2.05 | -1.14 | 51.79 | 77.60 | 3.73 | 5.56 | <0.001 | <0.001 | <0.001 |
| Apparent protein digestibility (%) | Herbivore | 12 | -2.30 | 0.52 | <0.001 | -3.32 | -1.28 | -3.29 | 60.87 | 1.85 | 2.60 | <0.001 | <0.001 | <0.001 |
| Apparent protein digestibility (%) | Carnivore | 9 | -1.90 | 1.05 | 0.07 | -3.96 | 0.16 | 0.59 | 86.19 | 7.51 | 9.87 | <0.001 | 0.04 | <0.001 |
| Apparent lipid digestibility (%) | Omnivore | 35 | -1.08 | 0.24 | <0.001 | -1.56 | -0.61 | 79.38 | 48.46 | 2.07 | 3.69 | <0.001 | <0.001 | <0.001 |
| Apparent lipid digestibility (%) | Herbivore | 4 | -0.49 | 0.48 | 0.31 | -1.44 | 0.46 | 60.06 | 20.43 | 0.19 | 0.19 | 0.29 | 0.33 | 0.07 |
| Apparent lipid digestibility (%) | Carnivore | 6 | 0.40 | 0.84 | 0.63 | -1.25 | 2.06 | 136.56 | 80.42 | 3.37 | 3.37 | <0.001 | 1.00 | 0.77 |
| Apparent ash digestibility (%) | Omnivore | 19 | -1.63 | 0.65 | 0.01 | -2.92 | -0.35 | 8.40 | 81.67 | 5.43 | 7.24 | <0.001 | <0.001 | <0.001 |
| Apparent phosporous digestibility (%) | Omnivore | 11 | -1.81 | 0.73 | 0.01 | -3.25 | -0.38 | 1.84 | 77.56 | 4.05 | 4.05 | <0.001 | <0.001 | <0.001 |
| Apparent calcium digestibility (%) | Omnivore | 7 | -0.34 | 0.63 | 0.59 | -1.57 | 0.89 | 227.45 | 67.83 | 1.87 | 1.87 | <0.001 | 1.00 | <0.001 |
| Apparent manganese digestibility (%) | Omnivore | 7 | -1.14 | 0.71 | 0.11 | -2.53 | 0.26 | 13.79 | 71.93 | 2.52 | 2.52 | <0.001 | 0.03 | <0.001 |
| Apparent copper digestibility (%) | Omnivore | 7 | -0.62 | 0.72 | 0.39 | -2.03 | 0.79 | 63.68 | 73.42 | 2.64 | 2.64 | <0.001 | 0.38 | 0.55 |
| Apparent iron digestibility (%) | Omnivore | 7 | -1.46 | 0.84 | 0.08 | -3.10 | 0.17 | 5.55 | 76.83 | 3.68 | 3.68 | <0.001 | 0.01 | <0.001 |
| Whole body phosphorus content (mg/g) | Omnivore | 7 | -1.40 | 1.15 | 0.22 | -3.65 | 0.85 | 6.70 | 84.28 | 7.66 | 8.53 | <0.001 | 0.14 | 0.17 |
| Whole body calcium content (mg/g) | Omnivore | 7 | -0.68 | 1.37 | 0.62 | -3.36 | 2.00 | 51.07 | 84.41 | 10.62 | 10.62 | <0.001 | 1.00 | <0.001 |
| Whole body manganese content (µg/g) | Omnivore | 7 | -3.77 | 2.11 | 0.07 | -7.90 | 0.37 | -5.11 | 89.97 | 27.13 | 27.13 | <0.001 | <0.001 | <0.001 |
| Whole body copper content (µg/g) | Omnivore | 7 | -5.87 | 1.20 | <0.001 | -8.21 | -3.52 | -6.22 | 64.14 | 5.79 | 8.60 | 0.01 | <0.001 | <0.001 |
| Whole body iron content (µg/g) | Omnivore | 7 | -2.58 | 0.88 | <0.001 | -4.30 | -0.86 | -2.96 | 74.08 | 3.80 | 3.80 | <0.001 | <0.001 | <0.001 |
| Faecal phophorus concentration (mg/g) | Omnivore | 7 | 4.75 | 1.23 | <0.001 | 2.34 | 7.17 | -5.81 | 76.39 | 7.05 | 8.59 | <0.001 | <0.001 | <0.001 |
| Survival Rate (%) | Omnivore | 61 | -0.22 | 0.14 | 0.11 | -0.50 | 0.05 | 4670.49 | 42.30 | 0.49 | 0.49 | <0.001 | <0.001 | <0.001 |
| Survival Rate (%) | Carnivore | 50 | 0.18 | 0.11 | 0.09 | -0.03 | 0.40 | 5594.69 | <0.001 | <0.001 | <0.001 | 0.48 | 0.34 | 0.01 |
| Survival Rate (%) | Herbivore | 9 | 0.20 | 0.24 | 0.40 | -0.26 | 0.67 | 848.76 | <0.001 | <0.001 | <0.001 | 0.99 | 0.01 | 0.26 |
| Average daily feed intake (%g/day) | Carnivore | 6 | -4.53 | 3.82 | 0.24 | -12.02 | 2.95 | -4.88 | 91.62 | 69.49 | 78.73 | <0.001 | 0.14 | 0.59 |
| Feed intake (g/fish/day) | Omnivore | 45 | -0.76 | 0.27 | <0.001 | -1.28 | -0.23 | 256.30 | 69.81 | 2.10 | 2.10 | <0.001 | <0.001 | <0.001 |
| Feed intake (g/fish/day) | Carnivore | 35 | -0.82 | 0.28 | <0.001 | -1.37 | -0.26 | 166.83 | 70.90 | 1.81 | 2.90 | <0.001 | <0.001 | <0.001 |
| Feed intake (g/fish/day) | Herbivore | 14 | -1.35 | 0.55 | 0.01 | -2.43 | -0.27 | 15.41 | 75.63 | 3.07 | 3.77 | <0.001 | <0.001 | <0.001 |
| Condition factor (g/cm3) | Omnivore | 8 | 1.56 | 0.63 | 0.01 | 0.33 | 2.79 | 4.63 | 85.32 | 1.84 | 2.40 | <0.001 | 0.55 | <0.001 |
| Condition factor (g/cm3) | Carnivore | 31 | -0.07 | 0.15 | 0.65 | -0.37 | 0.23 | 25265.76 | 14.27 | 0.10 | 0.10 | 0.24 | 0.21 | 0.13 |
| Hepatosomatic Index (%) | Omnivore | 49 | -0.41 | 0.18 | 0.02 | -0.76 | -0.06 | 1065.24 | 49.01 | 0.73 | 0.73 | <0.001 | <0.001 | 0.04 |
| Hepatosomatic Index (%) | Carnivore | 35 | -0.41 | 0.17 | 0.01 | -0.74 | -0.08 | 756.61 | 32.39 | 0.31 | 0.31 | 0.04 | <0.001 | <0.001 |
| Viscerosomatic index (%) | Omnivore | 9 | 0.15 | 0.45 | 0.74 | -0.74 | 1.03 | 1567.06 | 53.72 | 0.94 | 1.73 | 0.03 | 0.48 | 0.01 |
| Viscerosomatic index (%) | Carnivore | 36 | -0.18 | 0.15 | 0.24 | -0.48 | 0.12 | 4238.71 | 23.44 | 0.20 | 0.20 | 0.11 | <0.001 | <0.001 |
| Viscerosomatic index (%) | Herbivore | 3 | -5.20 | 0.86 | <0.001 | -6.89 | -3.51 | -2.57 | <0.001 | <0.001 | <0.001 | 0.66 | 0.33 | 0.36 |
| Intestinosomatic index (%) | Omnivore | 16 | -0.16 | 0.21 | 0.45 | -0.58 | 0.26 | 2385.86 | <0.001 | <0.001 | <0.001 | 0.55 | 0.36 | 0.02 |
| Intestinosomatic index (%) | Carnivore | 11 | -0.18 | 0.22 | 0.41 | -0.62 | 0.26 | 1249.81 | <0.001 | <0.001 | <0.001 | 0.96 | 0.38 | 0.99 |
| Intestinosomatic index (%) | Herbivore | 3 | -0.14 | 0.41 | 0.74 | -0.94 | 0.67 | 620.97 | <0.001 | <0.001 | <0.001 | 0.95 | 0.33 | 0.76 |
| Intestinosomatic index (%) | Carnivore | 6 | 0.11 | 0.58 | 0.84 | -1.03 | 1.26 | 1746.76 | 59.73 | 1.20 | 1.20 | 0.03 | 1.00 | 0.05 |
| IPF (%) | Carnivore | 12 | -1.54 | 0.40 | <0.001 | -2.33 | -0.75 | 7.44 | 58.65 | 1.11 | 1.74 | 0.01 | <0.001 | <0.001 |
| Total Antioxidant Capacity (U/mL) | Carnivore | 38 | 1.57 | 0.28 | <0.001 | 1.03 | 2.11 | 21.48 | 69.35 | 1.88 | 2.84 | <0.001 | <0.001 | <0.001 |
| Total Antioxidant Capacity (U/mL) | Omnivore | 10 | -0.68 | 0.31 | 0.03 | -1.28 | -0.07 | 74.08 | 18.19 | 0.17 | 0.17 | 0.28 | <0.001 | <0.001 |
| Total Antioxidant Capacity (U/mL) | Herbivore | 3 | -2.93 | 1.15 | 0.01 | -5.18 | -0.68 | -1.66 | 59.99 | 2.33 | 4.73 | 0.08 | 0.33 | 0.03 |
| Total Cholesterol (mmol/L) | Omnivore | 24 | -1.54 | 0.25 | <0.001 | -2.04 | -1.04 | 14.99 | 32.08 | 0.48 | 1.22 | 0.07 | <0.001 | <0.001 |
| Total Cholesterol (mmol/L) | Carnivore | 27 | -0.61 | 0.22 | <0.001 | -1.03 | -0.19 | 251.67 | 49.39 | 0.61 | 0.61 | <0.001 | <0.001 | <0.001 |
| Total Cholesterol (mmol/L) | Herbivore | 3 | 8.84 | 7.08 | 0.21 | -5.04 | 22.71 | -2.85 | 94.65 | 140.51 | 140.51 | <0.001 | 1.00 | <0.001 |
| Triacylglycerol (mmol/L) | Carnivore | 21 | -0.88 | 0.21 | <0.001 | -1.30 | -0.47 | 82.87 | 35.52 | 0.33 | 0.33 | 0.05 | <0.001 | <0.001 |
| Glucose (mmol/L) | Carnivore | 28 | -0.01 | 0.22 | 0.95 | -0.44 | 0.42 | 498804.10 | 54.50 | 0.71 | 0.71 | <0.001 | 0.45 | <0.001 |
| Glucose (mmol/L) | Omnivore | 19 | -0.46 | 0.38 | 0.23 | -1.22 | 0.29 | 321.12 | 83.63 | 1.89 | 2.30 | <0.001 | 0.11 | 0.01 |
| Blood Urea Nitrogen (mmol/L) | Omnivore | 8 | 1.38 | 0.48 | <0.001 | 0.44 | 2.32 | 8.08 | 86.07 | 1.49 | 1.49 | <0.001 | 0.18 | 0.74 |
| Blood Urea Nitrogen (mmol/L) | Carnivore | 13 | 0.13 | 0.20 | 0.52 | -0.26 | 0.52 | 3000.60 | <0.001 | <0.001 | <0.001 | 0.98 | 0.23 | 0.41 |
| Blood Urea Nitrogen (mmol/L) | Herbivore | 4 | -21.66 | 2.78 | <0.001 | -27.10 | -16.22 | -3.97 | <0.001 | <0.001 | <0.001 | 0.56 | 0.06 | 0.15 |
| Diamine oxide (U/L) | Carnivore | 13 | -0.26 | 0.51 | 0.62 | -1.26 | 0.75 | 741.27 | 78.40 | 2.58 | 2.58 | <0.001 | 0.77 | 0.18 |
| Lactate (µmol/L) | Carnivore | 8 | -0.33 | 0.26 | 0.21 | -0.84 | 0.18 | 274.42 | 1.73 | 0.01 | 0.01 | 0.42 | 0.06 | 0.06 |
| Lipopolysaccharide (pg/mL) | Carnivore | 11 | -0.69 | 0.53 | 0.19 | -1.73 | 0.35 | 77.02 | 75.27 | 2.22 | 3.09 | <0.001 | 0.45 | 0.03 |
| Superoxide dismutase (U/mL) | Omnivore | 32 | 0.07 | 0.23 | 0.77 | -0.39 | 0.53 | 26040.77 | 66.94 | 1.01 | 1.01 | <0.001 | 0.38 | 0.71 |
| Superoxide dismutase (U/mL) | Carnivore | 47 | 1.35 | 0.44 | <0.001 | 0.49 | 2.22 | 51.37 | 85.28 | 6.66 | 7.36 | <0.001 | 0.01 | <0.001 |
| Superoxide dismutase (U/mL) | Herbivore | 3 | 4.57 | 3.11 | 0.14 | -1.53 | 10.68 | -2.45 | 89.07 | 25.48 | 25.48 | <0.001 | 0.33 | <0.001 |
| Catalase activity (kU/L) | Omnivore | 30 | 0.41 | 0.19 | 0.03 | 0.03 | 0.78 | 661.14 | 51.50 | 0.49 | 0.98 | <0.001 | <0.001 | <0.001 |
| Catalase activity (kU/L) | Carnivore | 45 | 1.48 | 0.32 | <0.001 | 0.86 | 2.11 | 33.49 | 77.63 | 3.26 | 4.31 | <0.001 | <0.001 | <0.001 |
| Glutathione S-transferase (pmol/min/mg protein) | Omnivore | 5 | -0.22 | 0.22 | 0.32 | -0.66 | 0.22 | 376.55 | <0.001 | <0.001 | <0.001 | 0.99 | 0.02 | 0.65 |
| Glutathione S-transferase (pmol/min/mg protein) | Carnivore | 9 | 1.11 | 0.55 | 0.05 | 0.02 | 2.19 | 19.22 | 67.52 | 1.73 | 1.73 | <0.001 | 0.18 | <0.001 |
| Glutathione peroxidase (U/mL) | Omnivore | 9 | 0.11 | 0.29 | 0.69 | -0.45 | 0.68 | 2676.08 | 3.47 | 0.03 | 0.03 | 0.41 | 0.60 | 0.07 |
| Glutathione peroxidase (U/mL) | Carnivore | 16 | 0.29 | 0.18 | 0.11 | -0.07 | 0.64 | 733.09 | <0.001 | <0.001 | <0.001 | 0.99 | <0.001 | 0.04 |
| Malondialdehyde (U/mL) | Carnivore | 34 | -0.12 | 0.33 | 0.72 | -0.77 | 0.53 | 8823.79 | 76.23 | 2.45 | 4.26 | <0.001 | 0.66 | <0.001 |
| Malondialdehyde (U/mL) | Omnivore | 20 | 0.09 | 0.25 | 0.73 | -0.41 | 0.58 | 10146.40 | 54.34 | 0.59 | 0.59 | <0.001 | 0.08 | <0.001 |
| TBARS (nmol/g) | Omnivore | 9 | 0.32 | 0.39 | 0.40 | -0.43 | 1.08 | 321.18 | 67.81 | 0.83 | 0.83 | <0.001 | 0.01 | 0.37 |
| Lysozyme activity (U/mL) | Omnivore | 14 | 1.21 | 0.51 | 0.02 | 0.22 | 2.20 | 22.80 | 80.84 | 2.85 | 2.85 | <0.001 | 0.59 | 0.96 |
| Lysozyme activity (U/mL) | Carnivore | 26 | 1.49 | 0.47 | <0.001 | 0.58 | 2.41 | 18.71 | 81.76 | 4.35 | 5.23 | <0.001 | <0.001 | <0.001 |
| Peroxidase activity (OD 450 nm) | Omnivore | 5 | -1.31 | 0.34 | <0.001 | -1.98 | -0.65 | 6.14 | 44.44 | 0.25 | 0.25 | 0.13 | 0.02 | 0.01 |
| Plasma bactericidal activity | Omnivore | 5 | 0.93 | 0.49 | 0.06 | -0.02 | 1.89 | 17.10 | 74.32 | 0.88 | 0.88 | <0.001 | 0.08 | 0.02 |
| Nitro blue tetrazolium (OD 540 nm) | Omnivore | 10 | 0.08 | 0.46 | 0.87 | -0.84 | 0.99 | 6805.42 | 76.36 | 1.47 | 2.52 | <0.001 | 0.31 | <0.001 |
| Amylase (U/mg protein) | Carnivore | 42 | -0.48 | 0.23 | 0.04 | -0.94 | -0.02 | 660.32 | 68.11 | 1.49 | 1.49 | <0.001 | 0.01 | 0.10 |
| Amylase (U/mg protein) | Omnivore | 42 | -1.65 | 0.40 | <0.001 | -2.44 | -0.86 | 17.33 | 81.48 | 4.76 | 5.33 | <0.001 | <0.001 | <0.001 |
| Amylase (U/mg protein) | Herbivore | 10 | 1.14 | 1.12 | 0.31 | -1.06 | 3.34 | 19.65 | 87.90 | 9.04 | 9.04 | <0.001 | 0.47 | <0.001 |
| Lipase (U/mg protein) | Carnivore | 42 | 0.53 | 0.30 | 0.08 | -0.07 | 1.13 | 531.96 | 77.84 | 2.72 | 2.72 | <0.001 | <0.001 | <0.001 |
| Lipase (U/mg protein) | Omnivore | 35 | -1.82 | 0.39 | <0.001 | -2.58 | -1.06 | 5.55 | 78.86 | 3.77 | 5.28 | <0.001 | <0.001 | <0.001 |
| Lipase (U/mg protein) | Herbivore | 10 | -2.00 | 0.52 | <0.001 | -3.01 | -0.99 | -0.37 | 63.43 | 1.60 | 2.11 | <0.001 | <0.001 | <0.001 |
| Trypsin (U/mg protein) | Carnivore | 26 | -1.36 | 0.43 | <0.001 | -2.20 | -0.52 | 27.98 | 81.56 | 3.61 | 4.50 | <0.001 | <0.001 | <0.001 |
| Trypsin (U/mg protein) | Herbivore | 4 | 5.18 | 0.76 | <0.001 | 3.70 | 6.66 | -3.43 | <0.001 | <0.001 | 1.12 | 0.40 | 0.06 | 0.08 |
| Creatine kinase (U/mg protein) | Carnivore | 5 | -0.43 | 1.01 | 0.67 | -2.41 | 1.56 | 99.84 | 81.93 | 3.53 | 6.54 | <0.001 | 0.79 | <0.001 |
| Na+, K+- ATPase (U/mg protein) | Carnivore | 5 | -5.41 | 2.33 | 0.02 | -9.96 | -0.85 | -4.34 | 86.32 | 15.68 | 24.62 | <0.001 | 0.02 | <0.001 |
| Protease (U/mg protein) | Carnivore | 13 | -1.10 | 0.95 | 0.24 | -2.96 | 0.76 | 28.07 | 84.46 | 7.75 | 7.75 | <0.001 | 0.25 | 0.87 |
| Protease (U/mg protein) | Omnivore | 34 | -0.71 | 0.34 | 0.04 | -1.38 | -0.03 | 228.48 | 76.28 | 2.86 | 4.06 | <0.001 | <0.001 | <0.001 |
| Protease (U/mg protein) | Herbivore | 6 | -2.08 | 0.79 | 0.01 | -3.63 | -0.53 | -0.67 | 69.70 | 2.41 | 3.82 | 0.01 | <0.001 | <0.001 |
| Pepsin (U/mg protein) | Carnivore | 11 | -2.38 | 0.59 | <0.001 | -3.54 | -1.21 | -3.51 | 66.09 | 2.33 | 3.16 | <0.001 | <0.001 | <0.001 |
| Hematocrit (%) | Omnivore | 22 | 0.56 | 0.26 | 0.03 | 0.05 | 1.08 | 244.82 | 79.83 | 1.12 | 1.12 | <0.001 | 0.02 | 0.41 |
| Creatinine (mg/dL) | Omnivore | 4 | 8.15 | 2.05 | <0.001 | 4.15 | 12.16 | -3.77 | 38.42 | 6.22 | 14.23 | 0.18 | 0.08 | 0.03 |
| Total bilirubin (mg/dL) | Omnivore | 8 | 0.14 | 0.46 | 0.76 | -0.77 | 1.05 | 1539.52 | 44.72 | 0.74 | 1.30 | 0.08 | 0.90 | 0.18 |
| Total protein (g/dL) | Omnivore | 41 | -0.53 | 0.25 | 0.04 | -1.02 | -0.04 | 521.15 | 80.78 | 1.90 | 1.90 | <0.001 | 0.54 | 0.75 |
| Total protein (g/dL) | Carnivore | 11 | 1.17 | 0.59 | 0.05 | 0.02 | 2.32 | 19.92 | 72.24 | 2.55 | 2.55 | <0.001 | 0.02 | <0.001 |
| Total protein (g/dL) | Herbivore | 4 | 2.89 | 2.02 | 0.15 | -1.07 | 6.84 | -2.16 | 91.00 | 14.44 | 14.44 | <0.001 | 0.25 | 0.04 |
| Triglyceride (g/dL) | Omnivore | 11 | -1.27 | 0.41 | <0.001 | -2.07 | -0.47 | 15.19 | 49.95 | 0.91 | 1.26 | 0.03 | <0.001 | <0.001 |
| Triglyceride (g/dL) | Carnivore | 9 | -0.57 | 0.74 | 0.44 | -2.02 | 0.88 | 97.94 | 74.79 | 3.05 | 4.47 | <0.001 | 0.76 | <0.001 |
| Triglyceride (g/dL) | Herbivore | 3 | 3.06 | 3.75 | 0.42 | -4.30 | 10.42 | -1.77 | 94.35 | 39.78 | 39.78 | <0.001 | 0.33 | <0.001 |
| Phosphate (mmol/L) | Omnivore | 5 | -0.55 | 0.37 | 0.14 | -1.28 | 0.18 | 58.02 | <0.001 | <0.001 | <0.001 | 0.98 | 0.02 | 0.51 |
| Alkaline phosphatase (IU/L) | Omnivore | 18 | 0.01 | 0.26 | 0.98 | -0.51 | 0.52 | 2586142.88 | 56.95 | 0.58 | 1.08 | <0.001 | <0.001 | <0.001 |
| Alkaline phosphatase (IU/L) | Carnivore | 18 | 1.14 | 0.36 | <0.001 | 0.43 | 1.86 | 34.76 | 65.31 | 1.42 | 1.75 | <0.001 | <0.001 | <0.001 |
| Alanine aminotransferase (IU/L) | Carnivore | 29 | 0.55 | 0.33 | 0.09 | -0.10 | 1.19 | 340.70 | 75.03 | 2.20 | 2.74 | <0.001 | 0.03 | <0.001 |
| Alanine aminotransferase (IU/L) | Omnivore | 36 | 0.15 | 0.19 | 0.43 | -0.23 | 0.53 | 5961.88 | 59.41 | 0.65 | 0.65 | <0.001 | 0.51 | 0.58 |
| Alanine aminotransferase (IU/L) | Herbivore | 3 | 3.04 | 2.06 | 0.14 | -1.01 | 7.09 | -1.75 | 85.85 | 10.40 | 16.97 | <0.001 | 0.33 | <0.001 |
| Aspartate aminotransferase (IU/L) | Carnivore | 29 | 0.41 | 0.32 | 0.20 | -0.22 | 1.05 | 627.78 | 74.18 | 2.10 | 2.10 | <0.001 | 0.09 | 0.08 |
| Aspartate aminotransferase (IU/L) | Omnivore | 31 | 0.67 | 0.29 | 0.02 | 0.10 | 1.23 | 237.79 | 76.41 | 1.50 | 1.78 | <0.001 | <0.001 | <0.001 |
| Aspartate aminotransferase (IU/L) | Herbivore | 3 | -1.79 | 2.42 | 0.46 | -6.53 | 2.96 | 0.60 | 89.80 | 15.48 | 20.40 | <0.001 | 1.00 | 0.23 |
| Lactate dehydrogenase (U/mg protein) | Omnivore | 15 | 0.02 | 0.24 | 0.94 | -0.45 | 0.48 | 158291.58 | 47.92 | 0.34 | 0.76 | 0.02 | 0.05 | 0.01 |
| Malate dehydrogenase (U/mg protein) | Omnivore | 3 | 4.89 | 1.92 | 0.01 | 1.13 | 8.66 | -2.52 | 72.53 | 7.89 | 13.49 | 0.03 | 0.33 | 0.01 |
| Red blood cell (x1012L) | Omnivore | 29 | 0.15 | 0.26 | 0.57 | -0.36 | 0.65 | 5238.63 | 78.45 | 1.27 | 1.27 | <0.001 | 0.47 | 0.02 |
| White Blood Cells (x 10¹²/L) | Omnivore | 29 | 0.88 | 0.33 | 0.01 | 0.23 | 1.54 | 113.52 | 86.60 | 2.63 | 2.63 | <0.001 | 0.17 | 0.12 |
| Haemoglobin (g/dL) | Carnivore | 4 | 3.83 | 0.88 | <0.001 | 2.11 | 5.55 | -2.95 | 49.03 | 1.46 | 1.46 | 0.12 | 0.08 | 0.02 |
| Haemoglobin (g/dL) | Omnivore | 37 | -0.07 | 0.24 | 0.78 | -0.54 | 0.41 | 32318.18 | 77.66 | 1.39 | 2.02 | <0.001 | 0.03 | <0.001 |
| Platelet (%) | Omnivore | 9 | 0.46 | 0.29 | 0.11 | -0.10 | 1.02 | 153.78 | 0.31 | <0.001 | <0.001 | 0.43 | <0.001 | 0.01 |
| Packed Cell Volume (%) | Carnivore | 4 | -4.06 | 0.62 | <0.001 | -5.28 | -2.83 | -3.07 | <0.001 | <0.001 | <0.001 | 0.72 | 0.08 | 0.25 |
| Packed Cell Volume (%) | Omnivore | 10 | -0.36 | 0.67 | 0.60 | -1.67 | 0.96 | 294.39 | 72.63 | 2.98 | 2.98 | <0.001 | 0.86 | 0.09 |
| MCV (fL) | Omnivore | 31 | -0.18 | 0.27 | 0.49 | -0.71 | 0.34 | 3508.98 | 81.12 | 1.58 | 2.85 | <0.001 | 0.06 | 0.43 |
| MCH | Omnivore | 31 | 0.19 | 0.31 | 0.55 | -0.42 | 0.80 | 3378.03 | 85.15 | 2.30 | 2.76 | <0.001 | 0.05 | 0.45 |
| MCHC | Omnivore | 31 | 0.39 | 0.20 | 0.06 | -0.01 | 0.78 | 761.92 | 69.13 | 0.77 | 1.04 | <0.001 | 0.92 | 0.60 |
| Neutrophils | Omnivore | 13 | 0.18 | 0.24 | 0.44 | -0.28 | 0.64 | 1493.14 | <0.001 | <0.001 | <0.001 | 0.57 | 0.95 | 0.01 |
| Lymphocytes | Omnivore | 17 | -0.07 | 0.28 | 0.81 | -0.61 | 0.48 | 15175.28 | 39.92 | 0.51 | 0.51 | 0.05 | 0.97 | 0.44 |
| Basophils | Omnivore | 14 | 0.02 | 0.22 | 0.94 | -0.42 | 0.45 | 180561.04 | <0.001 | <0.001 | <0.001 | 0.99 | 0.03 | 0.64 |
| Monocytes | Omnivore | 17 | 0.33 | 0.21 | 0.11 | -0.07 | 0.73 | 585.46 | <0.001 | <0.001 | <0.001 | 0.76 | 0.03 | 0.06 |
| Erythrocytes | Carnivore | 4 | 0.52 | 0.43 | 0.22 | -0.32 | 1.35 | 53.34 | 23.95 | 0.17 | 0.17 | 0.27 | 0.33 | 0.06 |
| Erythrocytes | Omnivore | 11 | 0.05 | 0.68 | 0.94 | -1.27 | 1.38 | 15479.02 | 74.38 | 2.90 | 4.46 | <0.001 | 0.48 | 0.06 |
| Thrombocytes | Omnivore | 3 | 0.45 | 0.42 | 0.28 | -0.36 | 1.27 | 53.38 | <0.001 | <0.001 | <0.001 | 0.69 | 0.33 | 0.40 |
| Leukocytes | Carnivore | 4 | 2.66 | 0.49 | <0.001 | 1.70 | 3.62 | -1.83 | <0.001 | <0.001 | <0.001 | 0.86 | 0.08 | 0.39 |
| Leukocytes | Omnivore | 8 | 2.76 | 0.94 | <0.001 | 0.91 | 4.61 | -3.97 | 81.37 | 4.87 | 6.41 | <0.001 | 0.01 | <0.001 |
| Eosinophils | Omnivore | 11 | -0.34 | 0.25 | 0.18 | -0.84 | 0.16 | 358.07 | <0.001 | <0.001 | <0.001 | 0.73 | 0.03 | 0.01 |
| Villus length (cm) | Omnivore | 24 | 0.60 | 0.42 | 0.15 | -0.23 | 1.43 | 230.67 | 83.66 | 3.21 | 3.63 | <0.001 | 0.06 | 0.53 |
| Villus length (cm) | Herbivore | 8 | -1.63 | 0.39 | <0.001 | -2.40 | -0.86 | 3.52 | 20.97 | 0.26 | 0.89 | 0.26 | <0.001 | <0.001 |
| Villus length (cm) | Carnivore | 13 | 0.10 | 0.49 | 0.84 | -0.85 | 1.05 | 5247.09 | 77.10 | 2.33 | 2.33 | <0.001 | 0.86 | 0.25 |
| Villus width (cm) | Omnivore | 21 | -1.39 | 0.48 | <0.001 | -2.32 | -0.46 | 20.74 | 82.32 | 3.53 | 4.00 | <0.001 | 0.53 | <0.001 |
| Villus width (cm) | Herbivore | 8 | 0.05 | 0.29 | 0.86 | -0.52 | 0.62 | 12484.78 | <0.001 | <0.001 | <0.001 | 1.00 | 0.01 | 0.84 |
| Villus width (cm) | Carnivore | 13 | 0.82 | 0.50 | 0.10 | -0.16 | 1.80 | 61.46 | 76.90 | 2.42 | 2.42 | <0.001 | 0.08 | 0.04 |
| Villi density (mm2) | Omnivore | 10 | 1.32 | 0.54 | 0.01 | 0.27 | 2.37 | 11.98 | 87.55 | 2.47 | 2.47 | <0.001 | 0.03 | <0.001 |
| Muscular thickness (cm) | Carnivore | 9 | 0.11 | 0.24 | 0.65 | -0.36 | 0.58 | 2927.46 | <0.001 | <0.001 | <0.001 | 0.85 | 0.36 | 0.78 |
| Goblet cell number | Omnivore | 3 | 2.15 | 1.23 | 0.08 | -0.26 | 4.55 | -0.50 | 91.18 | 4.09 | 4.09 | <0.001 | 0.33 | <0.001 |
| Goblet cell number) | Herbivore | 8 | 1.31 | 0.38 | <0.001 | 0.57 | 2.05 | 9.92 | 20.94 | 0.24 | 0.56 | 0.26 | <0.001 | <0.001 |
| Goblet cell number | Carnivore | 3 | -0.66 | 0.42 | 0.12 | -1.49 | 0.16 | 23.10 | <0.001 | <0.001 | <0.001 | 0.70 | 0.33 | 0.40 |
| Albumin (g/L) | Omnivore | 23 | 0.08 | 0.22 | 0.70 | -0.34 | 0.51 | 12234.77 | 49.51 | 0.46 | 0.87 | <0.001 | 0.07 | 0.03 |
| Albumin (g/L) | Carnivore | 19 | -0.12 | 0.33 | 0.71 | -0.77 | 0.53 | 4753.86 | 66.50 | 1.34 | 1.34 | <0.001 | 0.53 | 0.03 |
| Albumin (g/L) | Herbivore | 4 | 0.19 | 1.20 | 0.87 | -2.15 | 2.54 | 417.40 | 84.41 | 4.54 | 4.54 | <0.001 | 0.70 | <0.001 |
| Globulin (g/L) | Omnivore | 16 | -0.56 | 0.50 | 0.26 | -1.55 | 0.42 | 177.36 | 70.91 | 2.52 | 3.28 | <0.001 | 0.05 | <0.001 |
| Globulin (g/L) | Carnivore | 15 | 0.42 | 0.19 | 0.03 | 0.05 | 0.80 | 309.69 | <0.001 | <0.001 | <0.001 | 0.85 | <0.001 | 0.16 |
| Globulin (g/L) | Herbivore | 4 | 0.10 | 2.32 | 0.97 | -4.44 | 4.64 | 1517.30 | 92.67 | 19.68 | 19.68 | <0.001 | 0.70 | 0.51 |
| HDLC (mmol/L) | Omnivore | 5 | -0.91 | 0.48 | 0.06 | -1.84 | 0.03 | 18.37 | 29.00 | 0.33 | 0.42 | 0.23 | 0.08 | 0.04 |
| HDLC (mmol/L) | Carnivore | 18 | 0.36 | 0.25 | 0.15 | -0.13 | 0.86 | 506.55 | 49.05 | 0.55 | 0.55 | 0.01 | 0.02 | <0.001 |
| HDLC (mmol/L) | Herbivore | 3 | -7.20 | 1.13 | <0.001 | -9.43 | -4.98 | -2.78 | <0.001 | <0.001 | 1.88 | 0.49 | 0.33 | 0.23 |
| LDLC (mmol/L) | Omnivore | 5 | -0.46 | 0.55 | 0.40 | -1.53 | 0.61 | 86.27 | 47.38 | 0.71 | 0.71 | 0.11 | 0.48 | 0.02 |
| LDLC (mmol/L) | Carnivore | 18 | 0.49 | 0.26 | 0.06 | -0.01 | 0.99 | 271.94 | 50.12 | 0.58 | 0.86 | 0.01 | <0.001 | <0.001 |
| LDLC (mmol/L) | Herbivore | 3 | -5.49 | 4.47 | 0.22 | -14.26 | 3.28 | -2.62 | 94.10 | 55.75 | 55.75 | <0.001 | 0.33 | <0.001 |
| Insulin (pg/mL) | Carnivore | 3 | 0.98 | 0.91 | 0.28 | -0.79 | 2.75 | 8.98 | 70.50 | 1.72 | 1.72 | 0.03 | 0.33 | 0.01 |
| Glucagon (ng/mL) | Carnivore | 3 | -0.16 | 0.43 | 0.70 | -1.00 | 0.68 | 425.92 | <0.001 | <0.001 | <0.001 | 0.88 | 1.00 | 0.84 |
| TNF-α (ng/L) | Carnivore | 8 | -1.62 | 0.29 | <0.001 | -2.19 | -1.05 | 3.72 | <0.001 | <0.001 | <0.001 | 0.95 | <0.001 | 0.14 |
| Nrf2 (ng/L) | Herbivore | 3 | 2.35 | 0.53 | <0.001 | 1.31 | 3.40 | -0.92 | <0.001 | <0.001 | <0.001 | 0.92 | 0.33 | 0.68 |
| Keap1 | Herbivore | 3 | 6.77 | 1.28 | <0.001 | 4.26 | 9.28 | -2.75 | 26.72 | 1.37 | 5.76 | 0.26 | 0.33 | 0.10 |
| IL-6 (ng/L) | Carnivore | 8 | -0.93 | 0.67 | 0.17 | -2.24 | 0.39 | 27.91 | 79.50 | 2.74 | 3.31 | <0.001 | 0.26 | <0.001 |
| IL-8 (ng/L) | Carnivore | 8 | 0.69 | 0.26 | 0.01 | 0.18 | 1.20 | 57.20 | <0.001 | <0.001 | <0.001 | 0.83 | <0.001 | 0.07 |
| IL-8 (ng/L) | Herbivore | 3 | 2.01 | 0.50 | <0.001 | 1.02 | 2.99 | -0.13 | <0.001 | <0.001 | <0.001 | 0.89 | 0.33 | 0.62 |
| IL-10 (ng/L) | Carnivore | 5 | 0.36 | 0.32 | 0.26 | -0.27 | 0.99 | 141.49 | <0.001 | <0.001 | <0.001 | 0.90 | 0.02 | 0.33 |
| IL-10 (ng/L) | Herbivore | 3 | 3.26 | 0.93 | <0.001 | 1.45 | 5.08 | -1.92 | 50.03 | 1.27 | 1.27 | 0.14 | 0.33 | 0.05 |
| Immune globulin (µg/mg) | Omnivore | 7 | -0.25 | 0.29 | 0.38 | -0.82 | 0.32 | 412.89 | <0.001 | <0.001 | <0.001 | 1.00 | 0.76 | 0.76 |
| Immune globulin (µg/mg) | Carnivore | 8 | 1.53 | 0.30 | <0.001 | 0.93 | 2.12 | 5.18 | 2.97 | 0.02 | 0.34 | 0.41 | <0.001 | 0.01 |

*g* – Hedges’ *g*; *p* – significance value for the pooled effect size, Q – significance value from Cochran’s Q test for heterogeneity, Begg – significance value from Begg’s test, Egger – significance value from Egger’s regression test, FSN – fail state number, cm – centimetre, cm^3^ – cubic centimetre, dL – decilitre, fL – femtoliter, FSN – fail state number, HDLC – high density lipoprotein cholesterol, kU – kilounit, µg – microgram, g – gram, IL – interleukin, *I²* – heterogeneity, *τ²* – estimated between-study variance from the original random-effects model; *τ²′* – estimated between-study variance from the corresponding adjusted/prime random-effects model; K+ - Kalium, k – number of studies, IU – international unit, L – litre, mg – milligram, LDLC – low density lipoprotein cholesterol, mL – mililiter, mmol – milimol, mm^2^ – square millimetre, Na+ - natrium, ng – nanogram, pg – pictogram, U – unit, Qs – significance value from the Q statistic; SE – standard error from the random effects model, TNF-α – Tumor Necrosis Factor-alpha, % – percentage, µmol – micromole
