# Supplementary material for: Dietary Tannins in Cultured Fish: Growth Promoter or Metabolic Burden? A Meta‐Analysis
Source: Aquac Nutr. 2026 Nov 15;2026:2460161. doi: 10.1155/anu/2460161 (PMC13770322; doi:10.1155/anu/2460161)
Supplement: Supplementary file 1 [file ANU-2026-2460161-s001.zip › Supplementary Materials/Table S7.docx]

| **Table S7.** Complete subgroup analysis of the effects of dietary tannin supplementation across all reported outcomes in cultured fish according to fish species. | | | | | | | | | | | | | | | |
| --- | --- | --- | --- | --- | --- | --- | --- | --- | --- | --- | --- | --- | --- | --- | --- |
| **Parameter** | **Category** | **Subgroup** | **k** | ***g*** | **SE** | ***p*** | **Lower** | **Upper** | **FsN (R)** | ***I^2^*** | ***τ²*** | ***τ²′*** | **Q** | **Begg** | **Egger** |
| Specific growth rate (%g/day) | Species | *Sparus aurata* | 3 | -0.86 | 0.80 | 0.28 | -2.42 | 0.69 | 12.46 | 56.27 | 1.07 | 1.07 | 0.10 | 0.33 | 0.03 |
| Specific growth rate (%g/day) | Species | *Ompok bimaculatus* | 4 | -1.32 | 0.73 | 0.07 | -2.76 | 0.12 | 4.80 | 67.66 | 1.44 | 2.99 | 0.03 | 0.08 | <0.001 |
| Specific growth rate (%g/day) | Species | *Labeo rohita* | 128 | -1.97 | 0.24 | <0.001 | -2.44 | -1.50 | -1.46 | 81.33 | 4.97 | 6.75 | <0.001 | <0.001 | <0.001 |
| Specific growth rate (%g/day) | Species | *Cyprinus carpio* | 9 | 0.07 | 0.37 | 0.84 | -0.66 | 0.81 | 6333.19 | 73.28 | 0.61 | 1.16 | <0.001 | 0.02 | <0.001 |
| Specific growth rate (%g/day) | Species | *Oreochromis niloticus* | 33 | -0.60 | 0.27 | 0.03 | -1.14 | -0.06 | 318.30 | 68.18 | 1.64 | 2.26 | <0.001 | 0.04 | 0.66 |
| Specific growth rate (%g/day) | Species | *Lateolabrax maculatus* | 12 | -1.10 | 0.68 | 0.10 | -2.43 | 0.23 | 26.06 | 84.11 | 4.46 | 5.04 | <0.001 | 0.04 | 0.03 |
| Specific growth rate (%g/day) | Species | *Oncorhynchus mykiss* | 6 | -191.87 | 77.99 | 0.01 | -344.73 | -39.00 | -6.00 | 91.13 | 29660.14 | 30686.95 | <0.001 | 0.02 | 0.03 |
| Specific growth rate (%g/day) | Species | *Oreochromis* sp. | 8 | -6.14 | 1.61 | <0.001 | -9.29 | -2.99 | -7.19 | 84.63 | 12.59 | 18.52 | <0.001 | <0.001 | <0.001 |
| Specific growth rate (%g/day) | Species | *Cirrhinus mrigala* | 8 | -5.37 | 3.16 | 0.09 | -11.56 | 0.81 | -6.93 | 91.41 | 58.17 | 61.75 | <0.001 | 0.18 | 0.15 |
| Specific growth rate (%g/day) | Species | *Tilapia* sp*.* | 5 | -2.94 | 2.81 | 0.30 | -8.45 | 2.57 | -2.78 | 92.65 | 34.98 | 34.98 | <0.001 | 0.23 | 0.37 |
| Specific growth rate (%g/day) | Species | *Oreochromis mossambicus* | 12 | -2.90 | 0.78 | <0.001 | -4.42 | -1.38 | -6.51 | 78.30 | 4.84 | 6.92 | <0.001 | <0.001 | <0.001 |
| Specific growth rate (%g/day) | Species | *Clarias gariepinus* | 9 | -0.42 | 0.51 | 0.41 | -1.43 | 0.59 | 184.05 | 63.11 | 1.47 | 1.47 | 0.01 | 0.18 | 0.36 |
| Specific growth rate (%g/day) | Species | *Micropterus salmoides* | 8 | 0.10 | 0.60 | 0.86 | -1.08 | 1.28 | 2827.56 | 67.02 | 1.78 | 3.16 | <0.001 | 0.72 | <0.001 |
| Specific growth rate (%g/day) | Species | *Ctenopharyngodon idella* | 9 | -2.23 | 0.83 | 0.01 | -3.86 | -0.61 | -2.06 | 78.48 | 4.00 | 6.12 | <0.001 | <0.001 | <0.001 |
| Specific growth rate (%g/day) | Species | *Megaleporinus obtusidens* | 6 | -0.97 | 0.54 | 0.07 | -2.04 | 0.10 | 18.49 | 63.53 | 1.11 | 1.11 | 0.02 | 0.02 | <0.001 |
| Specific growth rate (%g/day) | Species | *Tilapia rendalli* | 8 | -1.98 | 0.52 | <0.001 | -3.00 | -0.96 | -0.15 | 47.94 | 1.00 | 2.24 | 0.06 | <0.001 | <0.001 |
| Specific growth rate (%g/day) | Species | *Dicentrarchus labrax* | 3 | -2.27 | 1.08 | 0.04 | -4.40 | -0.15 | -0.77 | 63.88 | 2.21 | 4.98 | 0.06 | 0.33 | 0.02 |
| Specific growth rate (%g/day) | Species | *Heterobranchus longifilis* | 4 | -5.62 | 2.10 | 0.01 | -9.74 | -1.49 | -3.51 | 82.00 | 11.53 | 15.40 | <0.001 | 0.08 | <0.001 |
| Specific growth rate (%g/day) | Species | *Lateolabrax japonicus* | 12 | 0.22 | 0.21 | 0.31 | -0.20 | 0.63 | 970.46 | <0.001 | <0.001 | <0.001 | 0.64 | 0.09 | 0.96 |
| Specific growth rate (%g/day) | Species | *Betta splendens* | 3 | 3.14 | 1.03 | <0.001 | 1.13 | 5.15 | -1.83 | 47.49 | 1.51 | 1.51 | 0.15 | 0.33 | 0.05 |
| Specific growth rate (%g/day) | Species | *Lates calcarifer* | 3 | -0.14 | 0.47 | 0.76 | -1.07 | 0.78 | 565.32 | <0.001 | <0.001 | <0.001 | 1.00 | 0.33 | 0.94 |
| Specific growth rate (%g/day) | Species | *Mastacembelus armatus* | 3 | 14.60 | 8.50 | 0.09 | -2.06 | 31.26 | -2.95 | 91.48 | 174.91 | 174.91 | <0.001 | 0.33 | <0.001 |
| Feed Conversion Ratio | Species | *Ompok bimaculatus* | 4 | 0.94 | 0.82 | 0.25 | -0.66 | 2.54 | 13.46 | 74.68 | 1.97 | 1.97 | 0.01 | 0.33 | 0.05 |
| Feed Conversion Ratio | Species | *Labeo rohita* | 128 | 1.86 | 0.24 | <0.001 | 1.38 | 2.33 | 14.74 | 81.94 | 5.19 | 7.11 | <0.001 | <0.001 | <0.001 |
| Feed Conversion Ratio | Species | *Oreochromis niloticus* | 24 | 0.55 | 0.25 | 0.03 | 0.06 | 1.05 | 275.60 | 43.06 | 0.62 | 1.33 | 0.01 | <0.001 | <0.001 |
| Feed Conversion Ratio | Species | *Lateolabrax maculatus* | 14 | 0.54 | 0.46 | 0.25 | -0.37 | 1.45 | 172.54 | 76.02 | 2.15 | 2.15 | <0.001 | 0.05 | 0.05 |
| Feed Conversion Ratio | Species | *Oreochromis* sp. | 8 | 2.27 | 0.73 | <0.001 | 0.84 | 3.70 | -2.03 | 69.54 | 2.70 | 3.51 | <0.001 | <0.001 | <0.001 |
| Feed Conversion Ratio | Species | *Cyprinus carpio* | 7 | -0.15 | 0.46 | 0.74 | -1.05 | 0.75 | 1148.86 | 77.16 | 0.67 | 1.40 | <0.001 | 0.07 | <0.001 |
| Feed Conversion Ratio | Species | *Cirrhinus mrigala* | 8 | 5.94 | 2.35 | 0.01 | 1.33 | 10.54 | -7.13 | 89.02 | 32.55 | 38.78 | <0.001 | 0.01 | <0.001 |
| Feed Conversion Ratio | Species | *Tilapia* sp. | 5 | 2.48 | 1.80 | 0.17 | -1.06 | 6.01 | -1.87 | 90.81 | 14.39 | 14.39 | <0.001 | 0.08 | <0.001 |
| Feed Conversion Ratio | Species | *Oreochromis mossambicus* | 12 | 2.28 | 0.66 | <0.001 | 0.99 | 3.57 | -3.11 | 74.10 | 3.36 | 3.87 | <0.001 | <0.001 | <0.001 |
| Feed Conversion Ratio | Species | *Micropterus salmoides* | 13 | -0.11 | 0.34 | 0.76 | -0.78 | 0.57 | 4474.52 | 52.98 | 0.81 | 0.81 | 0.01 | 0.77 | 0.92 |
| Feed Conversion Ratio | Species | *Megaleporinus obtusidens* | 6 | 0.96 | 0.53 | 0.07 | -0.08 | 2.00 | 18.88 | 62.06 | 1.03 | 1.03 | 0.02 | 0.02 | <0.001 |
| Feed Conversion Ratio | Species | *Tilapia rendalli* | 8 | 4.60 | 1.07 | <0.001 | 2.50 | 6.71 | -6.55 | 72.13 | 5.88 | 8.77 | <0.001 | <0.001 | <0.001 |
| Feed Conversion Ratio | Species | *Clarias gariepinus* | 5 | -0.79 | 0.86 | 0.35 | -2.47 | 0.89 | 25.46 | 73.21 | 2.66 | 2.66 | <0.001 | 0.48 | 0.78 |
| Feed Conversion Ratio | Species | *Heterobranchus longifilis* | 4 | 2.45 | 1.03 | 0.02 | 0.44 | 4.47 | -1.45 | 67.38 | 2.66 | 2.66 | 0.03 | 0.08 | <0.001 |
| Feed Conversion Ratio | Species | *Lateolabrax japonicus* | 8 | -0.48 | 0.44 | 0.28 | -1.35 | 0.39 | 123.67 | 62.03 | 0.97 | 1.15 | 0.01 | 0.21 | <0.001 |
| Feed Conversion Ratio | Species | *Betta splendens* | 3 | -0.66 | 0.49 | 0.17 | -1.61 | 0.29 | 23.47 | <0.001 | <0.001 | <0.001 | 0.84 | 0.16 | 0.56 |
| Feed Conversion Ratio | Species | *Leporinus macrocephalus* | 6 | 0.40 | 0.35 | 0.24 | -0.27 | 1.08 | 136.93 | <0.001 | <0.001 | <0.001 | 0.63 | 0.14 | 0.09 |
| Feed Conversion Ratio | Species | *Rhamdia quelen* | 5 | 0.71 | 0.33 | 0.03 | 0.07 | 1.36 | 32.71 | <0.001 | <0.001 | <0.001 | 0.92 | 0.02 | 0.35 |
| Feed Conversion Ratio | Species | *Lates calcarifer* | 3 | 1.15 | 0.76 | 0.13 | -0.35 | 2.65 | 5.70 | 49.33 | 0.86 | 0.86 | 0.14 | 0.33 | 0.05 |
| Feed Conversion Ratio | Species | *Mastacembelus armatus* | 3 | -9.00 | 4.39 | 0.04 | -17.60 | -0.41 | -2.86 | 85.64 | 47.12 | 57.61 | <0.001 | 0.33 | <0.001 |
| Feed Conversion Ratio | Species | *Ctenopharyngodon idella* | 7 | 1.07 | 0.29 | <0.001 | 0.51 | 1.64 | 16.40 | <0.001 | <0.001 | <0.001 | 0.90 | 0.01 | 0.14 |
| Protein efficiency ratio | Species | *Sparus aurata* | 3 | -1.24 | 0.95 | 0.19 | -3.09 | 0.61 | 4.48 | 65.31 | 1.75 | 1.75 | 0.06 | 0.33 | 0.02 |
| Protein efficiency ratio | Species | *Ompok bimaculatus* | 4 | -0.85 | 0.80 | 0.28 | -2.42 | 0.71 | 17.05 | 74.02 | 1.87 | 2.96 | 0.01 | 0.33 | 0.07 |
| Protein efficiency ratio | Species | *Labeo rohita* | 124 | -1.37 | 0.21 | <0.001 | -1.77 | -0.97 | 129.48 | 78.00 | 3.62 | 4.60 | <0.001 | <0.001 | <0.001 |
| Protein efficiency ratio | Species | *Oreochromis niloticus* | 25 | -0.73 | 0.25 | <0.001 | -1.22 | -0.24 | 155.72 | 46.98 | 0.70 | 1.21 | 0.01 | 0.15 | <0.001 |
| Protein efficiency ratio | Species | *Oreochromis* sp. | 8 | -2.52 | 0.96 | 0.01 | -4.40 | -0.64 | -3.16 | 80.19 | 5.28 | 7.14 | <0.001 | 0.01 | <0.001 |
| Protein efficiency ratio | Species | *Cirrhinus mrigala* | 8 | 2.58 | 1.75 | 0.14 | -0.86 | 6.02 | -3.37 | 88.75 | 17.87 | 19.94 | <0.001 | 0.40 | <0.001 |
| Protein efficiency ratio | Species | *Oreochromis mossambicus* | 8 | -2.37 | 0.48 | <0.001 | -3.31 | -1.42 | -2.51 | 32.59 | 0.59 | 1.57 | 0.17 | <0.001 | <0.001 |
| Protein efficiency ratio | Species | *Cyprinus carpio* | 4 | -10.61 | 5.34 | 0.05 | -21.07 | -0.14 | -3.86 | 85.74 | 92.31 | 71.56 | <0.001 | 0.08 | <0.001 |
| Protein efficiency ratio | Species | *Clarias gariepinus* | 9 | -0.20 | 0.45 | 0.66 | -1.09 | 0.69 | 880.24 | 55.78 | 1.02 | 1.02 | 0.02 | 0.61 | 0.84 |
| Protein efficiency ratio | Species | *Ctenopharyngodon idella* | 5 | -6.52 | 1.68 | <0.001 | -9.81 | -3.24 | -4.55 | 69.07 | 8.59 | 13.80 | 0.01 | 0.02 | <0.001 |
| Protein efficiency ratio | Species | *Tilapia rendalli* | 8 | -0.80 | 0.30 | 0.01 | -1.39 | -0.21 | 40.03 | <0.001 | <0.001 | <0.001 | 0.98 | <0.001 | 0.23 |
| Protein efficiency ratio | Species | *Dicentrarchus labrax* | 3 | -1.61 | 0.69 | 0.02 | -2.96 | -0.25 | 1.46 | 33.06 | 0.48 | 2.19 | 0.22 | 0.33 | 0.09 |
| Protein efficiency ratio | Species | *Lateolabrax japonicus* | 8 | -0.10 | 0.25 | 0.71 | -0.59 | 0.40 | 3350.88 | <0.001 | <0.001 | <0.001 | 0.72 | 0.40 | 0.16 |
| Protein efficiency ratio | Species | *Leporinus macrocephalus* | 6 | -0.45 | 0.35 | 0.19 | -1.12 | 0.23 | 108.95 | <0.001 | <0.001 | <0.001 | 0.66 | 0.06 | 0.09 |
| Protein efficiency ratio | Species | *Rhamdia quelen* | 5 | -0.62 | 0.33 | 0.06 | -1.26 | 0.01 | 44.29 | <0.001 | <0.001 | <0.001 | 0.91 | 0.02 | 0.32 |
| Atrial natriuretic peptide | Species | *Labeo rohita* | 98 | -2.74 | 0.57 | <0.001 | -3.85 | -1.63 | -47.79 | 89.66 | 22.44 | 25.30 | <0.001 | <0.001 | <0.001 |
| Atrial natriuretic peptide | Species | *Cyprinus carpio* | 4 | -13.68 | 5.98 | 0.02 | -25.40 | -1.95 | -3.92 | 82.84 | 98.84 | 110.63 | <0.001 | 0.08 | <0.001 |
| Weight gain (%) | Species | *Sparus aurata* | 3 | -0.60 | 0.52 | 0.26 | -1.62 | 0.43 | 29.49 | 10.03 | 0.08 | 0.08 | 0.33 | 0.33 | 0.14 |
| Weight gain (%) | Species | *Ompok bimaculatus* | 4 | -0.24 | 0.36 | 0.50 | -0.94 | 0.46 | 261.85 | <0.001 | <0.001 | <0.001 | 0.94 | 0.08 | 0.54 |
| Weight gain (%) | Species | *Labeo rohita* | 129 | -1.66 | 0.35 | <0.001 | -2.34 | -0.97 | 51.64 | 86.88 | 10.35 | 14.71 | <0.001 | <0.001 | <0.001 |
| Weight gain (%) | Species | *Cyprinus carpio* | 6 | -2.22 | 1.18 | 0.06 | -4.53 | 0.10 | -1.31 | 76.64 | 4.27 | 7.27 | <0.001 | <0.001 | <0.001 |
| Weight gain (%) | Species | *Oreochromis niloticus* | 33 | -1.20 | 0.44 | 0.01 | -2.07 | -0.32 | 55.64 | 81.07 | 4.30 | 6.06 | <0.001 | <0.001 | <0.001 |
| Weight gain (%) | Species | *Lateolabrax maculatus* | 14 | -0.80 | 0.63 | 0.20 | -2.03 | 0.43 | 70.80 | 84.13 | 4.43 | 4.43 | <0.001 | 0.10 | 0.90 |
| Weight gain (%) | Species | *Oreochromis* sp. | 8 | -6.10 | 1.65 | <0.001 | -9.34 | -2.86 | -7.17 | 85.00 | 13.29 | 19.50 | <0.001 | <0.001 | <0.001 |
| Weight gain (%) | Species | *Cirrhinus mrigala* | 8 | -5.54 | 3.21 | 0.08 | -11.84 | 0.75 | -7.00 | 91.44 | 60.09 | 63.51 | <0.001 | 0.18 | 0.19 |
| Weight gain (%) | Species | *Tilapia* sp. | 5 | -1.91 | 2.63 | 0.47 | -7.07 | 3.26 | 0.28 | 92.41 | 30.97 | 30.97 | <0.001 | 0.82 | 0.65 |
| Weight gain (%) | Species | *Clarias gariepinus* | 9 | -0.42 | 0.59 | 0.48 | -1.57 | 0.74 | 188.51 | 70.52 | 2.17 | 2.72 | <0.001 | 0.26 | 0.09 |
| Weight gain (%) | Species | *Micropterus salmoides* | 13 | 0.37 | 0.50 | 0.46 | -0.61 | 1.34 | 358.37 | 72.36 | 2.17 | 2.96 | <0.001 | 0.86 | 0.01 |
| Weight gain (%) | Species | *Ctenopharyngodon idella* | 13 | -1.60 | 0.53 | <0.001 | -2.63 | -0.56 | 6.59 | 73.36 | 2.28 | 3.79 | <0.001 | <0.001 | <0.001 |
| Weight gain (%) | Species | *Megaleporinus obtusidens* | 6 | -0.91 | 0.56 | 0.11 | -2.01 | 0.20 | 22.13 | 66.00 | 1.23 | 1.23 | 0.01 | 0.02 | <0.001 |
| Weight gain (%) | Species | *Oreochromis mossambicus* | 4 | -0.17 | 0.41 | 0.67 | -0.98 | 0.63 | 497.82 | <0.001 | <0.001 | <0.001 | 0.97 | 0.08 | 0.66 |
| Weight gain (%) | Species | *Dicentrarchus labrax* | 3 | -2.66 | 0.95 | <0.001 | -4.51 | -0.80 | -1.37 | 47.75 | 1.29 | 3.69 | 0.15 | 0.33 | 0.05 |
| Weight gain (%) | Species | *Heterobranchus longifilis* | 4 | -5.15 | 1.95 | 0.01 | -8.97 | -1.34 | -3.42 | 81.26 | 10.08 | 13.05 | <0.001 | 0.08 | <0.001 |
| Weight gain (%) | Species | *Lateolabrax japonicus* | 12 | 0.27 | 0.21 | 0.21 | -0.15 | 0.68 | 631.75 | <0.001 | <0.001 | <0.001 | 0.66 | 0.09 | 0.08 |
| Weight gain (%) | Species | *Betta splendens* | 3 | 2.60 | 0.65 | <0.001 | 1.32 | 3.87 | -1.29 | <0.001 | <0.001 | 0.44 | 0.55 | 0.33 | 0.27 |
| Weight gain (%) | Species | *Leporinus macrocephalus* | 6 | -1.37 | 0.53 | 0.01 | -2.41 | -0.33 | 6.30 | 44.84 | 0.75 | 1.09 | 0.11 | <0.001 | 0.01 |
| Weight gain (%) | Species | *Rhamdia quelen* | 5 | 0.52 | 0.34 | 0.13 | -0.15 | 1.19 | 66.66 | 6.21 | 0.04 | 0.04 | 0.37 | 0.08 | 0.05 |
| Weight gain (%) | Species | *Lates calcarifer* | 3 | -0.80 | 0.49 | 0.10 | -1.76 | 0.16 | 15.02 | <0.001 | <0.001 | <0.001 | 0.93 | 0.33 | 0.69 |
| Weight gain (%) | Species | *Mastacembelus armatus* | 3 | 13.12 | 7.72 | 0.09 | -2.00 | 28.25 | -2.93 | 91.41 | 134.15 | 134.15 | <0.001 | 0.33 | <0.001 |
| Whole body crude protein content (%) | Species | *Ompok bimaculatus* | 4 | -5.72 | 1.40 | <0.001 | -8.47 | -2.97 | -3.53 | 65.47 | 4.84 | 9.27 | 0.03 | 0.08 | <0.001 |
| Whole body crude protein content (%) | Species | *Labeo rohita* | 124 | -0.03 | 0.19 | 0.88 | -0.41 | 0.35 | 549775.80 | 78.02 | 3.23 | 3.31 | <0.001 | 0.23 | <0.001 |
| Whole body crude protein content (%) | Species | *Oreochromis niloticus* | 21 | -0.13 | 0.22 | 0.57 | -0.56 | 0.31 | 4975.64 | 30.39 | 0.31 | 0.53 | 0.09 | <0.001 | <0.001 |
| Whole body crude protein content (%) | Species | *Oreochromis* sp. | 5 | -0.96 | 0.40 | 0.02 | -1.74 | -0.19 | 15.81 | <0.001 | <0.001 | <0.001 | 0.58 | 0.02 | 0.10 |
| Whole body crude protein content (%) | Species | *Cirrhinus mrigala* | 8 | -8.29 | 2.82 | <0.001 | -13.82 | -2.77 | -7.55 | 90.45 | 39.49 | 52.63 | <0.001 | 0.90 | <0.001 |
| Whole body crude protein content (%) | Species | *Cyprinus carpio* | 4 | -2.10 | 1.03 | 0.04 | -4.11 | -0.09 | -0.51 | 54.99 | 2.25 | 3.97 | 0.08 | 0.08 | 0.01 |
| Whole body crude protein content (%) | Species | *Micropterus salmoides* | 13 | -0.12 | 0.44 | 0.78 | -0.98 | 0.74 | 3351.33 | 68.06 | 1.66 | 1.66 | <0.001 | 0.44 | 0.49 |
| Whole body crude protein content (%) | Species | *Ctenopharyngodon idella* | 5 | -2.26 | 2.08 | 0.28 | -6.34 | 1.82 | -1.24 | 89.35 | 16.18 | 16.18 | <0.001 | 0.79 | 0.43 |
| Whole body crude protein content (%) | Species | *Clarias gariepinus* | 5 | 7.06 | 5.43 | 0.19 | -3.58 | 17.69 | -4.61 | 88.39 | 109.55 | 109.55 | <0.001 | 0.48 | 0.01 |
| Whole body crude protein content (%) | Species | *Dicentrarchus labrax* | 3 | -0.60 | 0.48 | 0.21 | -1.55 | 0.35 | 28.84 | <0.001 | <0.001 | <0.001 | 0.83 | 0.33 | 0.54 |
| Whole body crude protein content (%) | Species | *Lateolabrax maculatus* | 8 | 0.08 | 0.23 | 0.71 | -0.36 | 0.53 | 4322.11 | <0.001 | <0.001 | <0.001 | 0.67 | 0.06 | 0.28 |
| Whole body crude protein content (%) | Species | *Lateolabrax japonicus* | 9 | 0.52 | 0.25 | 0.03 | 0.04 | 1.01 | 116.90 | <0.001 | <0.001 | <0.001 | 0.72 | 0.02 | 0.50 |
| Whole body crude protein content (%) | Species | *Leporinus macrocephalus* | 6 | 2.60 | 0.62 | <0.001 | 1.38 | 3.83 | -2.60 | 40.28 | 0.91 | 0.91 | 0.14 | <0.001 | <0.001 |
| Whole body crude protein content (%) | Species | *Rhamdia quelen* | 5 | -0.90 | 0.34 | 0.01 | -1.58 | -0.23 | 18.47 | 1.89 | 0.01 | 0.07 | 0.40 | 0.02 | 0.05 |
| Whole body crude lipid content (%) | Species | *Ompok bimaculatus* | 4 | -0.08 | 0.36 | 0.83 | -0.78 | 0.63 | 2633.25 | <0.001 | <0.001 | <0.001 | 0.62 | 1.00 | 0.29 |
| Whole body crude lipid content (%) | Species | *Labeo rohita* | 111 | -0.82 | 0.25 | <0.001 | -1.31 | -0.32 | 530.71 | 83.08 | 5.41 | 7.00 | <0.001 | <0.001 | 0.64 |
| Whole body crude lipid content (%) | Species | *Oreochromis niloticus* | 18 | -1.19 | 0.36 | <0.001 | -1.89 | -0.49 | 30.77 | 59.95 | 1.28 | 1.79 | <0.001 | 0.01 | <0.001 |
| Whole body crude lipid content (%) | Species | *Oreochromis* sp. | 5 | -0.45 | 0.37 | 0.23 | -1.18 | 0.28 | 90.67 | <0.001 | <0.001 | <0.001 | 0.87 | 0.02 | 0.27 |
| Whole body crude lipid content (%) | Species | *Cirrhinus mrigala* | 8 | -6.11 | 2.31 | 0.01 | -10.65 | -1.58 | -7.18 | 89.28 | 35.64 | 34.41 | <0.001 | <0.001 | 0.01 |
| Whole body crude lipid content (%) | Species | *Cyprinus carpio* | 4 | -3.40 | 2.07 | 0.10 | -7.46 | 0.65 | -2.67 | 81.17 | 13.25 | 13.25 | <0.001 | 0.08 | <0.001 |
| Whole body crude lipid content (%) | Species | *Micropterus salmoides* | 13 | -0.13 | 0.86 | 0.88 | -1.82 | 1.56 | 2966.41 | 85.93 | 7.85 | 7.85 | <0.001 | 0.44 | 0.94 |
| Whole body crude lipid content (%) | Species | *Ctenopharyngodon idella* | 5 | -3.47 | 0.52 | <0.001 | -4.49 | -2.45 | -3.40 | <0.001 | <0.001 | <0.001 | 0.72 | 0.19 | 0.18 |
| Whole body crude lipid content (%) | Species | *Clarias gariepinus* | 5 | -0.72 | 0.41 | 0.08 | -1.51 | 0.08 | 32.50 | 7.89 | 0.07 | 0.59 | 0.36 | 0.08 | 0.06 |
| Whole body crude lipid content (%) | Species | *Dicentrarchus labrax* | 3 | -1.62 | 0.76 | 0.03 | -3.12 | -0.12 | 1.38 | 44.14 | 0.78 | 0.78 | 0.17 | 0.33 | 0.06 |
| Whole body crude lipid content (%) | Species | *Lateolabrax maculatus* | 8 | -0.17 | 0.22 | 0.45 | -0.61 | 0.27 | 1067.94 | <0.001 | <0.001 | <0.001 | 1.00 | <0.001 | 0.72 |
| Whole body crude lipid content (%) | Species | *Lateolabrax japonicus* | 9 | -0.43 | 0.24 | 0.08 | -0.91 | 0.05 | 176.69 | <0.001 | <0.001 | <0.001 | 0.98 | 0.04 | 0.69 |
| Whole body crude lipid content (%) | Species | *Leporinus macrocephalus* | 6 | -3.92 | 0.83 | <0.001 | -5.55 | -2.28 | -4.50 | 47.59 | 1.87 | 4.11 | 0.09 | <0.001 | <0.001 |
| Whole body crude lipid content (%) | Species | *Rhamdia quelen* | 5 | -0.94 | 0.40 | 0.02 | -1.72 | -0.16 | 16.83 | 24.97 | 0.20 | 0.20 | 0.25 | 0.02 | 0.02 |
| Whole body ash content (%) | Species | *Ompok bimaculatus* | 4 | -0.09 | 0.36 | 0.80 | -0.79 | 0.61 | 1829.54 | <0.001 | <0.001 | <0.001 | 0.65 | 1.00 | 0.80 |
| Whole body ash content (%) | Species | *Labeo rohita* | 119 | -0.20 | 0.16 | 0.23 | -0.52 | 0.12 | 11571.03 | 71.68 | 2.05 | 2.93 | <0.001 | <0.001 | <0.001 |
| Whole body ash content (%) | Species | *Oreochromis niloticus* | 21 | 0.56 | 0.18 | <0.001 | 0.21 | 0.92 | 235.05 | <0.001 | <0.001 | <0.001 | 0.82 | 0.05 | 0.18 |
| Whole body ash content (%) | Species | *Oreochromis* sp. | 5 | -0.08 | 0.37 | 0.83 | -0.80 | 0.64 | 3020.77 | <0.001 | <0.001 | <0.001 | 1.00 | 0.02 | 0.86 |
| Whole body ash content (%) | Species | *Cirrhinus mrigala* | 8 | -2.92 | 0.70 | <0.001 | -4.30 | -1.54 | -4.40 | 61.31 | 2.25 | 3.79 | 0.01 | <0.001 | <0.001 |
| Whole body ash content (%) | Species | *Cyprinus carpio* | 4 | 0.92 | 0.54 | 0.09 | -0.15 | 1.98 | 14.35 | <0.001 | <0.001 | 0.30 | 0.56 | 0.08 | 0.17 |
| Whole body ash content (%) | Species | *Micropterus salmoides* | 13 | -0.09 | 0.27 | 0.73 | -0.61 | 0.43 | 6091.70 | 25.97 | 0.24 | 0.24 | 0.18 | 0.08 | 0.18 |
| Whole body ash content (%) | Species | *Ctenopharyngodon idella* | 5 | -0.62 | 0.53 | 0.24 | -1.67 | 0.42 | 44.65 | 53.77 | 0.74 | 0.74 | 0.07 | 0.05 | <0.001 |
| Whole body ash content (%) | Species | *Clarias gariepinus* | 5 | 0.77 | 0.38 | 0.04 | 0.02 | 1.51 | 27.77 | <0.001 | <0.001 | <0.001 | 0.91 | 0.02 | 0.33 |
| Whole body ash content (%) | Species | *Dicentrarchus labrax* | 3 | 0.42 | 0.58 | 0.47 | -0.72 | 1.56 | 62.42 | 27.26 | 0.28 | 0.28 | 0.25 | 0.16 | 0.10 |
| Whole body ash content (%) | Species | *Lateolabrax japonicus* | 9 | 0.13 | 0.24 | 0.58 | -0.34 | 0.61 | 1948.46 | <0.001 | <0.001 | <0.001 | 0.93 | 0.49 | 0.51 |
| Whole body ash content (%) | Species | *Lateolabrax maculatus* | 7 | 0.18 | 0.27 | 0.51 | -0.35 | 0.70 | 834.17 | <0.001 | <0.001 | <0.001 | 1.00 | <0.001 | 0.51 |
| Whole body ash content (%) | Species | *Rhamdia quelen* | 5 | 0.01 | 0.32 | 0.97 | -0.62 | 0.64 | 110738.47 | <0.001 | <0.001 | <0.001 | 0.70 | 0.82 | 0.24 |
| Tetrachlorobiphenyl | Species | *Labeo rohita* | 5 | -1.78 | 1.11 | 0.11 | -3.96 | 0.40 | 1.07 | 79.17 | 4.78 | 6.53 | <0.001 | 0.23 | 0.12 |
| Apparent protein digestibility (%) | Species | *Labeo rohita* | 95 | -1.56 | 0.22 | <0.001 | -2.00 | -1.12 | 54.93 | 75.16 | 3.12 | 4.77 | <0.001 | <0.001 | <0.001 |
| Apparent protein digestibility (%) | Species | *Tilapia* sp. | 4 | -7.34 | 3.50 | 0.04 | -14.20 | -0.48 | -3.71 | 93.36 | 41.29 | 51.99 | <0.001 | 0.08 | <0.001 |
| Apparent protein digestibility (%) | Species | *Oreochromis mossambicus* | 8 | -2.78 | 0.54 | <0.001 | -3.84 | -1.71 | -4.02 | 39.05 | 0.90 | 1.67 | 0.12 | <0.001 | <0.001 |
| Apparent protein digestibility (%) | Species | *Oreochromis niloticus* | 8 | -0.58 | 0.95 | 0.55 | -2.45 | 1.30 | 84.64 | 77.90 | 5.05 | 6.20 | <0.001 | 0.11 | <0.001 |
| Apparent protein digestibility (%) | Species | *Dicentrarchus labrax* | 3 | -8.76 | 3.57 | 0.01 | -15.76 | -1.76 | -2.85 | 79.43 | 28.89 | 39.78 | 0.01 | 0.33 | <0.001 |
| Apparent protein digestibility (%) | Species | *Micropterus salmoides* | 4 | 0.85 | 1.04 | 0.41 | -1.19 | 2.89 | 17.08 | 82.92 | 3.57 | 3.57 | <0.001 | 0.33 | 0.13 |
| Apparent lipid digestibility (%) | Species | *Labeo rohita* | 31 | -0.76 | 0.22 | <0.001 | -1.20 | -0.32 | 174.42 | 4<0.001 | 0.59 | 1.33 | 0.01 | <0.001 | <0.001 |
| Apparent lipid digestibility (%) | Species | *Oreochromis niloticus* | 8 | -2.20 | 0.61 | <0.001 | -3.40 | -1.00 | -1.66 | 49.50 | 1.41 | 2.61 | 0.05 | 0.06 | <0.001 |
| Apparent lipid digestibility (%) | Species | *Micropterus salmoides* | 4 | 1.56 | 0.59 | 0.01 | 0.40 | 2.71 | 2.35 | 47.85 | 0.65 | 0.65 | 0.12 | 0.08 | 0.02 |
| Apparent ash digestibility (%) | Species | *Labeo rohita* | 19 | -1.63 | 0.65 | 0.01 | -2.92 | -0.35 | 8.40 | 81.67 | 5.43 | 7.24 | <0.001 | <0.001 | <0.001 |
| Apparent phosphorous digestibility (%) | Species | *Labeo rohita* | 11 | -1.81 | 0.73 | 0.01 | -3.25 | -0.38 | 1.84 | 77.56 | 4.05 | 4.05 | <0.001 | <0.001 | <0.001 |
| Apparent calcium digestibility (%) | Species | *Labeo rohita* | 7 | -0.34 | 0.63 | 0.59 | -1.57 | 0.89 | 227.45 | 67.83 | 1.87 | 1.87 | <0.001 | 1.00 | <0.001 |
| Apparent manganese digestibility (%) | Species | *Labeo rohita* | 7 | -1.14 | 0.71 | 0.11 | -2.53 | 0.26 | 13.79 | 71.93 | 2.52 | 2.52 | <0.001 | 0.03 | <0.001 |
| Apparent copper digestibility (%) | Species | *Labeo rohita* | 7 | -0.62 | 0.72 | 0.39 | -2.03 | 0.79 | 63.68 | 73.42 | 2.64 | 2.64 | <0.001 | 0.38 | 0.55 |
| Apparent iron digestibility (%) | Species | *Labeo rohita* | 7 | -1.46 | 0.84 | 0.08 | -3.10 | 0.17 | 5.55 | 76.83 | 3.68 | 3.68 | <0.001 | 0.01 | <0.001 |
| Whole body phosphorus content (mg/g) | Species | *Labeo rohita* | 7 | -1.40 | 1.15 | 0.22 | -3.65 | 0.85 | 6.70 | 84.28 | 7.66 | 8.53 | <0.001 | 0.14 | 0.17 |
| Whole body calcium content (mg/g) | Species | *Labeo rohita* | 7 | -0.68 | 1.37 | 0.62 | -3.36 | 2.00 | 51.07 | 84.41 | 10.62 | 10.62 | <0.001 | 1.00 | <0.001 |
| Whole body manganese content (µg/g) | Species | *Labeo rohita* | 7 | -3.77 | 2.11 | 0.07 | -7.90 | 0.37 | -5.11 | 89.97 | 27.13 | 27.13 | <0.001 | <0.001 | <0.001 |
| Whole body copper content (µg/g) | Species | *Labeo rohita* | 7 | -5.87 | 1.20 | <0.001 | -8.21 | -3.52 | -6.22 | 64.14 | 5.79 | 8.60 | 0.01 | <0.001 | <0.001 |
| Whole body iron content (µg/g) | Species | *Labeo rohita* | 7 | -2.58 | 0.88 | <0.001 | -4.30 | -0.86 | -2.96 | 74.08 | 3.80 | 3.80 | <0.001 | <0.001 | <0.001 |
| Faecal phosphorus concentration (mg/g) | Species | *Labeo rohita* | 7 | 4.75 | 1.23 | <0.001 | 2.34 | 7.17 | -5.81 | 76.39 | 7.05 | 8.59 | <0.001 | <0.001 | <0.001 |
| Survival Rate (%) | Species | *Sparus aurata* | 3 | -2.05 | 1.67 | 0.22 | -5.32 | 1.23 | -0.24 | 81.78 | 5.76 | 5.76 | <0.001 | 0.33 | <0.001 |
| Survival Rate (%) | Species | *Ompok bimaculatus* | 4 | -0.36 | 0.36 | 0.32 | -1.06 | 0.35 | 117.56 | <0.001 | <0.001 | <0.001 | 0.70 | 0.33 | 0.31 |
| Survival Rate (%) | Species | *Oreochromis niloticus* | 11 | 0.21 | 0.23 | 0.36 | -0.24 | 0.65 | 988.36 | <0.001 | <0.001 | <0.001 | 0.67 | 1.00 | 0.49 |
| Survival Rate (%) | Species | *Lateolabrax maculatus* | 14 | 0.13 | 0.19 | 0.48 | -0.24 | 0.51 | 2992.20 | <0.001 | <0.001 | <0.001 | 0.98 | <0.001 | 0.30 |
| Survival Rate (%) | Species | *Labeo rohita* | 8 | -0.06 | 0.29 | 0.85 | -0.62 | 0.51 | 9646.22 | <0.001 | <0.001 | <0.001 | 1.00 | 0.01 | 0.60 |
| Survival Rate (%) | Species | *Oreochromis* sp. | 8 | 0.02 | 0.29 | 0.94 | -0.55 | 0.59 | 69184.40 | <0.001 | <0.001 | <0.001 | 0.99 | 1.00 | 0.50 |
| Survival Rate (%) | Species | *Tilapia* sp. | 5 | -1.58 | 1.20 | 0.19 | -3.93 | 0.76 | 2.68 | 84.57 | 5.12 | 5.12 | <0.001 | 0.23 | <0.001 |
| Survival Rate (%) | Species | *Clarias gariepinus* | 9 | 0.10 | 0.39 | 0.79 | -0.66 | 0.87 | 3200.09 | 40.98 | 0.54 | 1.14 | 0.09 | 1.00 | <0.001 |
| Survival Rate (%) | Species | *Micropterus salmoides* | 8 | -0.18 | 0.43 | 0.68 | -1.01 | 0.66 | 991.92 | 45.61 | 0.65 | 0.65 | 0.08 | 0.80 | 0.01 |
| Survival Rate (%) | Species | *Ctenopharyngodon idella* | 13 | -0.23 | 0.22 | 0.29 | -0.67 | 0.20 | 904.11 | 3.74 | 0.02 | 0.25 | 0.41 | 0.01 | <0.001 |
| Survival Rate (%) | Species | *Heterobranchus longifilis* | 4 | -0.54 | 1.03 | 0.60 | -2.55 | 1.47 | 49.04 | 83.10 | 3.49 | 3.49 | <0.001 | 0.33 | 0.03 |
| Survival Rate (%) | Species | *Lateolabrax japonicus* | 12 | 0.50 | 0.21 | 0.02 | 0.08 | 0.92 | 173.37 | <0.001 | <0.001 | <0.001 | 0.90 | <0.001 | 0.41 |
| Survival Rate (%) | Species | *Rhamdia quelen* | 5 | -0.86 | 0.35 | 0.02 | -1.56 | -0.17 | 20.87 | 8.16 | 0.05 | 0.18 | 0.36 | 0.02 | 0.05 |
| Survival Rate (%) | Species | *Mastacembelus armatus* | 3 | 2.88 | 0.69 | <0.001 | 1.52 | 4.23 | -1.61 | <0.001 | <0.001 | <0.001 | 0.38 | 0.33 | 0.17 |
| Average daily feed intake (%/g/day) | Species | *Oncorhynchus mykiss* | 6 | -4.53 | 3.82 | 0.24 | -12.02 | 2.95 | -4.88 | 91.62 | 69.49 | 78.73 | <0.001 | 0.14 | 0.59 |
| Feed intake (g/ﬁsh/day) | Species | *Sparus aurata* | 3 | -0.16 | 0.64 | 0.80 | -1.43 | 1.10 | 420.74 | 40.51 | 0.50 | 0.50 | 0.19 | 1.00 | 0.94 |
| Feed intake (g/ﬁsh/day) | Species | *Oreochromis niloticus* | 9 | -1.50 | 0.70 | 0.03 | -2.87 | -0.12 | 6.47 | 74.37 | 3.17 | 4.35 | <0.001 | 0.01 | <0.001 |
| Feed intake (g/ﬁsh/day) | Species | *Lateolabrax maculatus* | 14 | -0.76 | 0.49 | 0.12 | -1.71 | 0.19 | 78.69 | 77.91 | 2.50 | 2.50 | <0.001 | 0.04 | 0.05 |
| Feed intake (g/ﬁsh/day) | Species | *Labeo rohita* | 14 | 0.47 | 0.42 | 0.27 | -0.36 | 1.30 | 230.98 | 63.10 | 1.48 | 1.48 | <0.001 | 0.05 | 0.11 |
| Feed intake (g/ﬁsh/day) | Species | *Oreochromis* sp. | 5 | -3.71 | 1.70 | 0.03 | -7.05 | -0.37 | -3.60 | 86.05 | 11.33 | 15.03 | <0.001 | 0.08 | <0.001 |
| Feed intake (g/ﬁsh/day) | Species | *Oreochromis mossambicus* | 12 | -1.89 | 0.54 | <0.001 | -2.95 | -0.84 | 0.87 | 67.05 | 2.21 | 3.25 | <0.001 | <0.001 | <0.001 |
| Feed intake (g/ﬁsh/day) | Species | *Ctenopharyngodon idella* | 6 | -3.28 | 0.70 | <0.001 | -4.65 | -1.90 | -3.85 | 40.40 | 1.14 | 2.28 | 0.14 | <0.001 | <0.001 |
| Feed intake (g/ﬁsh/day) | Species | *Megaleporinus obtusidens* | 6 | -0.56 | 0.79 | 0.48 | -2.11 | 1.00 | 68.20 | 80.16 | 2.84 | 2.84 | <0.001 | 0.72 | <0.001 |
| Feed intake (g/ﬁsh/day) | Species | *Tilapia rendalli* | 8 | -0.57 | 0.30 | 0.06 | -1.15 | 0.02 | 87.30 | <0.001 | <0.001 | <0.001 | 0.92 | <0.001 | 0.12 |
| Feed intake (g/ﬁsh/day) | Species | *Dicentrarchus labrax* | 3 | -1.04 | 0.50 | 0.04 | -2.03 | -0.05 | 7.69 | <0.001 | <0.001 | <0.001 | 0.86 | 0.33 | 0.58 |
| Feed intake (g/ﬁsh/day) | Species | *Lateolabrax japonicus* | 9 | 0.03 | 0.24 | 0.89 | -0.44 | 0.50 | 33602.33 | <0.001 | <0.001 | <0.001 | 1.00 | 0.01 | 0.97 |
| Condition factor (g/cm3) | Species | *Sparus aurata* | 3 | 1.74 | 1.42 | 0.22 | -1.06 | 4.53 | 0.83 | 79.94 | 4.61 | 4.61 | 0.01 | 0.33 | <0.001 |
| Condition factor (g/cm3) | Species | *Ompok bimaculatus* | 4 | -0.15 | 0.35 | 0.68 | -0.84 | 0.55 | 703.48 | <0.001 | <0.001 | <0.001 | 0.99 | 0.08 | 0.75 |
| Condition factor (g/cm3) | Species | *Cyprinus carpio* | 3 | 2.24 | 0.29 | <0.001 | 1.67 | 2.82 | -0.71 | 57.74 | 0.15 | 0.15 | 0.09 | 0.33 | 0.03 |
| Condition factor (g/cm3) | Species | *Micropterus salmoides* | 3 | -15.89 | 11.37 | 0.16 | -38.18 | 6.39 | -2.95 | 92.31 | 351.18 | 351.18 | <0.001 | 1.00 | <0.001 |
| Condition factor (g/cm3) | Species | *Dicentrarchus labrax* | 3 | -0.23 | 0.47 | 0.63 | -1.16 | 0.70 | 215.48 | <0.001 | <0.001 | <0.001 | 0.88 | 0.33 | 0.64 |
| Condition factor (g/cm3) | Species | *Lateolabrax maculatus* | 4 | -0.92 | 0.37 | 0.01 | -1.65 | -0.19 | 14.08 | <0.001 | <0.001 | <0.001 | 1.00 | 0.08 | 0.80 |
| Condition factor (g/cm3) | Species | *Lateolabrax japonicus* | 9 | 0.42 | 0.25 | 0.09 | -0.06 | 0.91 | 183.51 | <0.001 | <0.001 | <0.001 | 0.53 | 0.02 | 0.05 |
| Condition factor (g/cm3) | Species | *Betta splendens* | 3 | -1.00 | 0.51 | 0.05 | -2.00 | <0.001 | 8.53 | <0.001 | <0.001 | 0.60 | 0.48 | 0.33 | 0.23 |
| Condition factor (g/cm3) | Species | *Mastacembelus armatus* | 3 | 0.16 | 0.48 | 0.75 | -0.79 | 1.10 | 466.89 | <0.001 | <0.001 | <0.001 | 0.49 | 1.00 | 0.68 |
| Hepatosomatic Index (%) | Species | *Sparus aurata* | 3 | -0.40 | 0.58 | 0.49 | -1.55 | 0.74 | 67.72 | 27.97 | 0.29 | 0.29 | 0.25 | 0.33 | 0.29 |
| Hepatosomatic Index (%) | Species | *Oreochromis niloticus* | 16 | -0.62 | 0.23 | 0.01 | -1.07 | -0.18 | 142.28 | <0.001 | <0.001 | 0.19 | 0.48 | <0.001 | 0.05 |
| Hepatosomatic Index (%) | Species | *Oreochromis* sp*.* | 5 | -0.51 | 0.37 | 0.17 | -1.24 | 0.22 | 67.53 | <0.001 | <0.001 | <0.001 | 0.97 | 0.02 | 0.49 |
| Hepatosomatic Index (%) | Species | *Micropterus salmoides* | 13 | -0.55 | 0.23 | 0.02 | -0.99 | -0.10 | 153.16 | <0.001 | <0.001 | <0.001 | 0.55 | <0.001 | 0.01 |
| Hepatosomatic Index (%) | Species | *Labeo rohita* | 15 | 0.50 | 0.34 | 0.14 | -0.16 | 1.16 | 212.57 | 58.03 | 0.89 | 1.89 | <0.001 | 0.01 | <0.001 |
| Hepatosomatic Index (%) | Species | *Ctenopharyngodon idella* | 6 | -1.99 | 0.73 | 0.01 | -3.41 | -0.57 | -0.17 | 63.40 | 1.96 | 1.92 | 0.02 | <0.001 | <0.001 |
| Hepatosomatic Index (%) | Species | *Dicentrarchus labrax* | 3 | -0.87 | 0.59 | 0.14 | -2.03 | 0.29 | 12.23 | 24.13 | 0.25 | 0.25 | 0.27 | 0.33 | 0.11 |
| Hepatosomatic Index (%) | Species | *Lateolabrax maculatus* | 4 | 0.27 | 0.36 | 0.45 | -0.43 | 0.97 | 211.84 | <0.001 | <0.001 | <0.001 | 0.90 | 0.08 | 0.45 |
| Hepatosomatic Index (%) | Species | *Lateolabrax japonicus* | 9 | <0.001 | 0.24 | 1.00 | -0.48 | 0.48 | 18018441.21 | <0.001 | <0.001 | <0.001 | 0.79 | 0.91 | 0.34 |
| Hepatosomatic Index (%) | Species | *Leporinus macrocephalus* | 6 | -1.57 | 0.44 | <0.001 | -2.44 | -0.71 | 3.33 | 18.20 | 0.21 | 1.20 | 0.30 | <0.001 | 0.02 |
| Viscerosomatic index (%) | Species | *Oreochromis* sp. | 5 | -0.01 | 0.37 | 0.98 | -0.73 | 0.71 | 195960.15 | <0.001 | <0.001 | <0.001 | 1.00 | 1.00 | 0.98 |
| Viscerosomatic index (%) | Species | *Micropterus salmoides* | 13 | -0.06 | 0.22 | 0.80 | -0.49 | 0.38 | 15162.19 | <0.001 | <0.001 | <0.001 | 0.63 | 0.06 | 0.40 |
| Viscerosomatic index (%) | Species | *Ctenopharyngodon idella* | 9 | -3.88 | 1.02 | <0.001 | -5.87 | -1.88 | -6.70 | 80.51 | 6.97 | 7.04 | <0.001 | <0.001 | <0.001 |
| Viscerosomatic index (%) | Species | *Dicentrarchus labrax* | 3 | -0.07 | 0.47 | 0.88 | -0.99 | 0.85 | 2386.36 | <0.001 | <0.001 | <0.001 | 0.99 | 0.33 | 0.91 |
| Viscerosomatic index (%) | Species | *Lateolabrax maculatus* | 4 | -0.24 | 0.36 | 0.50 | -0.94 | 0.46 | 263.16 | <0.001 | <0.001 | <0.001 | 0.91 | 0.08 | 0.47 |
| Viscerosomatic index (%) | Species | *Lateolabrax japonicus* | 9 | 0.30 | 0.24 | 0.22 | -0.18 | 0.77 | 377.23 | <0.001 | <0.001 | <0.001 | 0.95 | 0.05 | 0.93 |
| Viscerosomatic index (%) | Species | *Mastacembelus armatus* | 3 | -0.92 | 0.50 | 0.07 | -1.89 | 0.06 | 10.72 | <0.001 | <0.001 | <0.001 | 0.78 | 0.33 | 0.48 |
| Intestinosomatic index (%) | Species | *Oreochromis niloticus* | 12 | -0.12 | 0.27 | 0.66 | -0.66 | 0.42 | 3154.33 | 18.32 | 0.17 | 0.33 | 0.26 | 0.32 | 0.01 |
| Intestinosomatic index (%) | Species | *Lateolabrax maculatus* | 5 | -0.45 | 0.32 | 0.17 | -1.09 | 0.19 | 90.11 | <0.001 | <0.001 | <0.001 | 0.68 | 0.03 | 0.13 |
| Intestinosomatic index (%) | Species | *Labeo rohita* | 4 | -0.30 | 0.41 | 0.46 | -1.11 | 0.50 | 165.44 | <0.001 | <0.001 | <0.001 | 1.00 | 0.08 | 0.80 |
| Intestinosomatic index (%) | Species | *Dicentrarchus labrax* | 3 | 0.13 | 0.47 | 0.78 | -0.79 | 1.06 | 637.91 | <0.001 | <0.001 | <0.001 | 1.00 | 0.33 | 0.95 |
| Intestinosomatic index (%) | Species | *Ctenopharyngodon idella* | 3 | -0.14 | 0.41 | 0.74 | -0.94 | 0.67 | 620.97 | <0.001 | <0.001 | <0.001 | 0.95 | 0.33 | 0.76 |
| Intestinosomatic index (%) | Species | *Micropterus salmoides* | 8 | 0.21 | 0.43 | 0.62 | -0.63 | 1.05 | 685.22 | 46.42 | 0.67 | 0.67 | 0.07 | 0.72 | 0.08 |
| Intraperitoneal fat (%) | Species | *Ctenopharyngodon idella* | 5 | -2.00 | 0.82 | 0.01 | -3.60 | -0.40 | -0.20 | 64.49 | 2.03 | 2.03 | 0.02 | 0.02 | <0.001 |
| Intraperitoneal fat (%) | Species | *Lateolabrax japonicus* | 8 | -1.64 | 0.54 | <0.001 | -2.71 | -0.58 | 3.38 | 67.48 | 1.54 | 1.54 | <0.001 | <0.001 | <0.001 |
| Total antioxidant capacity (U/mL) | Species | *Lateolabrax maculatus* | 10 | 1.50 | 0.48 | <0.001 | 0.56 | 2.44 | 7.10 | 67.72 | 1.50 | 2.28 | <0.001 | <0.001 | <0.001 |
| Total antioxidant capacity (U/mL) | Species | *Micropterus salmoides* | 13 | 1.18 | 0.60 | 0.05 | <0.001 | 2.36 | 22.83 | 77.43 | 3.51 | 3.51 | <0.001 | 0.06 | 0.65 |
| Total antioxidant capacity (U/mL) | Species | *Tilapia rendalli* | 8 | -0.57 | 0.30 | 0.06 | -1.15 | 0.02 | 87.30 | <0.001 | <0.001 | <0.001 | 0.92 | <0.001 | 0.12 |
| Total antioxidant capacity (U/mL) | Species | *Lateolabrax japonicus* | 9 | 1.00 | 0.30 | <0.001 | 0.42 | 1.58 | 25.50 | 21.33 | 0.17 | 0.17 | 0.25 | 0.01 | 0.02 |
| Total antioxidant capacity (U/mL) | Species | *Betta splendens* | 3 | 4.28 | 1.46 | <0.001 | 1.41 | 7.15 | -2.37 | 71.73 | 4.55 | 4.55 | 0.03 | 0.33 | 0.01 |
| Total antioxidant capacity (U/mL) | Species | *Mastacembelus armatus* | 3 | 4.88 | 2.56 | 0.06 | -0.15 | 9.91 | -2.52 | 83.95 | 16.25 | 16.25 | <0.001 | 0.33 | <0.001 |
| Total antioxidant capacity (U/mL) | Species | *Ctenopharyngodon idella* | 3 | -2.93 | 1.15 | 0.01 | -5.18 | -0.68 | -1.66 | 59.99 | 2.33 | 4.73 | 0.08 | 0.33 | 0.03 |
| Total cholesterol (mmol/L) | Species | *Oreochromis niloticus* | 15 | -1.61 | 0.29 | <0.001 | -2.18 | -1.04 | 7.27 | 14.38 | 0.18 | 0.71 | 0.29 | <0.001 | <0.001 |
| Total cholesterol (mmol/L) | Species | *Oreochromis* sp. | 5 | -0.82 | 0.38 | 0.03 | -1.57 | -0.08 | 23.43 | <0.001 | <0.001 | <0.001 | 1.00 | 0.02 | 0.68 |
| Total cholesterol (mmol/L) | Species | *Ctenopharyngodon idella* | 8 | 0.05 | 1.12 | 0.97 | -2.16 | 2.25 | 15111.76 | 85.23 | 7.35 | 7.35 | <0.001 | 0.90 | <0.001 |
| Total cholesterol (mmol/L) | Species | *Lateolabrax maculatus* | 10 | -1.39 | 0.57 | 0.01 | -2.50 | -0.29 | 9.78 | 76.19 | 2.34 | 2.34 | <0.001 | <0.001 | <0.001 |
| Total cholesterol (mmol/L) | Species | *Lateolabrax japonicus* | 11 | -0.27 | 0.22 | 0.22 | -0.69 | 0.16 | 582.09 | <0.001 | <0.001 | <0.001 | 0.87 | 0.03 | 0.04 |
| Triacylglycerol (mmol/L) | Species | *Lateolabrax maculatus* | 10 | -1.48 | 0.42 | <0.001 | -2.30 | -0.66 | 7.58 | 59.23 | 1.02 | 1.50 | 0.01 | <0.001 | <0.001 |
| Triacylglycerol (mmol/L) | Species | *Lateolabrax japonicus* | 11 | -0.51 | 0.22 | 0.02 | -0.94 | -0.08 | 150.80 | <0.001 | <0.001 | <0.001 | 0.92 | <0.001 | 0.05 |
| Glucose (mmol/L) | Species | *Ompok bimaculatus* | 4 | 3.29 | 1.01 | <0.001 | 1.30 | 5.27 | -2.58 | 68.56 | 2.66 | 2.66 | 0.02 | 0.08 | <0.001 |
| Glucose (mmol/L) | Species | *Oreochromis niloticus* | 6 | -1.99 | 1.07 | 0.06 | -4.10 | 0.11 | -0.21 | 82.89 | 4.91 | 7.45 | <0.001 | 0.06 | <0.001 |
| Glucose (mmol/L) | Species | *Megaleporinus obtusidens* | 6 | 0.05 | 0.56 | 0.93 | -1.04 | 1.14 | 8507.98 | 67.28 | 1.23 | 1.23 | 0.01 | 1.00 | 0.01 |
| Glucose (mmol/L) | Species | *Tilapia rendalli* | 6 | -0.17 | 0.48 | 0.72 | -1.11 | 0.77 | 792.74 | 88.82 | 1.22 | 1.22 | <0.001 | 1.00 | 0.58 |
| Glucose (mmol/L) | Species | *Lateolabrax maculatus* | 10 | -0.49 | 0.29 | 0.09 | -1.05 | 0.07 | 151.45 | 30.53 | 0.25 | 0.25 | 0.16 | <0.001 | <0.001 |
| Glucose (mmol/L) | Species | *Lateolabrax japonicus* | 12 | -0.31 | 0.21 | 0.15 | -0.72 | 0.11 | 481.98 | <0.001 | <0.001 | <0.001 | 0.96 | 0.01 | 0.18 |
| Blood urea nitrogen (mmol/L) | Species | *Tilapia rendalli* | 6 | 1.64 | 0.49 | <0.001 | 0.68 | 2.61 | 2.55 | 87.09 | 1.26 | 1.26 | <0.001 | <0.001 | <0.001 |
| Blood urea nitrogen (mmol/L) | Species | *Lateolabrax japonicus* | 6 | 0.41 | 0.29 | 0.15 | -0.16 | 0.99 | 127.84 | <0.001 | <0.001 | <0.001 | 1.00 | 0.03 | 0.95 |
| Blood urea nitrogen (mmol/L) | Species | *Lateolabrax maculatus* | 7 | -0.12 | 0.27 | 0.66 | -0.65 | 0.41 | 1948.66 | <0.001 | <0.001 | <0.001 | 0.87 | 0.22 | 0.46 |
| Blood urea nitrogen (mmol/L) | Species | *Ctenopharyngodon idella* | 4 | -21.66 | 2.78 | <0.001 | -27.10 | -16.22 | -3.97 | <0.001 | <0.001 | <0.001 | 0.56 | 0.06 | 0.15 |
| Diamine oxidase (U/L) | Species | *Lateolabrax maculatus* | 5 | -0.70 | 0.81 | 0.39 | -2.29 | 0.89 | 34.17 | 78.56 | 2.56 | 2.56 | <0.001 | 0.48 | 0.38 |
| Diamine oxidase (U/L) | Species | *Micropterus salmoides* | 7 | 0.23 | 0.91 | 0.80 | -1.55 | 2.02 | 486.16 | 83.31 | 4.69 | 4.69 | <0.001 | 1.00 | 0.31 |
| Diamine oxidase (U/L) | Species | *Lateolabrax japonicus* | 3 | 0.20 | 0.41 | 0.63 | -0.61 | 1.00 | 288.28 | <0.001 | <0.001 | <0.001 | 0.82 | 0.33 | 0.57 |
| Lactate (µmol/L) | Species | *Lateolabrax maculatus* | 5 | -0.43 | 0.44 | 0.32 | -1.29 | 0.42 | 97.07 | 41.26 | 0.39 | 0.75 | 0.15 | 0.23 | 0.07 |
| Lactate (µmol/L) | Species | *Lateolabrax japonicus* | 3 | -0.22 | 0.41 | 0.60 | -1.02 | 0.59 | 244.69 | <0.001 | <0.001 | <0.001 | 0.91 | 0.33 | 0.68 |
| Lipopolysaccharide (pg/mL) | Species | *Lateolabrax japonicus* | 4 | -0.67 | 0.45 | 0.13 | -1.55 | 0.21 | 30.32 | 24.43 | 0.20 | 0.20 | 0.26 | 1.00 | 0.87 |
| Lipopolysaccharide (pg/mL) | Species | *Micropterus salmoides* | 5 | -1.45 | 1.02 | 0.16 | -3.45 | 0.55 | 4.15 | 82.16 | 3.96 | 3.96 | <0.001 | 0.23 | 0.01 |
| Superoxide dismutase (U/mL) | Species | *Sparus aurata* | 3 | -1.71 | 0.56 | <0.001 | -2.80 | -0.62 | 0.93 | <0.001 | <0.001 | <0.001 | 0.76 | 0.33 | 0.46 |
| Superoxide dismutase (U/mL) | Species | *Oreochromis niloticus* | 9 | 0.17 | 0.20 | 0.40 | -0.22 | 0.55 | 1258.20 | <0.001 | <0.001 | <0.001 | 1.00 | 0.36 | 0.65 |
| Superoxide dismutase (U/mL) | Species | *Lateolabrax maculatus* | 11 | 2.73 | 0.73 | <0.001 | 1.30 | 4.17 | -5.35 | 81.81 | 4.36 | 6.17 | <0.001 | <0.001 | <0.001 |
| Superoxide dismutase (U/mL) | Species | *Cyprinus carpio* | 6 | 0.67 | 0.20 | <0.001 | 0.28 | 1.06 | 45.37 | <0.001 | <0.001 | <0.001 | 0.83 | 1.00 | 0.67 |
| Superoxide dismutase (U/mL) | Species | *Micropterus salmoides* | 13 | -1.53 | 0.97 | 0.12 | -3.43 | 0.37 | 8.43 | 87.21 | 9.55 | 11.09 | <0.001 | 0.10 | <0.001 |
| Superoxide dismutase (U/mL) | Species | *Ctenopharyngodon idella* | 8 | -0.37 | 0.95 | 0.70 | -2.22 | 1.49 | 221.77 | 80.60 | 5.38 | 5.38 | <0.001 | 0.72 | <0.001 |
| Superoxide dismutase (U/mL) | Species | *Labeo rohita* | 11 | 0.41 | 0.68 | 0.54 | -0.92 | 1.73 | 241.26 | 78.50 | 3.85 | 3.85 | <0.001 | 0.88 | 0.17 |
| Superoxide dismutase (U/mL) | Species | *Lateolabrax japonicus* | 9 | 3.41 | 1.00 | <0.001 | 1.45 | 5.36 | -6.02 | 85.48 | 6.24 | 8.36 | <0.001 | 0.01 | <0.001 |
| Superoxide dismutase (U/mL) | Species | *Betta splendens* | 3 | 11.89 | 2.07 | <0.001 | 7.82 | 15.96 | -2.92 | <0.001 | <0.001 | <0.001 | 0.52 | 0.33 | 0.26 |
| Superoxide dismutase (U/mL) | Species | *Mastacembelus armatus* | 3 | 4.30 | 2.47 | 0.08 | -0.53 | 9.14 | -2.38 | 84.48 | 12.42 | 12.42 | <0.001 | 0.33 | <0.001 |
| Catalase activity (kU/L) | Species | *Sparus aurata* | 3 | 4.28 | 1.44 | <0.001 | 1.46 | 7.10 | -2.37 | 60.15 | 3.74 | 3.74 | 0.08 | 0.33 | 0.03 |
| Catalase activity (kU/L) | Species | *Oreochromis niloticus* | 9 | -0.11 | 0.20 | 0.58 | -0.50 | 0.28 | 2921.35 | <0.001 | <0.001 | <0.001 | 0.95 | 0.92 | 0.37 |
| Catalase activity (kU/L) | Species | *Lateolabrax maculatus* | 11 | 1.03 | 0.45 | 0.02 | 0.15 | 1.90 | 29.05 | 67.57 | 1.38 | 1.38 | <0.001 | <0.001 | <0.001 |
| Catalase activity (kU/L) | Species | *Cyprinus carpio* | 6 | 0.42 | 0.20 | 0.03 | 0.03 | 0.80 | 125.33 | <0.001 | <0.001 | 0.09 | 0.47 | 0.72 | 0.17 |
| Catalase activity (kU/L) | Species | *Ctenopharyngodon idella* | 5 | -1.99 | 0.46 | <0.001 | -2.88 | -1.09 | -0.14 | <0.001 | <0.001 | <0.001 | 0.67 | 0.02 | 0.12 |
| Catalase activity (kU/L) | Species | *Labeo rohita* | 11 | 0.88 | 0.38 | 0.02 | 0.14 | 1.61 | 43.88 | 44.22 | 0.66 | 0.66 | 0.06 | <0.001 | <0.001 |
| Catalase activity (kU/L) | Species | *Lateolabrax japonicus* | 9 | 1.73 | 0.50 | <0.001 | 0.75 | 2.71 | 2.54 | 63.74 | 1.39 | 1.83 | <0.001 | <0.001 | <0.001 |
| Catalase activity (kU/L) | Species | *Betta splendens* | 3 | 6.05 | 1.38 | <0.001 | 3.34 | 8.75 | -2.68 | 27.93 | 1.65 | 1.65 | 0.25 | 0.33 | 0.10 |
| Catalase activity (kU/L) | Species | *Micropterus salmoides* | 11 | 1.98 | 0.72 | 0.01 | 0.57 | 3.40 | -0.27 | 77.53 | 4.07 | 4.93 | <0.001 | 0.02 | 0.80 |
| Catalase activity (kU/L) | Species | *Mastacembelus armatus* | 3 | 3.55 | 0.85 | <0.001 | 1.89 | 5.22 | -2.09 | 14.41 | 0.33 | 2.42 | 0.31 | 0.33 | 0.13 |
| Glutathione S-transferase (pmol/min/mg protein) | Species | *Oreochromis niloticus* | 5 | -0.22 | 0.22 | 0.32 | -0.66 | 0.22 | 376.55 | <0.001 | <0.001 | <0.001 | 0.99 | 0.02 | 0.65 |
| Glutathione S-transferase (pmol/min/mg protein) | Species | *Lateolabrax japonicus* | 3 | 0.88 | 0.43 | 0.04 | 0.03 | 1.72 | 12.00 | <0.001 | <0.001 | <0.001 | 0.80 | 0.33 | 0.50 |
| Glutathione S-transferase (pmol/min/mg protein) | Species | *Micropterus salmoides* | 6 | 1.60 | 0.98 | 0.10 | -0.33 | 3.52 | 3.04 | 79.18 | 4.25 | 5.44 | <0.001 | 0.06 | <0.001 |
| Glutathione peroxidase (U/mL) | Species | *Cyprinus carpio* | 3 | 0.12 | 0.47 | 0.80 | -0.81 | 1.04 | 824.69 | <0.001 | <0.001 | <0.001 | 0.94 | 0.16 | 0.72 |
| Glutathione peroxidase (U/mL) | Species | *Oreochromis niloticus* | 4 | -0.04 | 0.45 | 0.93 | -0.93 | 0.85 | 8473.88 | 12.98 | 0.11 | 0.11 | 0.33 | 1.00 | 1.00 |
| Glutathione peroxidase (U/mL) | Species | *Lateolabrax maculatus* | 8 | 0.42 | 0.26 | 0.10 | -0.08 | 0.92 | 166.29 | <0.001 | <0.001 | <0.001 | 0.93 | <0.001 | 0.13 |
| Glutathione peroxidase (U/mL) | Species | *Lateolabrax japonicus* | 8 | 0.16 | 0.25 | 0.54 | -0.34 | 0.65 | 1252.86 | <0.001 | <0.001 | <0.001 | 0.97 | 0.18 | 0.24 |
| Malondialdehyde (U/mL) | Species | *Lateolabrax maculatus* | 11 | -0.71 | 0.38 | 0.06 | -1.46 | 0.03 | 72.59 | 60.30 | 0.92 | 0.92 | <0.001 | 0.01 | <0.001 |
| Malondialdehyde (U/mL) | Species | *Cyprinus carpio* | 6 | -0.23 | 0.19 | 0.23 | -0.61 | 0.15 | 413.78 | <0.001 | <0.001 | <0.001 | 0.82 | 0.14 | 0.16 |
| Malondialdehyde (U/mL) | Species | *Micropterus salmoides* | 13 | 4.52 | 1.12 | <0.001 | 2.33 | 6.71 | -10.55 | 86.78 | 10.36 | 13.44 | <0.001 | <0.001 | <0.001 |
| Malondialdehyde (U/mL) | Species | *Oreochromis niloticus* | 4 | -0.14 | 0.41 | 0.74 | -0.95 | 0.67 | 817.71 | <0.001 | <0.001 | <0.001 | 0.77 | 0.75 | 0.59 |
| Malondialdehyde (U/mL) | Species | *Lateolabrax japonicus* | 9 | 0.01 | 0.24 | 0.95 | -0.46 | 0.49 | 165373.15 | <0.001 | <0.001 | <0.001 | 0.80 | 0.92 | 0.62 |
| Malondialdehyde (U/mL) | Species | *Labeo rohita* | 8 | 0.22 | 0.61 | 0.72 | -0.97 | 1.40 | 649.90 | 69.15 | 1.98 | 1.98 | <0.001 | 0.05 | <0.001 |
| Malondialdehyde (U/mL) | Species | *Mastacembelus armatus* | 3 | -3.90 | 1.48 | 0.01 | -6.81 | -1.00 | -2.24 | 66.54 | 4.35 | 4.35 | 0.05 | 0.33 | 0.01 |
| TBARS (nmol/g) | Species | *Sparus aurata* | 3 | 2.00 | 0.80 | 0.01 | 0.42 | 3.57 | -0.11 | 42.70 | 0.83 | 2.74 | 0.17 | 0.33 | 0.06 |
| TBARS (nmol/g) | Species | *Oreochromis niloticus* | 6 | -0.12 | 0.37 | 0.74 | -0.84 | 0.60 | 1565.02 | 62.43 | 0.49 | 0.49 | 0.02 | 0.47 | <0.001 |
| Lysozyme activity (U/mL) | Species | *Oreochromis niloticus* | 9 | 1.80 | 0.64 | <0.001 | 0.55 | 3.05 | 1.71 | 82.72 | 2.99 | 2.99 | <0.001 | 0.92 | 0.45 |
| Lysozyme activity (U/mL) | Species | *Labeo rohita* | 5 | 0.11 | 0.62 | 0.86 | -1.11 | 1.33 | 1641.92 | 58.21 | 1.12 | 1.12 | 0.05 | 1.00 | 0.89 |
| Lysozyme activity (U/mL) | Species | *Lateolabrax maculatus* | 9 | 3.44 | 0.63 | <0.001 | 2.21 | 4.67 | -6.08 | 62.19 | 2.04 | 2.04 | 0.01 | <0.001 | <0.001 |
| Lysozyme activity (U/mL) | Species | *Lateolabrax japonicus* | 4 | -0.76 | 0.38 | 0.05 | -1.51 | -0.01 | 22.68 | <0.001 | <0.001 | <0.001 | 0.82 | 0.75 | 0.69 |
| Lysozyme activity (U/mL) | Species | *Betta splendens* | 3 | 3.01 | 1.10 | 0.01 | 0.86 | 5.17 | -1.73 | 54.38 | 1.91 | 1.91 | 0.11 | 0.33 | 0.04 |
| Lysozyme activity (U/mL) | Species | *Micropterus salmoides* | 4 | 1.71 | 1.66 | 0.30 | -1.54 | 4.95 | 1.28 | 89.40 | 9.55 | 12.80 | <0.001 | 0.75 | 0.24 |
| Peroxidase activity (OD 450 nm) | Species | *Oreochromis niloticus* | 5 | -1.31 | 0.34 | <0.001 | -1.98 | -0.65 | 6.14 | 44.44 | 3.48 | 5.61 | 0.13 | 0.02 | 0.01 |
| Plasma bactericidal activity | Species | *Oreochromis niloticus* | 5 | 0.93 | 0.49 | 0.06 | -0.02 | 1.89 | 17.10 | 74.32 | 0.88 | 0.88 | <0.001 | 0.08 | 0.02 |
| Nitro blue tetrazolium (OD 540 nm) | Species | *Oreochromis niloticus* | 5 | -0.19 | 0.55 | 0.74 | -1.27 | 0.90 | 553.13 | 81.10 | 1.25 | 1.25 | <0.001 | 0.79 | 0.80 |
| Nitro blue tetrazolium (OD 540 nm) | Species | *Labeo rohita* | 5 | 0.52 | 0.96 | 0.59 | -1.36 | 2.39 | 67.09 | 74.49 | 2.89 | 5.61 | <0.001 | 0.03 | <0.001 |
| Amylase (U/mg protein) | Species | *Ompok bimaculatus* | 4 | -1.10 | 1.40 | 0.43 | -3.85 | 1.65 | 8.79 | 87.98 | 6.72 | 6.72 | <0.001 | 0.75 | 0.13 |
| Amylase (U/mg protein) | Species | *Labeo rohita* | 32 | -2.10 | 0.54 | <0.001 | -3.15 | -1.05 | -4.02 | 82.30 | 6.46 | 7.10 | <0.001 | <0.001 | 0.01 |
| Amylase (U/mg protein) | Species | *Lateolabrax maculatus* | 11 | -1.11 | 0.37 | <0.001 | -1.83 | -0.39 | 23.25 | 60.34 | 0.83 | 1.08 | <0.001 | <0.001 | <0.001 |
| Amylase (U/mg protein) | Species | *Cyprinus carpio* | 3 | 0.91 | 0.36 | 0.01 | 0.22 | 1.61 | 10.80 | <0.001 | <0.001 | 0.48 | 0.39 | 0.33 | 0.17 |
| Amylase (U/mg protein) | Species | *Micropterus salmoides* | 13 | -0.04 | 0.41 | 0.92 | -0.85 | 0.77 | 31774.68 | 64.40 | 1.37 | 1.37 | <0.001 | 0.08 | <0.001 |
| Amylase (U/mg protein) | Species | *Ctenopharyngodon idella* | 9 | 1.65 | 1.25 | 0.19 | -0.80 | 4.09 | 3.71 | 88.24 | 10.57 | 12.64 | <0.001 | 0.92 | <0.001 |
| Amylase (U/mg protein) | Species | *Megaleporinus obtusidens* | 6 | -1.98 | 0.56 | <0.001 | -3.08 | -0.87 | -0.10 | 57.08 | 1.07 | 1.12 | 0.04 | <0.001 | <0.001 |
| Amylase (U/mg protein) | Species | *Oreochromis niloticus* | 4 | -1.03 | 0.44 | 0.02 | -1.89 | -0.17 | 10.51 | <0.001 | <0.001 | <0.001 | 0.87 | 0.08 | 0.40 |
| Amylase (U/mg protein) | Species | *Lateolabrax japonicus* | 4 | 0.48 | 1.16 | 0.68 | -1.80 | 2.76 | 62.66 | 81.14 | 3.54 | 3.54 | <0.001 | 0.06 | <0.001 |
| Amylase (U/mg protein) | Species | *Betta splendens* | 3 | 2.81 | 0.68 | <0.001 | 1.49 | 4.14 | -1.54 | <0.001 | <0.001 | <0.001 | 0.55 | 0.33 | 0.28 |
| Amylase (U/mg protein) | Species | *Mastacembelus armatus* | 3 | -0.64 | 0.48 | 0.18 | -1.59 | 0.30 | 24.72 | <0.001 | <0.001 | <0.001 | 0.98 | 0.33 | 0.83 |
| Lipase (U/mg protein) | Species | *Ompok bimaculatus* | 4 | 9.79 | 3.12 | <0.001 | 3.69 | 15.90 | -3.84 | 85.13 | 29.97 | 39.85 | <0.001 | 0.08 | <0.001 |
| Lipase (U/mg protein) | Species | *Labeo rohita* | 25 | -2.45 | 0.47 | <0.001 | -3.38 | -1.52 | -8.99 | 74.40 | 3.65 | 5.15 | <0.001 | <0.001 | <0.001 |
| Lipase (U/mg protein) | Species | *Lateolabrax maculatus* | 11 | -1.12 | 0.46 | 0.01 | -2.02 | -0.22 | 22.78 | 72.84 | 1.56 | 2.09 | <0.001 | <0.001 | <0.001 |
| Lipase (U/mg protein) | Species | *Cyprinus carpio* | 3 | 1.89 | 0.59 | <0.001 | 0.73 | 3.04 | 0.24 | 51.32 | 0.54 | 0.54 | 0.13 | 0.33 | 0.04 |
| Lipase (U/mg protein) | Species | *Micropterus salmoides* | 13 | 1.11 | 0.67 | 0.10 | -0.21 | 2.43 | 27.58 | 81.06 | 4.33 | 4.33 | <0.001 | 0.10 | <0.001 |
| Lipase (U/mg protein) | Species | *Ctenopharyngodon idella* | 9 | -1.47 | 0.46 | <0.001 | -2.36 | -0.57 | 7.10 | 55.07 | 1.01 | 1.36 | 0.02 | <0.001 | <0.001 |
| Lipase (U/mg protein) | Species | *Megaleporinus obtusidens* | 6 | -1.70 | 0.62 | 0.01 | -2.92 | -0.48 | 1.97 | 66.47 | 1.52 | 1.62 | 0.01 | <0.001 | <0.001 |
| Lipase (U/mg protein) | Species | *Oreochromis niloticus* | 4 | -1.37 | 0.46 | <0.001 | -2.26 | -0.47 | 4.20 | <0.001 | <0.001 | <0.001 | 0.89 | 0.08 | 0.43 |
| Lipase (U/mg protein) | Species | *Lateolabrax japonicus* | 4 | -0.25 | 0.37 | 0.49 | -0.97 | 0.47 | 234.39 | <0.001 | <0.001 | <0.001 | 0.95 | 1.00 | 0.58 |
| Lipase (U/mg protein) | Species | *Betta splendens* | 3 | 3.50 | 0.75 | <0.001 | 2.02 | 4.98 | -2.06 | <0.001 | <0.001 | <0.001 | 0.83 | 0.33 | 0.54 |
| Lipase (U/mg protein) | Species | *Mastacembelus armatus* | 3 | 0.67 | 0.49 | 0.17 | -0.29 | 1.64 | 22.36 | <0.001 | <0.001 | <0.001 | 0.57 | 0.33 | 0.30 |
| Trypsin (U/mg protein) | Species | *Lateolabrax maculatus* | 11 | -2.61 | 0.81 | <0.001 | -4.21 | -1.01 | -4.80 | 86.57 | 5.79 | 6.44 | <0.001 | <0.001 | <0.001 |
| Trypsin (U/mg protein) | Species | *Micropterus salmoides* | 13 | -0.71 | 0.63 | 0.26 | -1.94 | 0.52 | 85.94 | 80.40 | 3.92 | 5.05 | <0.001 | 0.01 | <0.001 |
| Trypsin (U/mg protein) | Species | *Lateolabrax japonicus* | 4 | -1.03 | 0.41 | 0.01 | -1.84 | -0.23 | 10.36 | 5.93 | 0.04 | 0.04 | 0.36 | 0.75 | 0.15 |
| Trypsin (U/mg protein) | Species | *Ctenopharyngodon idella* | 4 | 5.18 | 0.76 | <0.001 | 3.70 | 6.66 | -3.43 | <0.001 | <0.001 | 1.12 | 0.40 | 0.06 | 0.08 |
| Creatine kinase (U/mg protein) | Species | *Lateolabrax japonicus* | 3 | 1.25 | 1.90 | 0.51 | -2.47 | 4.96 | 4.43 | 87.42 | 6.91 | 6.91 | <0.001 | 0.16 | <0.001 |
| Na+, K+- ATPase (U/mg protein) | Species | *Lateolabrax japonicus* | 3 | -51.94 | 26.83 | 0.05 | -104.53 | 0.65 | -3.00 | 93.15 | 1584.76 | 1584.76 | <0.001 | 0.33 | <0.001 |
| Protease (U/mg protein) | Species | *Ompok bimaculatus* | 4 | -2.54 | 1.06 | 0.02 | -4.62 | -0.47 | -1.63 | 77.39 | 3.40 | 3.40 | <0.001 | 0.08 | <0.001 |
| Protease (U/mg protein) | Species | *Labeo rohita* | 29 | -0.93 | 0.37 | 0.01 | -1.66 | -0.20 | 99.83 | 73.05 | 2.66 | 3.25 | <0.001 | <0.001 | <0.001 |
| Protease (U/mg protein) | Species | *Cyprinus carpio* | 3 | 1.99 | 0.41 | <0.001 | 1.18 | 2.79 | -0.08 | <0.001 | <0.001 | <0.001 | 0.65 | 0.33 | 0.36 |
| Protease (U/mg protein) | Species | *Ctenopharyngodon idella* | 5 | -1.76 | 0.44 | <0.001 | -2.62 | -0.90 | 1.21 | <0.001 | <0.001 | <0.001 | 0.62 | 0.02 | 0.11 |
| Protease (U/mg protein) | Species | *Megaleporinus obtusidens* | 3 | -3.13 | 0.61 | <0.001 | -4.33 | -1.94 | -1.83 | <0.001 | <0.001 | <0.001 | 0.86 | 0.33 | 0.58 |
| Protease (U/mg protein) | Species | *Oreochromis niloticus* | 4 | -0.98 | 0.43 | 0.02 | -1.82 | -0.13 | 12.13 | <0.001 | <0.001 | <0.001 | 0.98 | 0.08 | 0.68 |
| Protease (U/mg protein) | Species | *Betta splendens* | 3 | 14.61 | 7.23 | 0.04 | 0.45 | 28.78 | -2.95 | 86.48 | 131.50 | 131.50 | <0.001 | 0.33 | <0.001 |
| Protease (U/mg protein) | Species | *Micropterus salmoides* | 8 | -3.87 | 0.93 | <0.001 | -5.69 | -2.04 | -5.94 | 71.43 | 4.62 | 4.57 | <0.001 | <0.001 | <0.001 |
| Protease (U/mg protein) | Species | *Mastacembelus armatus* | 3 | -0.47 | 0.48 | 0.33 | -1.41 | 0.47 | 49.37 | <0.001 | <0.001 | <0.001 | 0.99 | 0.33 | 0.92 |
| Hematocrit (%) | Species | *Oreochromis niloticus* | 7 | 0.57 | 0.30 | 0.06 | -0.03 | 1.16 | 76.95 | <0.001 | <0.001 | 0.05 | 0.48 | 0.01 | 0.02 |
| Hematocrit (%) | Species | *Clarias gariepinus* | 4 | 0.30 | 0.42 | 0.47 | -0.52 | 1.13 | 164.83 | <0.001 | <0.001 | 0.02 | 0.53 | 0.33 | 0.23 |
| Hematocrit (%) | Species | *Oreochromis mossambicus* | 4 | 2.21 | 0.44 | <0.001 | 1.35 | 3.08 | -0.86 | 71.76 | 0.56 | 0.56 | 0.01 | 0.08 | <0.001 |
| Hematocrit (%) | Species | *Tilapia rendalli* | 6 | -0.34 | 0.22 | 0.12 | -0.77 | 0.09 | 192.51 | 51.30 | 0.15 | 0.15 | 0.07 | 0.06 | <0.001 |
| Creatinine (mg/dL) | Species | *Clarias gariepinus* | 3 | 10.47 | 3.35 | <0.001 | 3.91 | 17.03 | -2.89 | 46.72 | 15.76 | 28.84 | 0.15 | 0.33 | 0.05 |
| Total bilirubin (mg/dL) | Species | *Labeo rohita* | 5 | 0.43 | 0.48 | 0.37 | -0.51 | 1.37 | 97.84 | 33.30 | 0.38 | 0.38 | 0.20 | 0.23 | 0.02 |
| Total bilirubin (mg/dL) | Species | *Clarias gariepinus* | 3 | -0.96 | 1.20 | 0.43 | -3.31 | 1.40 | 9.58 | 64.16 | 2.68 | 2.68 | 0.06 | 1.00 | 0.02 |
| Total protein (g/dL) | Species | *Oreochromis niloticus* | 14 | -0.42 | 0.22 | 0.06 | -0.85 | 0.01 | 295.32 | <0.001 | <0.001 | <0.001 | 0.64 | <0.001 | 0.01 |
| Total protein (g/dL) | Species | *Oreochromis* sp. | 5 | 0.51 | 0.44 | 0.24 | -0.35 | 1.38 | 67.62 | 22.52 | 0.22 | 0.22 | 0.27 | 0.08 | 0.04 |
| Total protein (g/dL) | Species | *Labeo rohita* | 6 | -0.42 | 0.93 | 0.65 | -2.24 | 1.40 | 125.17 | 78.24 | 3.80 | 3.80 | <0.001 | 0.72 | 0.87 |
| Total protein (g/dL) | Species | *Ctenopharyngodon idella* | 9 | 0.44 | 0.82 | 0.59 | -1.17 | 2.05 | 169.58 | 83.34 | 4.86 | 4.86 | <0.001 | 0.75 | <0.001 |
| Total protein (g/dL) | Species | *Oreochromis mossambicus* | 4 | 0.80 | 0.21 | <0.001 | 0.38 | 1.22 | 20.02 | 18.97 | 0.03 | 0.03 | 0.30 | 0.08 | 0.06 |
| Total protein (g/dL) | Species | *Tilapia rendalli* | 6 | -2.37 | 0.20 | <0.001 | -2.75 | -1.99 | -1.89 | <0.001 | <0.001 | <0.001 | 0.58 | <0.001 | 0.05 |
| Total protein (g/dL) | Species | *Clarias gariepinus* | 3 | -0.86 | 0.98 | 0.38 | -2.78 | 1.07 | 12.75 | 52.94 | 1.52 | 1.52 | 0.12 | 0.33 | 0.05 |
| Total protein (g/dL) | Species | *Betta splendens* | 3 | 4.58 | 2.48 | 0.06 | -0.28 | 9.43 | -2.45 | 84.21 | 15.20 | 15.20 | <0.001 | 0.33 | <0.001 |
| Triglyceride (g/dL) | Species | *Oreochromis* sp. | 5 | -1.73 | 0.49 | <0.001 | -2.70 | -0.76 | 1.43 | 18.43 | 0.23 | 0.93 | 0.30 | 0.02 | 0.03 |
| Triglyceride (g/dL) | Species | *Ctenopharyngodon idella* | 8 | -0.09 | 0.93 | 0.92 | -1.92 | 1.74 | 3738.88 | 83.55 | 5.62 | 5.62 | <0.001 | 0.72 | <0.001 |
| Triglyceride (g/dL) | Species | *Oreochromis niloticus* | 3 | -0.77 | 0.99 | 0.44 | -2.71 | 1.18 | 16.49 | 69.65 | 2.03 | 2.03 | 0.04 | 1.00 | 0.01 |
| Triglyceride (g/dL) | Species | *Mastacembelus armatus* | 3 | 153.31 | 94.20 | 0.10 | -31.32 | 337.93 | -3.00 | 91.55 | 23230.98 | 23245.57 | <0.001 | 0.33 | <0.001 |
| Phosphate (mmol/L) | Species | *Oreochromis* sp. | 5 | -0.55 | 0.37 | 0.14 | -1.28 | 0.18 | 58.02 | <0.001 | <0.001 | <0.001 | 0.98 | 0.02 | 0.51 |
| Alkaline phosphatase (IU/L) | Species | *Labeo rohita* | 4 | -0.40 | 0.42 | 0.34 | -1.22 | 0.42 | 91.85 | <0.001 | <0.001 | <0.001 | 0.81 | 0.08 | 0.34 |
| Alkaline phosphatase (IU/L) | Species | *Oreochromis mossambicus* | 4 | 0.83 | 0.23 | <0.001 | 0.39 | 1.28 | 18.22 | 28.93 | 0.06 | 0.06 | 0.24 | 0.08 | 0.04 |
| Alkaline phosphatase (IU/L) | Species | *Oreochromis niloticus* | 7 | -0.12 | 0.31 | 0.70 | -0.72 | 0.49 | 1945.66 | <0.001 | <0.001 | <0.001 | 1.00 | 0.13 | 0.51 |
| Alkaline phosphatase (IU/L) | Species | *Clarias gariepinus* | 3 | -6.27 | 1.72 | <0.001 | -9.64 | -2.90 | -2.71 | 22.78 | 2.17 | 7.01 | 0.27 | 0.33 | 0.11 |
| Alkaline phosphatase (IU/L) | Species | *Lateolabrax maculatus* | 7 | 0.43 | 0.36 | 0.23 | -0.27 | 1.13 | 136.81 | 37.04 | 0.33 | 0.63 | 0.15 | 0.24 | 0.04 |
| Alkaline phosphatase (IU/L) | Species | *Lateolabrax japonicus* | 3 | 0.42 | 0.42 | 0.31 | -0.39 | 1.24 | 61.23 | <0.001 | <0.001 | <0.001 | 0.63 | 0.33 | 0.34 |
| Alkaline phosphatase (IU/L) | Species | *Betta splendens* | 3 | 2.48 | 0.64 | <0.001 | 1.22 | 3.75 | -1.13 | <0.001 | <0.001 | 1.30 | 0.39 | 0.33 | 0.17 |
| Alkaline phosphatase (IU/L) | Species | *Mastacembelus armatus* | 3 | 7.58 | 1.36 | <0.001 | 4.91 | 10.26 | -2.80 | <0.001 | <0.001 | <0.001 | 0.65 | 0.33 | 0.35 |
| Alanine aminotransferase (IU/L) | Species | *Ompok bimaculatus* | 4 | 0.05 | 0.80 | 0.95 | -1.52 | 1.62 | 6658.84 | 75.28 | 1.92 | 1.92 | 0.01 | 1.00 | 0.26 |
| Alanine aminotransferase (IU/L) | Species | *Oreochromis* sp. | 5 | 0.49 | 0.38 | 0.20 | -0.26 | 1.24 | 73.88 | <0.001 | <0.001 | <0.001 | 0.47 | 0.02 | 0.06 |
| Alanine aminotransferase (IU/L) | Species | *Labeo rohita* | 16 | 0.03 | 0.33 | 0.93 | -0.62 | 0.67 | 78238.79 | 52.45 | 0.88 | 1.35 | 0.01 | 0.69 | 0.01 |
| Alanine aminotransferase (IU/L) | Species | *Cyprinus carpio* | 3 | -0.41 | 0.21 | 0.05 | -0.83 | 0.01 | 65.09 | <0.001 | <0.001 | <0.001 | 0.91 | 0.33 | 0.67 |
| Alanine aminotransferase (IU/L) | Species | *Oreochromis mossambicus* | 4 | 0.56 | 0.19 | <0.001 | 0.19 | 0.93 | 44.87 | <0.001 | <0.001 | 0.06 | 0.41 | 0.08 | 0.10 |
| Alanine aminotransferase (IU/L) | Species | *Clarias gariepinus* | 3 | -4.38 | 1.10 | <0.001 | -6.53 | -2.23 | -2.40 | <0.001 | <0.001 | 0.56 | 0.51 | 0.33 | 0.25 |
| Alanine aminotransferase (IU/L) | Species | *Oreochromis niloticus* | 3 | 2.57 | 0.64 | <0.001 | 1.31 | 3.82 | -1.25 | <0.001 | <0.001 | <0.001 | 0.82 | 0.33 | 0.53 |
| Alanine aminotransferase (IU/L) | Species | *Lateolabrax maculatus* | 10 | 1.50 | 0.71 | 0.03 | 0.11 | 2.89 | 7.03 | 82.33 | 3.96 | 5.07 | <0.001 | 0.01 | <0.001 |
| Alanine aminotransferase (IU/L) | Species | *Lateolabrax japonicus* | 8 | 0.08 | 0.25 | 0.75 | -0.42 | 0.58 | 4535.75 | <0.001 | <0.001 | <0.001 | 0.77 | 0.55 | 0.11 |
| Alanine aminotransferase (IU/L) | Species | *Mastacembelus armatus* | 3 | 3.82 | 3.44 | 0.27 | -2.92 | 10.55 | -2.21 | 91.52 | 31.99 | 31.99 | <0.001 | 0.33 | <0.001 |
| Alanine aminotransferase (IU/L) | Species | *Ctenopharyngodon idella* | 3 | 3.04 | 2.06 | 0.14 | -1.01 | 7.09 | -1.75 | 85.85 | 10.40 | 16.97 | <0.001 | 0.33 | <0.001 |
| Aspartate aminotransferase (IU/L) | Species | *Ompok bimaculatus* | 4 | -1.01 | 3.90 | 0.79 | -8.65 | 6.62 | 11.00 | 94.23 | 56.22 | 56.22 | <0.001 | 0.75 | 0.61 |
| Aspartate aminotransferase (IU/L) | Species | *Labeo rohita* | 16 | 0.87 | 0.48 | 0.07 | -0.07 | 1.82 | 64.35 | 72.88 | 2.64 | 3.26 | <0.001 | 0.02 | <0.001 |
| Aspartate aminotransferase (IU/L) | Species | *Cyprinus carpio* | 3 | -0.31 | 0.21 | 0.14 | -0.73 | 0.10 | 113.35 | <0.001 | <0.001 | <0.001 | 0.98 | 0.33 | 0.83 |
| Aspartate aminotransferase (IU/L) | Species | *Oreochromis mossambicus* | 4 | 0.47 | 0.19 | 0.01 | 0.11 | 0.84 | 64.39 | <0.001 | <0.001 | <0.001 | 0.39 | 0.08 | 0.09 |
| Aspartate aminotransferase (IU/L) | Species | *Clarias gariepinus* | 3 | -2.93 | 1.05 | 0.01 | -5.00 | -0.86 | -1.66 | 28.89 | 1.01 | 1.01 | 0.25 | 0.33 | 0.09 |
| Aspartate aminotransferase (IU/L) | Species | *Oreochromis niloticus* | 3 | 10.60 | 3.26 | <0.001 | 4.20 | 16.99 | -2.90 | 64.43 | 20.42 | 36.12 | 0.06 | 0.33 | 0.02 |
| Aspartate aminotransferase (IU/L) | Species | *Lateolabrax maculatus* | 10 | 0.34 | 0.58 | 0.56 | -0.80 | 1.48 | 330.37 | 77.92 | 2.50 | 2.50 | <0.001 | 0.29 | 0.07 |
| Aspartate aminotransferase (IU/L) | Species | *Lateolabrax japonicus* | 8 | 0.23 | 0.26 | 0.38 | -0.27 | 0.73 | 590.30 | <0.001 | <0.001 | <0.001 | 0.65 | 0.11 | 0.09 |
| Aspartate aminotransferase (IU/L) | Species | *Mastacembelus armatus* | 3 | 1.55 | 0.63 | 0.01 | 0.31 | 2.79 | 1.80 | 21.13 | 0.26 | 0.26 | 0.28 | 0.33 | 0.11 |
| Aspartate aminotransferase (IU/L) | Species | *Ctenopharyngodon idella* | 3 | -1.79 | 2.42 | 0.46 | -6.53 | 2.96 | 0.60 | 89.80 | 15.48 | 20.40 | <0.001 | 1.00 | 0.23 |
| Lactate dehydrogenase | Species | *Oreochromis mossambicus* | 4 | 0.39 | 0.18 | 0.03 | 0.03 | 0.76 | 95.46 | <0.001 | <0.001 | <0.001 | 0.69 | 0.08 | 0.24 |
| Lactate dehydrogenase | Species | *Labeo rohita* | 11 | -0.37 | 0.40 | 0.36 | -1.15 | 0.42 | 301.95 | 52.99 | 0.91 | 0.91 | 0.02 | 0.16 | 0.01 |
| Malate dehydrogenase | Species | *Labeo rohita* | 3 | 4.89 | 1.92 | 0.01 | 1.13 | 8.66 | -2.52 | 72.53 | 7.89 | 13.49 | 0.03 | 0.33 | 0.01 |
| Red blood cell (x10^12^L) | Species | *Oreochromis niloticus* | 7 | 0.04 | 0.31 | 0.89 | -0.57 | 0.66 | 13754.74 | <0.001 | <0.001 | <0.001 | 0.93 | 0.24 | 0.21 |
| Red blood cell (x10^12^L) | Species | *Clarias gariepinus* | 7 | 0.45 | 0.47 | 0.34 | -0.48 | 1.37 | 127.32 | 39.03 | 0.60 | 0.60 | 0.13 | 0.14 | 0.01 |
| Red blood cell (x10^12^L) | Species | *Oreochromis mossambicus* | 4 | 1.66 | 0.56 | <0.001 | 0.56 | 2.75 | 1.59 | 84.94 | 1.05 | 1.05 | <0.001 | 0.08 | <0.001 |
| Red blood cell (x10^12^L) | Species | *Tilapia rendalli* | 6 | -0.73 | 0.21 | <0.001 | -1.14 | -0.33 | 37.15 | 42.95 | 0.11 | 0.11 | 0.12 | <0.001 | <0.001 |
| Red blood cell (x10^12^L) | Species | *Heterobranchus longifilis* | 4 | -3.60 | 2.18 | 0.10 | -7.87 | 0.67 | -2.81 | 88.11 | 14.17 | 16.77 | <0.001 | 0.33 | <0.001 |
| White blood cell (x10^12^L) | Species | *Oreochromis niloticus* | 7 | 0.61 | 0.33 | 0.06 | -0.03 | 1.25 | 64.65 | <0.001 | <0.001 | <0.001 | 0.54 | 0.03 | 0.15 |
| White blood cell (x10^12^L) | Species | *Clarias gariepinus* | 7 | 1.06 | 0.43 | 0.01 | 0.22 | 1.90 | 16.74 | 22.26 | 0.29 | 0.29 | 0.26 | 0.56 | 0.32 |
| White blood cell (x10^12^L) | Species | *Oreochromis mossambicus* | 4 | 3.30 | 1.17 | <0.001 | 1.00 | 5.59 | -2.59 | 94.64 | 5.07 | 5.39 | <0.001 | 0.08 | <0.001 |
| White blood cell (x10^12^L) | Species | *Tilapia rendalli* | 6 | 0.18 | 0.77 | 0.81 | -1.33 | 1.70 | 684.13 | 94.75 | 3.36 | 3.36 | <0.001 | 1.00 | 0.13 |
| White blood cell (x10^12^L) | Species | *Heterobranchus longifilis* | 4 | 0.01 | 0.41 | 0.98 | -0.80 | 0.81 | 251032.62 | <0.001 | <0.001 | <0.001 | 0.94 | 1.00 | 0.95 |
| Haemoglobin (g/dL) | Species | *Ompok bimaculatus* | 4 | 3.83 | 0.88 | <0.001 | 2.11 | 5.55 | -2.95 | 49.03 | 1.46 | 1.46 | 0.12 | 0.08 | 0.02 |
| Haemoglobin (g/dL) | Species | *Oreochromis niloticus* | 10 | -0.47 | 0.26 | 0.07 | -0.97 | 0.04 | 165.60 | <0.001 | <0.001 | <0.001 | 0.61 | 0.15 | 0.04 |
| Haemoglobin (g/dL) | Species | *Clarias gariepinus* | 7 | 1.14 | 0.40 | <0.001 | 0.36 | 1.93 | 13.52 | 9.23 | 0.10 | 0.59 | 0.36 | <0.001 | 0.02 |
| Haemoglobin (g/dL) | Species | *Labeo rohita* | 6 | -6.02 | 1.90 | <0.001 | -9.75 | -2.28 | -5.36 | 85.65 | 15.22 | 16.68 | <0.001 | <0.001 | <0.001 |
| Haemoglobin (g/dL) | Species | *Oreochromis mossambicus* | 4 | 1.67 | 0.50 | <0.001 | 0.69 | 2.65 | 1.48 | 81.21 | 0.81 | 0.81 | <0.001 | 0.08 | <0.001 |
| Haemoglobin (g/dL) | Species | *Tilapia rendalli* | 6 | -0.11 | 0.28 | 0.70 | -0.65 | 0.43 | 1998.35 | 69.31 | 0.32 | 0.32 | 0.01 | 0.72 | 0.04 |
| Haemoglobin (g/dL) | Species | *Heterobranchus longifilis* | 4 | -1.04 | 0.82 | 0.20 | -2.64 | 0.56 | 10.23 | 64.95 | 1.68 | 1.68 | 0.04 | 0.06 | 0.01 |
| Platelet (%) | Species | Clarias gariepinus | 4 | 0.29 | 0.41 | 0.48 | -0.52 | 1.10 | 177.01 | <0.001 | <0.001 | <0.001 | 0.90 | 0.08 | 0.50 |
| Platelet (%) | Species | *Heterobranchus longifilis* | 4 | 0.97 | 0.70 | 0.17 | -0.41 | 2.34 | 12.50 | 55.54 | 1.08 | 1.08 | 0.08 | 0.08 | 0.01 |
| Packed cell volume (%) | Species | *Ompok bimaculatus* | 4 | -4.06 | 0.62 | <0.001 | -5.28 | -2.83 | -3.07 | <0.001 | <0.001 | <0.001 | 0.72 | 0.08 | 0.25 |
| Packed cell volume (%) | Species | *Clarias gariepinus* | 3 | 3.18 | 1.57 | 0.04 | 0.09 | 6.26 | -1.86 | 59.57 | 4.06 | 4.06 | 0.08 | 0.33 | 0.03 |
| Packed cell volume (%) | Species | *Oreochromis niloticus* | 3 | -1.06 | 0.69 | 0.13 | -2.43 | 0.30 | 7.17 | 41.45 | 0.60 | 0.60 | 0.18 | 0.33 | 0.07 |
| Packed cell volume (%) | Species | *Heterobranchus longifilis* | 4 | -1.42 | 1.00 | 0.16 | -3.38 | 0.54 | 3.60 | 73.42 | 2.82 | 2.82 | 0.01 | 0.07 | <0.001 |
| Mean corpuscular volume (fL) | Species | *Oreochromis niloticus* | 10 | -0.10 | 0.25 | 0.68 | -0.59 | 0.39 | 3618.99 | <0.001 | <0.001 | <0.001 | 0.93 | 0.38 | 0.91 |
| Mean corpuscular volume (fL) | Species | *Clarias gariepinus* | 7 | -0.47 | 0.35 | 0.17 | -1.15 | 0.21 | 113.20 | <0.001 | <0.001 | <0.001 | 0.68 | 0.14 | 0.16 |
| Mean corpuscular volume (fL) | Species | *Oreochromis mossambicus* | 4 | 0.11 | 0.18 | 0.54 | -0.25 | 0.47 | 1229.96 | <0.001 | <0.001 | <0.001 | 0.41 | 0.75 | 0.22 |
| Mean corpuscular volume (fL) | Species | *Tilapia rendalli* | 6 | -0.78 | 0.92 | 0.40 | -2.59 | 1.03 | 31.48 | 95.84 | 4.81 | 4.81 | <0.001 | 1.00 | <0.001 |
| Mean corpuscular volume (fL) | Species | *Heterobranchus longifilis* | 4 | 2.03 | 1.95 | 0.30 | -1.80 | 5.85 | -0.26 | 88.30 | 12.89 | 14.29 | <0.001 | 0.33 | <0.001 |
| Melanine Concentrating Hormone | Species | *Oreochromis niloticus* | 10 | -1.19 | 0.29 | <0.001 | -1.76 | -0.62 | 17.13 | 7.69 | 0.06 | 0.44 | 0.37 | <0.001 | <0.001 |
| Melanine Concentrating Hormone | Species | *Clarias gariepinus* | 7 | 0.13 | 0.35 | 0.70 | -0.55 | 0.81 | 1535.73 | <0.001 | <0.001 | 0.23 | 0.49 | 0.24 | 0.06 |
| Melanine Concentrating Hormone | Species | *Oreochromis mossambicus* | 4 | 0.60 | 0.27 | 0.03 | 0.06 | 1.13 | 38.92 | 52.15 | 0.16 | 0.16 | 0.10 | 0.08 | 0.02 |
| Melanine Concentrating Hormone | Species | *Tilapia rendalli* | 6 | 2.23 | 0.92 | 0.01 | 0.44 | 4.03 | -1.38 | 95.35 | 4.69 | 4.69 | <0.001 | 0.02 | <0.001 |
| Melanine Concentrating Hormone | Species | *Heterobranchus longifilis* | 4 | 0.77 | 1.84 | 0.68 | -2.85 | 4.38 | 22.23 | 88.58 | 11.91 | 11.91 | <0.001 | 0.33 | 0.03 |
| Major Histocompatibility complex | Species | *Oreochromis niloticus* | 10 | -0.46 | 0.25 | 0.07 | -0.96 | 0.03 | 168.90 | <0.001 | <0.001 | <0.001 | 0.96 | <0.001 | 0.18 |
| Major Histocompatibility complex | Species | *Clarias gariepinus* | 7 | 1.18 | 0.36 | <0.001 | 0.47 | 1.89 | 12.38 | <0.001 | <0.001 | <0.001 | 0.99 | 0.24 | 0.66 |
| Major Histocompatibility complex | Species | *Oreochromis mossambicus* | 4 | 0.65 | 0.19 | <0.001 | 0.28 | 1.01 | 32.90 | <0.001 | <0.001 | <0.001 | 0.77 | 0.08 | 0.29 |
| Major Histocompatibility complex | Species | *Tilapia rendalli* | 6 | 1.10 | 0.64 | 0.08 | -0.14 | 2.35 | 12.98 | 92.65 | 2.22 | 2.22 | <0.001 | 0.14 | <0.001 |
| Major Histocompatibility complex | Species | *Heterobranchus longifilis* | 4 | -0.37 | 0.42 | 0.37 | -1.19 | 0.45 | 106.14 | <0.001 | <0.001 | <0.001 | 0.72 | 0.08 | 0.36 |
| Neutrophils | Species | *Clarias gariepinus* | 7 | 0.35 | 0.46 | 0.44 | -0.54 | 1.25 | 208.79 | 35.13 | 0.50 | 0.50 | 0.16 | 0.24 | <0.001 |
| Neutrophils | Species | *Oreochromis niloticus* | 6 | 0.15 | 0.31 | 0.63 | -0.46 | 0.76 | 1002.38 | <0.001 | <0.001 | <0.001 | 0.94 | 0.41 | 0.55 |
| Lymphocytes | Species | *Clarias gariepinus* | 7 | 0.11 | 0.61 | 0.85 | -1.08 | 1.31 | 2051.56 | 60.74 | 1.56 | 1.56 | 0.02 | 0.56 | 0.34 |
| Lymphocytes | Species | *Oreochromis niloticus* | 6 | -0.04 | 0.31 | 0.90 | -0.65 | 0.57 | 14468.91 | <0.001 | <0.001 | <0.001 | 0.99 | 0.68 | 0.71 |
| Lymphocytes | Species | *Heterobranchus longifilis* | 4 | -0.50 | 0.83 | 0.55 | -2.14 | 1.13 | 56.56 | 68.84 | 1.90 | 1.90 | 0.02 | 0.33 | 0.52 |
| Basophils | Species | *Clarias gariepinus* | 7 | <0.001 | 0.34 | 0.99 | -0.66 | 0.67 | 1746272.61 | <0.001 | <0.001 | <0.001 | 0.82 | 0.74 | 0.28 |
| Basophils | Species | *Oreochromis niloticus* | 3 | -0.14 | 0.41 | 0.74 | -0.94 | 0.66 | 610.97 | <0.001 | <0.001 | <0.001 | 0.99 | 0.33 | 0.87 |
| Basophils | Species | *Heterobranchus longifilis* | 4 | 0.19 | 0.41 | 0.64 | -0.61 | 1.00 | 405.45 | <0.001 | <0.001 | <0.001 | 0.87 | 0.08 | 0.40 |
| Monocytes | Species | *Clarias gariepinus* | 7 | 0.12 | 0.34 | 0.71 | -0.54 | 0.78 | 1763.67 | <0.001 | <0.001 | <0.001 | 0.96 | 0.40 | 0.85 |
| Monocytes | Species | *Oreochromis niloticus* | 6 | -0.01 | 0.31 | 0.98 | -0.62 | 0.60 | 468070.68 | <0.001 | <0.001 | <0.001 | 0.96 | 0.39 | 0.97 |
| Monocytes | Species | *Heterobranchus longifilis* | 4 | 1.50 | 0.47 | <0.001 | 0.58 | 2.42 | 2.83 | <0.001 | <0.001 | <0.001 | 0.67 | 0.08 | 0.22 |
| Erytrocytes | Species | *Ompok bimaculatus* | 4 | 0.52 | 0.43 | 0.22 | -0.32 | 1.35 | 53.34 | 23.95 | 0.17 | 0.17 | 0.27 | 0.33 | 0.06 |
| Erytrocytes | Species | *Clarias gariepinus* | 3 | 0.46 | 0.61 | 0.45 | -0.73 | 1.64 | 51.70 | <0.001 | <0.001 | <0.001 | 0.46 | 0.16 | 0.21 |
| Erytrocytes | Species | *Labeo rohita* | 5 | -0.49 | 3.38 | 0.89 | -7.11 | 6.14 | 75.97 | 88.90 | 22.18 | 37.41 | <0.001 | 0.19 | 0.04 |
| Threonine | Species | *Oreochromis niloticus* | 3 | 0.45 | 0.42 | 0.28 | -0.36 | 1.27 | 53.38 | <0.001 | <0.001 | <0.001 | 0.69 | 0.33 | 0.40 |
| Leukocyte | Species | *Ompok bimaculatus* | 4 | 2.66 | 0.49 | <0.001 | 1.70 | 3.62 | -1.83 | <0.001 | <0.001 | <0.001 | 0.86 | 0.08 | 0.39 |
| Leukocyte | Species | *Labeo rohita* | 5 | 5.96 | 1.78 | <0.001 | 2.47 | 9.45 | -4.46 | 77.07 | 11.42 | 11.15 | <0.001 | 0.02 | <0.001 |
| Eosinophils | Species | *Clarias gariepinus* | 4 | 0.01 | 0.41 | 0.99 | -0.80 | 0.81 | 326643.54 | <0.001 | <0.001 | <0.001 | 0.92 | 1.00 | 0.76 |
| Eosinophils | Species | *Heterobranchus longifilis* | 4 | -1.06 | 0.45 | 0.02 | -1.94 | -0.18 | 9.74 | <0.001 | <0.001 | 0.06 | 0.44 | 0.08 | 0.11 |
| Villus length (cm) | Species | *Oreochromis niloticus* | 5 | 2.36 | 0.58 | <0.001 | 1.21 | 3.50 | -1.54 | 73.94 | 1.24 | 1.24 | <0.001 | 0.02 | <0.001 |
| Villus length (cm) | Species | *Labeo rohita* | 4 | 0.31 | 0.41 | 0.45 | -0.50 | 1.12 | 152.65 | <0.001 | <0.001 | <0.001 | 0.91 | 0.08 | 0.49 |
| Villus length (cm) | Species | *Cyprinus carpio* | 3 | 1.59 | 0.90 | 0.08 | -0.17 | 3.36 | 1.54 | 86.61 | 2.10 | 2.10 | <0.001 | 0.33 | <0.001 |
| Villus length (cm) | Species | *Tilapia* sp. | 4 | -0.12 | 6.95 | 0.99 | -13.74 | 13.51 | 1146.93 | 94.94 | 169.71 | 169.71 | <0.001 | 1.00 | 0.96 |
| Villus length (cm) | Species | *Oreochromis mossambicus* | 8 | -1.63 | 0.39 | <0.001 | -2.40 | -0.86 | 3.52 | 20.97 | 0.26 | 0.89 | 0.26 | <0.001 | <0.001 |
| Villus length (cm) | Species | *Tilapia rendalli* | 8 | -0.76 | 0.30 | 0.01 | -1.35 | -0.17 | 45.33 | <0.001 | <0.001 | <0.001 | 0.97 | <0.001 | 0.18 |
| Villus length (cm) | Species | *Lateolabrax maculatus* | 5 | -0.85 | 1.28 | 0.51 | -3.36 | 1.66 | 21.59 | 88.62 | 7.24 | 7.24 | <0.001 | 0.08 | <0.001 |
| Villus length (cm) | Species | *Lateolabrax japonicus* | 3 | -0.44 | 0.41 | 0.28 | -1.26 | 0.37 | 55.24 | <0.001 | <0.001 | <0.001 | 0.94 | 0.33 | 0.72 |
| Villus length (cm) | Species | *Micropterus salmoides* | 5 | 1.17 | 0.40 | <0.001 | 0.40 | 1.95 | 8.99 | 15.86 | 0.12 | 0.12 | 0.31 | 0.08 | 0.05 |
| Villus width (cm) | Species | *Oreochromis niloticus* | 5 | -2.31 | 0.56 | <0.001 | -3.41 | -1.21 | -1.40 | 72.30 | 1.12 | 1.12 | 0.01 | 0.02 | <0.001 |
| Villus width (cm) | Species | *Labeo rohita* | 4 | -2.02 | 1.68 | 0.23 | -5.31 | 1.27 | -0.22 | 85.94 | 9.52 | 10.59 | <0.001 | 0.33 | 0.17 |
| Villus width (cm) | Species | *Tilapia* sp. | 4 | -8.13 | 4.15 | 0.05 | -16.25 | -0.01 | -3.77 | 93.99 | 59.75 | 71.51 | <0.001 | 0.08 | <0.001 |
| Villus width (cm) | Species | *Oreochromis mossambicus* | 8 | 0.05 | 0.29 | 0.86 | -0.52 | 0.62 | 12484.78 | <0.001 | <0.001 | <0.001 | 1.00 | 0.01 | 0.84 |
| Villus width (cm) | Species | *Tilapia rendalli* | 8 | 0.08 | 0.29 | 0.79 | -0.49 | 0.64 | 5229.65 | <0.001 | <0.001 | <0.001 | 1.00 | 0.03 | 0.75 |
| Villus width (cm) | Species | *Lateolabrax maculatus* | 5 | 1.45 | 1.77 | 0.41 | -2.01 | 4.91 | 4.12 | 91.56 | 14.22 | 14.22 | <0.001 | 0.08 | <0.001 |
| Villus width (cm) | Species | *Lateolabrax japonicus* | 3 | 1.06 | 0.44 | 0.02 | 0.20 | 1.91 | 7.30 | <0.001 | <0.001 | <0.001 | 0.87 | 0.33 | 0.60 |
| Villus width (cm) | Species | *Micropterus salmoides* | 5 | 0.41 | 0.33 | 0.22 | -0.24 | 1.07 | 108.44 | <0.001 | <0.001 | <0.001 | 0.57 | 0.82 | 0.16 |
| Villi density (mm²) | Species | *Oreochromis niloticus* | 5 | 3.00 | 0.39 | <0.001 | 2.23 | 3.77 | -2.87 | 28.69 | 0.22 | 0.41 | 0.23 | 0.02 | 0.02 |
| Villi density (mm²) | Species | *Rhamdia quelen* | 5 | -0.28 | 0.23 | 0.21 | -0.72 | 0.16 | 236.01 | <0.001 | <0.001 | <0.001 | 0.87 | 0.02 | 0.28 |
| Muscular thickness (cm) | Species | *Lateolabrax japonicus* | 3 | 0.23 | 0.41 | 0.57 | -0.57 | 1.03 | 213.37 | <0.001 | <0.001 | <0.001 | 0.97 | 0.33 | 0.80 |
| Muscular thickness (cm) | Species | *Micropterus salmoides* | 4 | -0.17 | 0.36 | 0.64 | -0.87 | 0.53 | 525.91 | <0.001 | <0.001 | <0.001 | 0.62 | 0.33 | 0.24 |
| Goblet cell number | Species | *Cyprinus carpio* | 3 | 2.15 | 1.23 | 0.08 | -0.26 | 4.55 | -0.50 | 91.18 | 4.09 | 4.09 | <0.001 | 0.33 | <0.001 |
| Goblet cell number | Species | *Oreochromis mossambicus* | 8 | 1.31 | 0.38 | <0.001 | 0.57 | 2.05 | 9.92 | 20.94 | 0.24 | 0.56 | 0.26 | <0.001 | <0.001 |
| Goblet cell number | Species | *Lateolabrax japonicus* | 3 | -0.66 | 0.42 | 0.12 | -1.49 | 0.16 | 23.10 | <0.001 | <0.001 | <0.001 | 0.70 | 0.33 | 0.40 |
| Albumin (g/L) | Species | *Oreochromis niloticus* | 7 | -0.31 | 0.32 | 0.33 | -0.94 | 0.32 | 274.93 | <0.001 | <0.001 | <0.001 | 0.56 | 0.14 | 0.13 |
| Albumin (g/L) | Species | *Labeo rohita* | 6 | -0.10 | 0.50 | 0.84 | -1.07 | 0.87 | 2208.32 | 47.26 | 0.69 | 0.69 | 0.09 | 0.72 | 0.15 |
| Albumin (g/L) | Species | *Ctenopharyngodon idella* | 9 | -0.85 | 0.59 | 0.15 | -2.01 | 0.30 | 38.32 | 71.15 | 2.11 | 2.11 | <0.001 | 0.34 | 0.05 |
| Albumin (g/L) | Species | *Oreochromis mossambicus* | 4 | 0.72 | 0.27 | 0.01 | 0.18 | 1.26 | 25.38 | 51.74 | 0.16 | 0.16 | 0.10 | 0.08 | 0.02 |
| Albumin (g/L) | Species | *Clarias gariepinus* | 3 | 0.85 | 0.62 | 0.17 | -0.37 | 2.07 | 13.10 | <0.001 | <0.001 | <0.001 | 0.48 | 0.33 | 0.24 |
| Albumin (g/L) | Species | *Lateolabrax maculatus* | 10 | -0.22 | 0.56 | 0.69 | -1.31 | 0.87 | 760.96 | 77.09 | 2.27 | 2.27 | <0.001 | 1.00 | 0.02 |
| Albumin (g/L) | Species | *Lateolabrax japonicus* | 3 | -0.07 | 0.41 | 0.86 | -0.88 | 0.73 | 2093.67 | <0.001 | <0.001 | <0.001 | 0.90 | 1.00 | 0.66 |
| Globulin (g/L) | Species | *Oreochromis niloticus* | 7 | -0.10 | 0.41 | 0.81 | -0.91 | 0.71 | 2792.52 | 36.41 | 0.43 | 0.43 | 0.15 | 0.77 | 0.02 |
| Globulin (g/L) | Species | *Labeo rohita* | 6 | -0.33 | 1.51 | 0.83 | -3.29 | 2.63 | 205.05 | 85.54 | 9.23 | 12.66 | <0.001 | 0.27 | 0.01 |
| Globulin (g/L) | Species | *Clarias gariepinus* | 3 | -1.86 | 1.08 | 0.08 | -3.98 | 0.25 | 0.31 | 48.88 | 1.68 | 1.68 | 0.14 | 0.33 | 0.05 |
| Globulin (g/L) | Species | *Lateolabrax maculatus* | 10 | 0.52 | 0.23 | 0.03 | 0.06 | 0.97 | 134.66 | <0.001 | <0.001 | <0.001 | 0.80 | <0.001 | 0.08 |
| Globulin (g/L) | Species | *Lateolabrax japonicus* | 3 | -0.06 | 0.41 | 0.88 | -0.88 | 0.75 | 2733.35 | <0.001 | <0.001 | <0.001 | 0.54 | 1.00 | 0.28 |
| Globulin (g/L) | Species | *Ctenopharyngodon idella* | 4 | 0.10 | 2.32 | 0.97 | -4.44 | 4.64 | 1517.30 | 92.67 | 19.68 | 19.68 | <0.001 | 0.70 | 0.51 |
| High-density lipoprotein cholesterol (mmol/L) | Species | *Oreochromis niloticus* | 3 | -1.23 | 0.52 | 0.02 | -2.26 | -0.21 | 4.58 | <0.001 | <0.001 | 0.11 | 0.60 | 0.33 | 0.32 |
| High-density lipoprotein cholesterol (mmol/L) | Species | *Lateolabrax maculatus* | 10 | 0.64 | 0.48 | 0.18 | -0.30 | 1.58 | 84.24 | 71.39 | 1.60 | 1.60 | <0.001 | 0.17 | <0.001 |
| High-density lipoprotein cholesterol (mmol/L) | Species | *Lateolabrax japonicus* | 8 | 0.20 | 0.25 | 0.42 | -0.29 | 0.70 | 735.26 | <0.001 | <0.001 | <0.001 | 0.97 | 0.04 | 0.29 |
| High-density lipoprotein cholesterol (mmol/L) | Species | *Ctenopharyngodon idella* | 3 | -7.20 | 1.13 | <0.001 | -9.43 | -4.98 | -2.78 | <0.001 | <0.001 | 1.88 | 0.49 | 0.33 | 0.23 |
| Low-density lipoprotein cholesterol (mmol/L) | Species | *Oreochromis niloticus* | 3 | -1.21 | 0.63 | 0.06 | -2.45 | 0.04 | 4.93 | 28.84 | 0.35 | 1.66 | 0.25 | 0.33 | 0.10 |
| Low-density lipoprotein cholesterol (mmol/L) | Species | *Lateolabrax maculatus* | 10 | 0.38 | 0.23 | 0.10 | -0.07 | 0.83 | 254.46 | <0.001 | <0.001 | <0.001 | 0.63 | 0.01 | 0.03 |
| Low-density lipoprotein cholesterol (mmol/L) | Species | *Lateolabrax japonicus* | 8 | 0.80 | 0.57 | 0.16 | -0.32 | 1.93 | 39.55 | 74.05 | 1.88 | 1.88 | <0.001 | 0.08 | <0.001 |
| Low-density lipoprotein cholesterol (mmol/L) | Species | *Ctenopharyngodon idella* | 3 | -5.49 | 4.47 | 0.22 | -14.26 | 3.28 | -2.62 | 94.10 | 55.75 | 55.75 | <0.001 | 0.33 | <0.001 |
| TNF-α (ng/L) | Species | *Lateolabrax japonicus* | 8 | -1.62 | 0.29 | <0.001 | -2.19 | -1.05 | 3.72 | <0.001 | <0.001 | <0.001 | 0.95 | <0.001 | 0.14 |
| Nuclear Factor | Species | *Ctenopharyngodon idella* | 3 | 2.35 | 0.53 | <0.001 | 1.31 | 3.40 | -0.92 | <0.001 | <0.001 | <0.001 | 0.92 | 0.33 | 0.68 |
| Keap1 | Species | *Ctenopharyngodon idella* | 3 | 6.77 | 1.28 | <0.001 | 4.26 | 9.28 | -2.75 | 26.72 | 1.37 | 5.76 | 0.26 | 0.33 | 0.10 |
| IL-6 (ng/L) | Species | *Lateolabrax japonicus* | 8 | -0.93 | 0.67 | 0.17 | -2.24 | 0.39 | 27.91 | 79.50 | 2.74 | 3.31 | <0.001 | 0.26 | <0.001 |
| IL-8 (ng/L) | Species | *Lateolabrax japonicus* | 8 | 0.69 | 0.26 | 0.01 | 0.18 | 1.20 | 57.20 | <0.001 | <0.001 | <0.001 | 0.83 | <0.001 | 0.07 |
| IL-8 (ng/L) | Species | *Ctenopharyngodon idella* | 3 | 2.01 | 0.50 | <0.001 | 1.02 | 2.99 | -0.13 | <0.001 | <0.001 | <0.001 | 0.89 | 0.33 | 0.62 |
| IL-10 (ng/L) | Species | *Lateolabrax japonicus* | 5 | 0.36 | 0.32 | 0.26 | -0.27 | 0.99 | 141.49 | <0.001 | <0.001 | <0.001 | 0.90 | 0.02 | 0.33 |
| IL-10 (ng/L) | Species | *Ctenopharyngodon idella* | 3 | 3.26 | 0.93 | <0.001 | 1.45 | 5.08 | -1.92 | 50.03 | 1.27 | 1.27 | 0.14 | 0.33 | 0.05 |
| Immune globulin (µg/mg) | Species | *Oreochromis niloticus* | 7 | -0.25 | 0.29 | 0.38 | -0.82 | 0.32 | 412.89 | <0.001 | <0.001 | <0.001 | 1.00 | 0.76 | 0.76 |
| Immune globulin (µg/mg) | Species | *Lateolabrax maculatus* | 4 | 2.14 | 0.45 | <0.001 | 1.26 | 3.03 | -0.65 | <0.001 | <0.001 | 0.11 | 0.58 | 0.08 | 0.16 |
| Immune globulin (µg/mg) | Species | *Lateolabrax japonicus* | 4 | 1.04 | 0.40 | 0.01 | 0.27 | 1.82 | 10.13 | <0.001 | <0.001 | <0.001 | 0.60 | 0.08 | 0.23 |

*g* – Hedges’ *g*; *p* – significance value for the pooled effect size, Q – significance value from Cochran’s Q test for heterogeneity, Begg – significance value from Begg’s test, Egger – significance value from Egger’s regression test, FSN – fail state number, cm – centimetre, cm^3^ – cubic centimetre, dL – decilitre, fL – femtoliter, FSN – fail state number, HDLC – high density lipoprotein cholesterol, kU – kilounit, µg – microgram, g – gram, IL – interleukin, *I²* – heterogeneity, *τ²* – estimated between-study variance from the original random-effects model; *τ²′* – estimated between-study variance from the corresponding adjusted/prime random-effects model; K+ - Kalium, k – number of studies, IU – international unit, L – litre, mg – milligram, LDLC – low density lipoprotein cholesterol, mL – mililiter, mmol – milimol, mm^2^ – square millimetre, Na+ - natrium, ng – nanogram, pg – pictogram, U – unit, Qs – significance value from the Q statistic; SE – standard error from the random effects model, TNF-α – Tumor Necrosis Factor-alpha, % – percentage, µmol – micromole
